# Rb-driven transcription limits its tumour-suppressive effects in breast cancer

**DOI:** 10.1038/s41586-026-10886-w

**Published:** 2026-08-12

**Authors:** April C. Watt, Antonio Ahn, Catherine Blyth, Julia R. Dixon-Douglas, Krutika Ambani, Rhiannon Coulson, Michael Taylor, Keefe T. Chan, Catherine Dietrich, Brendan E. Russ, Susanne Ramm, Christabella A. Mahendra, Kun-Hui Lu, Nichelle Pires, Jesus Garcia-Sannicolas, Olivia Voulgaris, Sheena Nunag, Ching-Seng Ang, Mark A. Dawson, Elgene Lim, Monica Arnedos, Sarat Chandarlapaty, Fabrice André, Shom Goel

**Affiliations:** 1 Peter MacCallum Cancer Centre, Melbourne, Victoria Australia; 2 Sir Peter MacCallum Department of Oncology, University of Melbourne, Parkville, Victoria Australia; 3 INSERM U981, Molecular Predictors and Novel Targets in Oncology, Gustave Roussy, Villejuif, France; 4 Department of Medical Oncology, Gustave Roussy, Villejuif, France; 5 Garvan Institute of Medical Research, Sydney, New South Wales Australia; 6 School of Clinical Medicine, St Vincent’s Healthcare Clinical Campus, Faculty of Medicine and Health, University of New South Wales, Sydney, New South Wales Australia; 7 Bio21 Mass Spectrometry and Proteomics Facility, University of Melbourne, Parkville, Victoria Australia; 8 Collaborative Centre for Genomic Cancer Medicine, University of Melbourne, Parkville, Victoria Australia; 9 Department of Medicine, Institut Bergonie, Bordeaux, France; 10 INSERM U1312, BRIC, Université Bordeaux, Bordeaux, France; 11 Human Oncology and Pathogenesis Program, Memorial Sloan Kettering Cancer Center, New York, NY USA; 12 Department of Medicine, Memorial Sloan Kettering Cancer Center, New York, NY USA; 13 Faculty of Medicine, Université Paris-Saclay, Le Kremlin-Bicêtre, France

**Keywords:** Breast cancer, Cell-cycle exit, Tumour-suppressor proteins, Epigenetics

## Abstract

The retinoblastoma protein (Rb) is a tumour suppressor best known for repressing E2F transcription factors and halting cell cycle progression^1^. In hormone receptor-positive (HR^+^) breast cancer, CDK4/6 inhibitors activate Rb by preventing its phosphorylation, forming a key component of current endocrine therapy regimens^2^. How pharmacologically activated Rb remodels chromatin and influences transcription beyond cell cycle arrest remains poorly understood. Here we show that CDK4/6 inhibition induces redistribution of hypophosphorylated Rb to promoters and enhancers. Although Rb predictably binds to cell cycle gene promoters to repress transcription, at other sites, it unexpectedly promotes expression of oestrogen-responsive genes by integrating into oestrogen receptor (ER)-rich transcriptional hubs. CDK4/6 inhibition enhances ER target gene expression in breast cancer cells, patient-derived xenografts and clinical HR^+^ breast cancer samples in an Rb-dependent manner. This reprogramming is mediated in part by KDM5A, whose interaction with Rb contributes to gene regulation at these loci. Critically, components of this Rb-driven ER transcriptional program are pro-proliferative. In endocrine-sensitive tumours, this effect can be neutralized with anti-oestrogen therapy, explaining therapeutic synergy. In endocrine-resistant settings such as *ESR1*-mutant breast cancer, the program persists, limiting the therapeutic efficacy of CDK4/6 inhibition. These findings reframe Rb as a dual-function transcriptional regulator that, although enforcing cell cycle arrest, can also activate programs that counteract its tumour suppressor function.

## Abstract (web summary)

CDK4/6 inhibition redirects Rb chromatin occupancy to activate oestrogen-responsive genes, counteracting its own ability to maintain therapeutic cell-cycle arrest.

## Main

The retinoblastoma protein is a tumour suppressor that prevents uncontrolled cell division^1^. It enforces G1 cell cycle arrest by binding to and antagonizing E2F transcription factors at gene promoters, thereby blocking expression of E2F target genes that are required for S-phase entry^3–6^. This repression is mediated by hypophosphorylated (‘active’) Rb^7,8^. During G1, cyclin-dependent kinases (CDKs)—specifically, CDK2, CDK4 and CDK6—phosphorylate Rb. Once hyperphosphorylated (‘inactive’), Rb releases E2F factors, driving irreversible S-phase commitment^7^.

Many cancer cells retain wild-type Rb expression, and the development of selective CDK inhibitors has positioned Rb as a critical therapeutic target. Selective CDK inhibitors enforce Rb hypophosphorylation, downregulate E2F target genes and induce G1 arrest^2^. Clinically, CDK4/6 inhibitors effectively suppress Rb phosphorylation and tumour proliferation in HR^+^ breast cancers, forming the cornerstone of first-line therapy in early and advanced disease^9^. CDK2 inhibitors are similarly promising for tumours driven by increased CDK2 activity^10^.

Beyond E2F repression, hypophosphorylated Rb probably has broader functions on chromatin. Studies from the pre-genomic era suggested that active Rb can interact with other transcription factors to modulate their activity^11–15^. Thus, pharmacologically activated Rb might not only halt cell division but also influence broader transcriptional programs that affect therapeutic outcomes. Despite widespread clinical use of CDK4/6 inhibitors, the downstream transcriptional consequences of sustained Rb activation in cancer remain poorly defined. Clarifying these effects could enhance CDK inhibitor use and elucidate resistance mechanisms.

Historically, mapping the genome-wide chromatin occupancy of Rb has proven difficult because of technical challenges associated with chromatin immunoprecipitation followed by sequencing (ChIP–seq), particularly the low signal-to-noise ratio typically observed^8^. Attempts to circumvent this problem have used exogenously expressed, tagged Rb^16^, an approach that might be insensitive to the marked cell cycle-dependent fluctuations in endogenous Rb abundance^17^ and prone to artefacts from tag-induced alterations in protein function, chromatin-binding specificity and cross-reactivity with off-target proteins. Moreover, most previous Rb ChIP–seq studies have been conducted in non-transformed cells, leaving the effect of CDK inhibition on the chromatin-binding landscape and transcriptional activity of Rb in cancer cells unresolved.

## Drug-activated Rb accumulates on chromatin

**Fig. 1:**
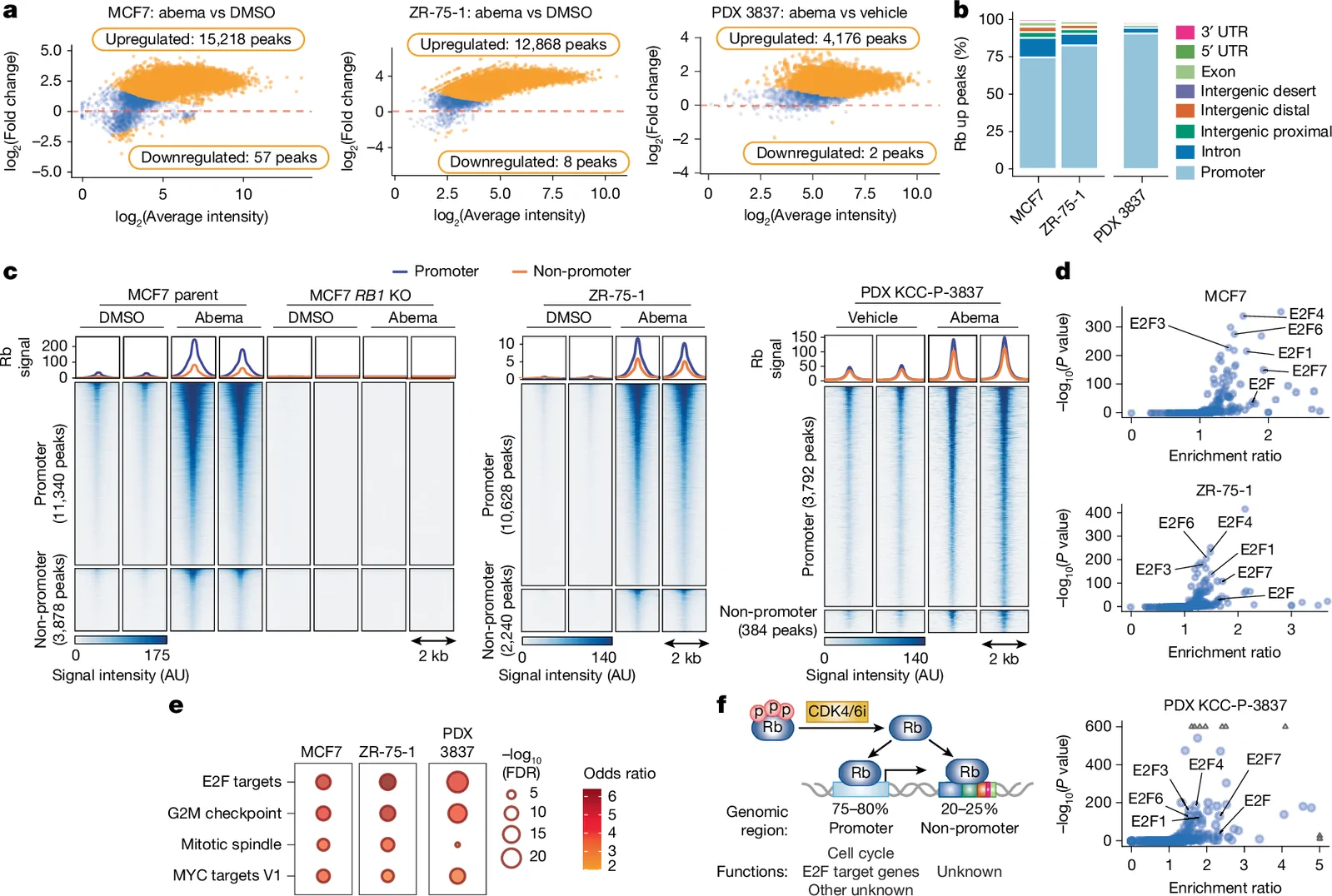
CDK4/6 inhibition drives genome-wide redistribution of native Rb to chromatin. Rb chromatin binding was profiled by CUT&RUN in MCF7 and ZR-75-1 cells treated with abemaciclib (abema) or DMSO, and by DynaTag in the breast cancer PDX model KCC-P-3837 treated with abemaciclib or vehicle. **a**, MA (minus-average) plots of differential Rb chromatin binding following abemaciclib treatment. Each dot represents a 400-bp genomic region. Differential Rb binding was determined using DESeq2 with Benjamini–Hochberg multiple-testing correction; orange indicates regions with adjusted *P* < 0.1 for cell lines and adjusted *P* < 0.2 for the PDXs; horizontal dashed lines indicate log_2_(fold change) = 0. **b**, Genomic distribution of regions showing increased Rb binding (up peaks) identified in **a**. UTR, untranslated region. **c**, Composite profiles and heatmaps of the Rb signal across up-peak regions defined in **b**. Each row represents a genomic region; the colour indicates signal intensity. AU, arbitrary units. **d**, HOMER motif enrichment analysis of promoter-associated Rb up peaks identified in **b**, where *P* values were calculated by HOMER using a cumulative hypergeometric test. **e**, ChIP-Enrich analysis of promoter-associated Rb up peaks. The top three hallmark gene sets ranked by adjusted *P* values in any of the models using the ChIP-Enrich logistic regression model with Benjamini–Hochberg correction are shown; only data points with adjusted *P* < 0.05 are displayed. FDR, false discovery rate. **f**, Model depicting redistribution of hypophosphorylated Rb to promoter-associated chromatin and other genomic regions following CDK4/6 inhibitor (CDK4/6i) treatment. Unless otherwise indicated, CUT&RUN and DynaTag experiments were performed using *n* = 2 independent biological cultures or individual tumours per condition, with independent chromatin profiling reactions for each replicate. Source data

**Extended Data Fig. 1:**
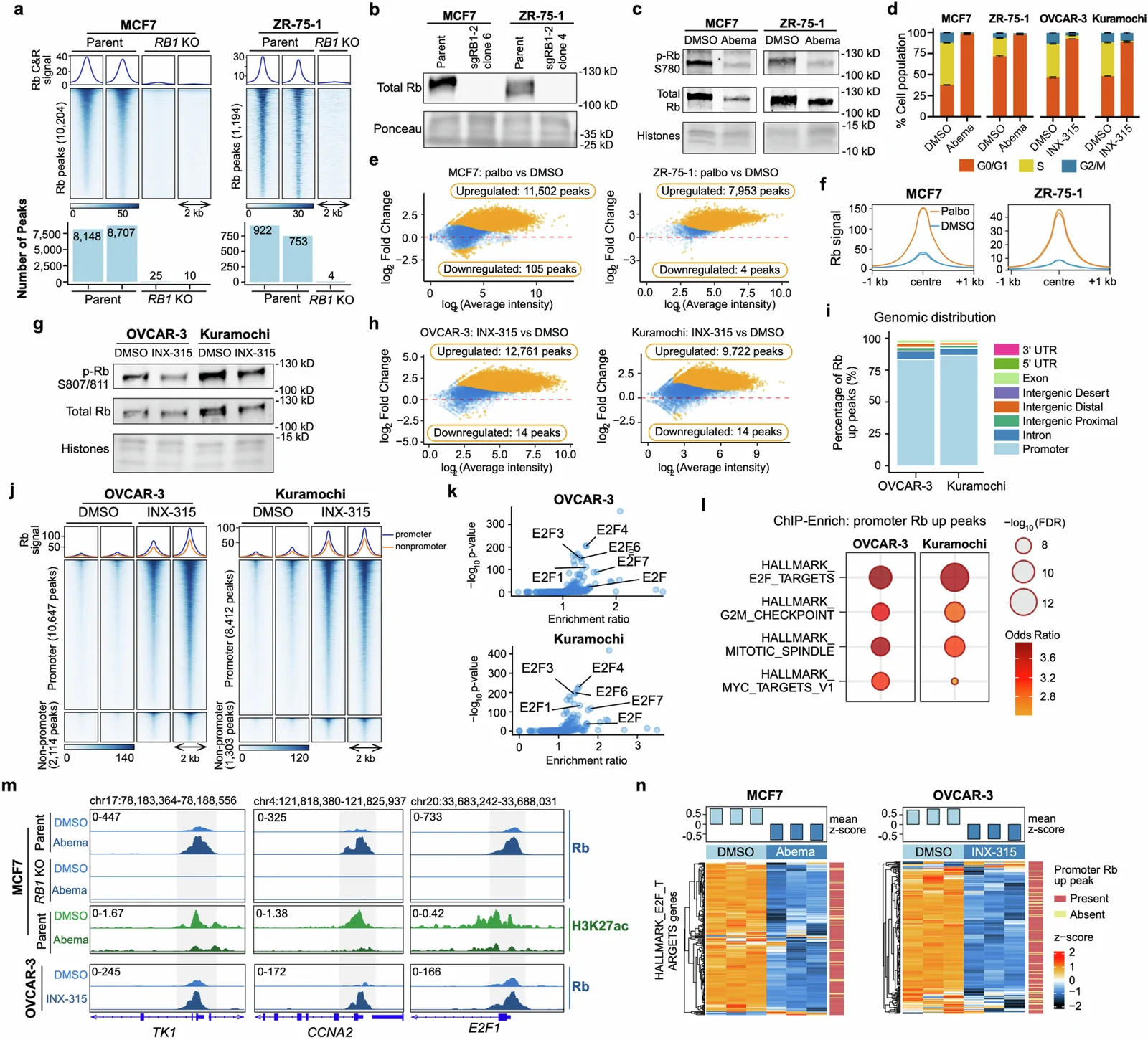
Inhibition of CDKs redistributes Rb to chromatin in breast and ovarian cancer cells. **a**, Composite profiles and heatmaps of Rb CUT&RUN signal in all peaks detected in parental or isogenic *RB1* knockout cells. Each row represents a genomic region; colour indicates signal intensity. Bar plots indicate the number of Rb peaks identified in each sample. **b, c, g**, Immunoblot analysis of total Rb in parental and *RB1* knockout cells (**b**), and of total and phosphorylated Rb following abemaciclib (**c**) or INX-315 (**g**) treatment. **d**, Cell-cycle distribution following treatment as indicated. Data represent mean ± s.d.; *n* = 3 individual cultures per condition. **e, h**, MA plots of differential Rb CUT&RUN peaks following palbociclib (**e**) or INX-315 (**h**) treatment. Each point represents a 400-bp genomic region. Differential Rb binding was determined using DESeq2 with Benjamini-Hochberg multiple-testing correction; orange indicates adjusted P < 0.1. **f**, Composite profiles of Rb occupancy in cells treated with DMSO or palbociclib across genomic regions with abemaciclib-induced Rb up peaks. **i**, Genomic annotation of Rb up peaks identified following INX-315 treatment. **j**, Composite profiles and heatmaps of Rb CUT&RUN signal across Rb up peaks identified in **h**. **k**, **l**, HOMER motif enrichment analysis (k) and ChIP-Enrich analysis (l) of promoter-associated Rb up peaks identified following INX-315 treatment, where P values calculated by HOMER using a cumulative hypergeometric test. **m**, Rb CUT&RUN (blue) and H3K27ac ChIP-seq (green) signal tracks at representative E2F target gene promoters. Grey boxes indicate the Rb up peaks at promoter loci. **n**, Heatmaps of *Hallmark E2F Targets* gene expression in cells treated with CDK inhibitors or DMSO for 5 days. Rows represent individual genes and columns represent biological replicates. Bar plots indicate mean z-score in each sample. The annotation bars to the right indicate presence or absence of a promoter Rb up peak. CUT&RUN experiments were performed using *n* = 2 independent biological cultures per condition, except for the ZR-75-1 *RB1* knockout sample in **a** (*n* = 1), and RNA-seq was performed on *n* = 3 independent biological cultures per condition. For gel source data, see Supplementary Fig. 1. *RB1* KO, isogenic *RB1* knockout cells; Abema, abemaciclib; Palbo, palbociclib. Source data

To overcome longstanding challenges in profiling Rb–chromatin interactions, we applied cleavage under targets and release using nuclease (CUT&RUN)^18^, enabling high-resolution mapping of native, untagged Rb without the need for formaldehyde crosslinking. Using validated monoclonal antibodies, we optimized this assay in HR^+^ breast cancer cell lines (MCF7 and ZR-75-1), generating distinct Rb peaks, which were absent in isogenic *RB1*-knockout cells, confirming specificity (Extended Data Fig. 1a,b). We applied our Rb CUT&RUN assay to examine how CDK inhibition alters Rb chromatin occupancy. Upon CDK4/6 inhibition with abemaciclib, both cell lines exhibited Rb hypophosphorylation, reduced total Rb levels and G1 arrest, as expected (Extended Data Fig. 1c,d). Critically, genome-wide analysis revealed a substantial increase in chromatin-bound Rb—15,218 and 12,868 ‘up peaks’ and only 57 and 8 ‘down peaks’ in MCF7 and ZR-75-1 cells, respectively—highlighting the dramatic chromatin redistribution of hypophosphorylated, active Rb despite reduced total protein levels (Fig. 1a). These observations were confirmed with palbociclib, a different CDK4/6 inhibitor, and extended to CDK2-inhibited cyclin E-driven ovarian cancer lines, suggesting broader relevance^10^ (Extended Data Fig. 1d–h).

Approximately 75–80% of induced Rb peaks localized to gene promoters (Fig. 1b,c and Extended Data Fig. 1i,j). Promoter Rb up peaks were enriched with E2F motifs and associated with cell cycle genes (Fig. 1d,e, Extended Data Fig. 1k–m and Supplementary Table 1). The expression of classical cell cycle genes was substantially downregulated; consistent with the known role of Rb in recruiting histone deacetylases to cell cycle promoters^19,20^, H3K27 acetylation, a mark of transcriptional activity, was reduced at these promoters (Extended Data Fig. 1m,n). Of note, we also observed extensive Rb accumulation at non-promoter regions, including intergenic and intronic loci. This unexpected redistribution hinted at uncharacterized roles for Rb in transcriptional regulation beyond E2F repression, which is explored in subsequent sections.

**Extended Data Fig. 2:**
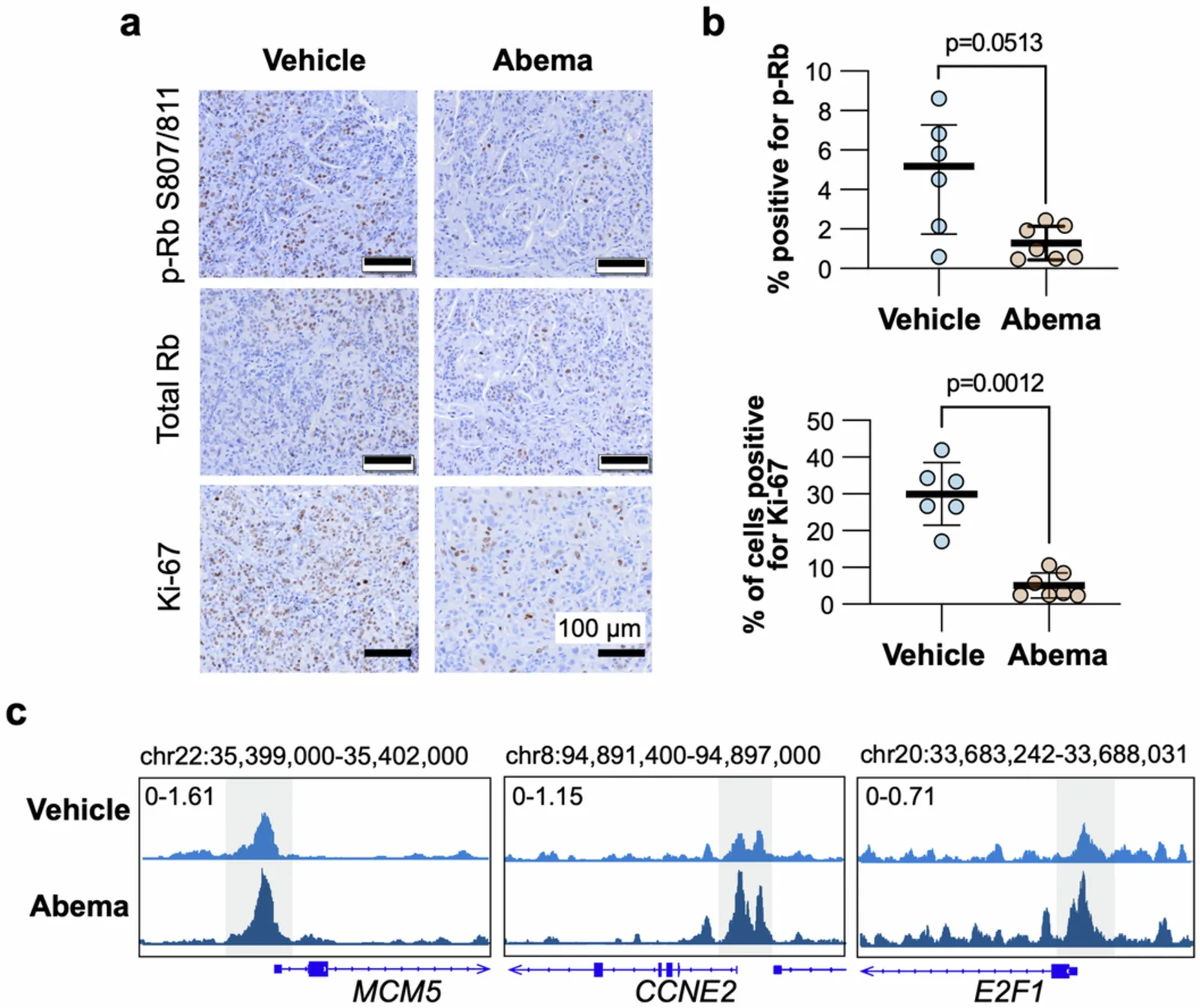
Cell cycle markers in a breast cancer patient-derived xenograft model. **a**, Representative immunohistochemistry images of KCC-P-3837 patient-derived xenograft (PDX) tumour sections stained for phosphorylated Rb (p-Rb), total Rb, and Ki-67. **b**, Quantification of immunohistochemistry staining by percentage of p-Rb-positive (top) or Ki-67-positive (bottom) cells. One image was analysed per tumour; *n* = 6 tumours for vehicle-treated mice and *n* = 7 for abemaciclib-treated mice. Points represent individual tumours. Centre values indicate the mean and error bars denote s.d. *P* values were calculated using two-tailed Mann-Whitney test. **c**, Representative Rb chromatin occupancy profiles in KCC-P-3837 tumours at E2F target gene promoter regions. Grey boxes indicate promoter-associated Rb up peaks. Abema, abemaciclib. Source data

To extend these findings in vivo, we profiled chromatin occupancy of native Rb in a patient-derived xenograft (PDX) of breast cancer treated with abemaciclib. For these experiments, we used dynamic targets and tagmentation (DynaTag), a modified method of CUT&Tag^21^, which provided an improved signal-to-noise ratio in tumour tissue compared with CUT&RUN. Abemaciclib reduced Rb phosphorylation and tumour cell proliferation in vivo, as expected (Extended Data Fig. 2a,b). Consistent with our in vitro observations, essentially all differential Rb signals were up peaks, which localized to promoters enriched for cell cycle genes (Fig. 1a–e and Extended Data Fig. 2c). Together, these results illustrate that epigenetic profiling of native Rb faithfully captures known functions of Rb at a genome-wide scale, while enabling investigation of previously inaccessible aspects of Rb biology at promoter and non-promoter regions (Fig. 1f).

## Rb engages enhancer networks

**Extended Data Fig. 3:**
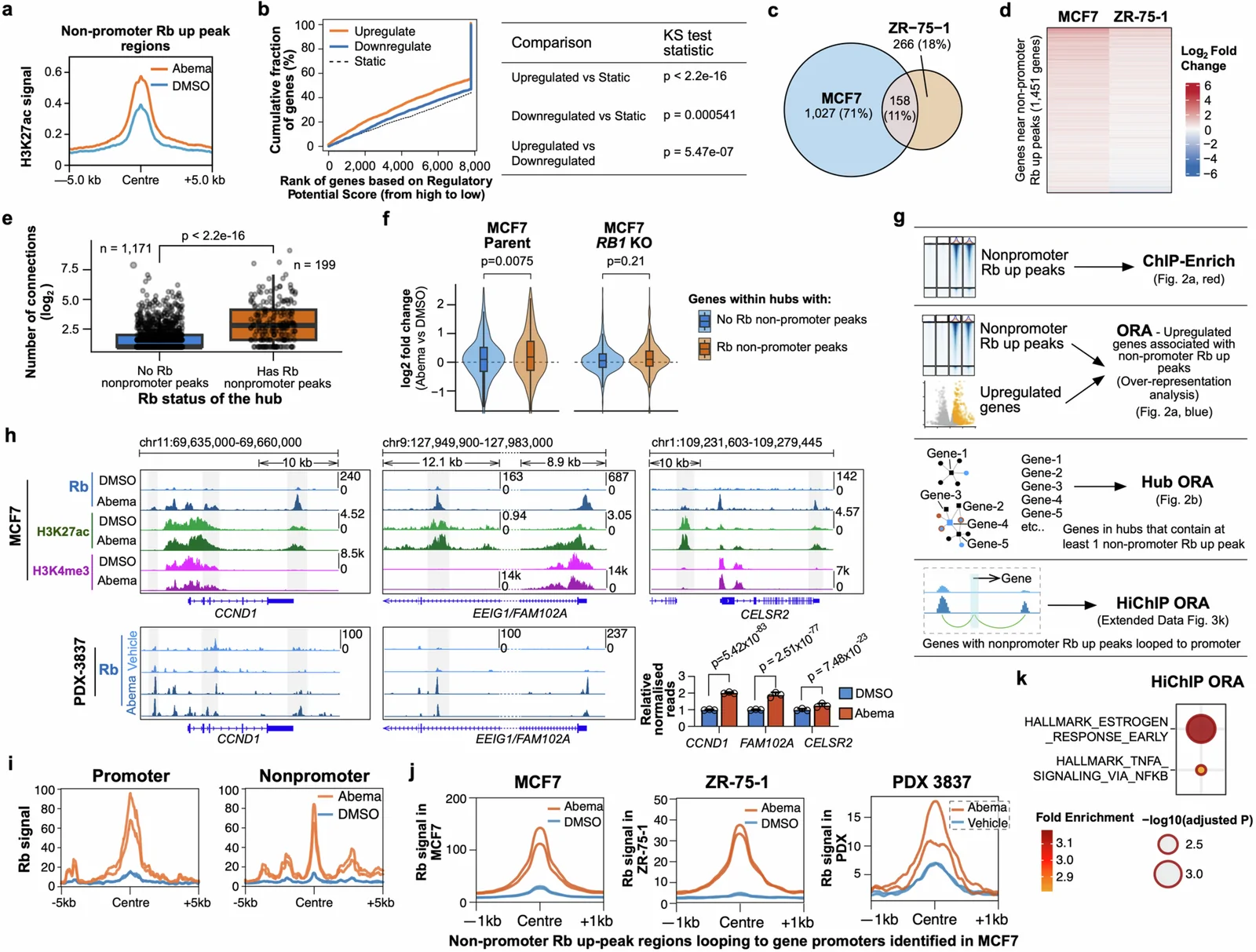
CDK4/6 inhibition induces Rb to regulatory regions surrounding oestrogen-responsive genes. **a**, Composite H3K27ac ChIP-seq signal at non-promoter Rb up-peaks in MCF7 cells (*n* = 1 replicate per condition). **b**, Binding and Expression Target Analysis (BETA) of non-promoter Rb up peaks and gene expression following abemaciclib treatment in MCF7 cells. Statistical significance was determined using a one-sided Kolmogorov-Smirnov test. **c, d**, Overlap (c) and relative expression (d) of abemaciclib-upregulated genes associated with non-promoter Rb up peaks across two cell lines as defined in the ORA analysis shown in Fig. 2a. **e**, Number of chromatin interactions per H3K27ac HiChIP transcriptional hub with or without at least one non-promoter Rb up peak in abemaciclib-treated MCF7 cells. Each point represents one hub; boxplots: centre line denotes median; boxes, interquartile range; whiskers, ±1.5× IQR. Two-tailed Wilcoxon rank-sum test. **f**, Expression changes following abemaciclib treatment for genes residing in transcriptional hubs containing at least one non-promoter Rb up-peak (orange; 464 genes) or lacking such sites (blue; 1,552 genes). Boxplots: centre line denotes median; boxes, interquartile range; whiskers, ±1.5× IQR. Two-sided *t*-test. **g**, Schematic illustrating gene set enrichment strategies used in Fig. 2a,b and k. **h**, Top and bottom left, representative genomic regions near oestrogen-responsive genes. Bottom right, RNA-seq expression of the corresponding genes following abemaciclib treatment in MCF7 cells. Data represent mean ± s.d. Adjusted *P* values from DESeq2 (two-sided Wald test with Benjamini-Hochberg correction for multiple testing). **i**, **j**, Composite Rb chromatin occupancy profiles in MCF7 cells at promoter regions of 55 *Hallmark Estrogen Response Early* genes identified by ORA in Fig. 2a and at their associated 92 non-promoter Rb up peaks located within 100 kb of these genes (i) and in cell lines and the PDX model across 405 non-promoter Rb up-peak regions that are linked to gene promoters by H3K27ac HiChIP interactions (j). **k**, *Hallmark* ORA of genes linked to non-promoter Rb up peaks by H3K27ac HiChIP in abemaciclib-treated MCF7 cells. Enrichment significance was assessed using a one-sided hypergeometric test and Benjamini-Hochberg correction for multiple testing. Only gene sets with adjusted *P* < 0.05 are shown. Chromatin profiling, *n* = 2 independent biological samples or tumours per condition; RNA-seq, *n* = 3 biologically independent samples per condition. *RB1* KO, isogenic *RB1* knockout cells; Abema, abemaciclib. Values shown as p < 2.2 × 10^−^^16^ correspond to the lower reporting limit of R. Source data

To explore functions of chromatin-bound Rb beyond its classical activity at cell cycle gene promoters, we initially focused on non-promoter regions where CDK4/6 inhibition increased Rb occupancy: regions distinct from canonical Rb–E2F interactions with poorly defined regulatory roles. As HR^+^ breast cancer is the primary clinical context in which CDK inhibitors are used to activate Rb, we focused on this disease setting. In MCF7 and ZR-75-1 cells, abemaciclib treatment induced 3,878 and 2,240 Rb up peaks, respectively, outside annotated promoters (Fig. 1c). In contrast to classical cell cycle gene promoters, these sites were marked by increased levels of H3K27ac (acetylation of lysine 27 on histone H3) and were significantly associated with upregulation of nearby genes (via binding and expression target analysis (BETA) analysis^22^), suggesting that they may represent pre-existing enhancers that gain additional activity upon Rb recruitment (Extended Data Fig. 3a–d).

To further examine possible functions of Rb at these non-classical sites, we analysed our previously published H3K27ac high-throughput chromatin conformation capture combined with chromatin immunoprecipitation (HiChIP) data from abemaciclib-treated MCF7 cells^23^ (of note, the HiChIP data were generated after 7 days of treatment, whereas Rb CUT&RUN profiling in the current study was performed after 2 days, a difference in treatment duration that represents a limitation of this integrative analysis). Transcriptional hubs—3D networks of H3K27ac-marked loci connected by chromatin interactions—were identified from HiChIP contacts. Hubs containing non-promoter Rb peaks were more complex, exhibiting a greater number of H3K27ac-marked nodes and interconnections than those lacking Rb (Extended Data Fig. 3e). These hubs also showed increased transcriptional activity, which was attenuated by *RB1* knockout (Extended Data Fig. 3f).

**Fig. 2:**
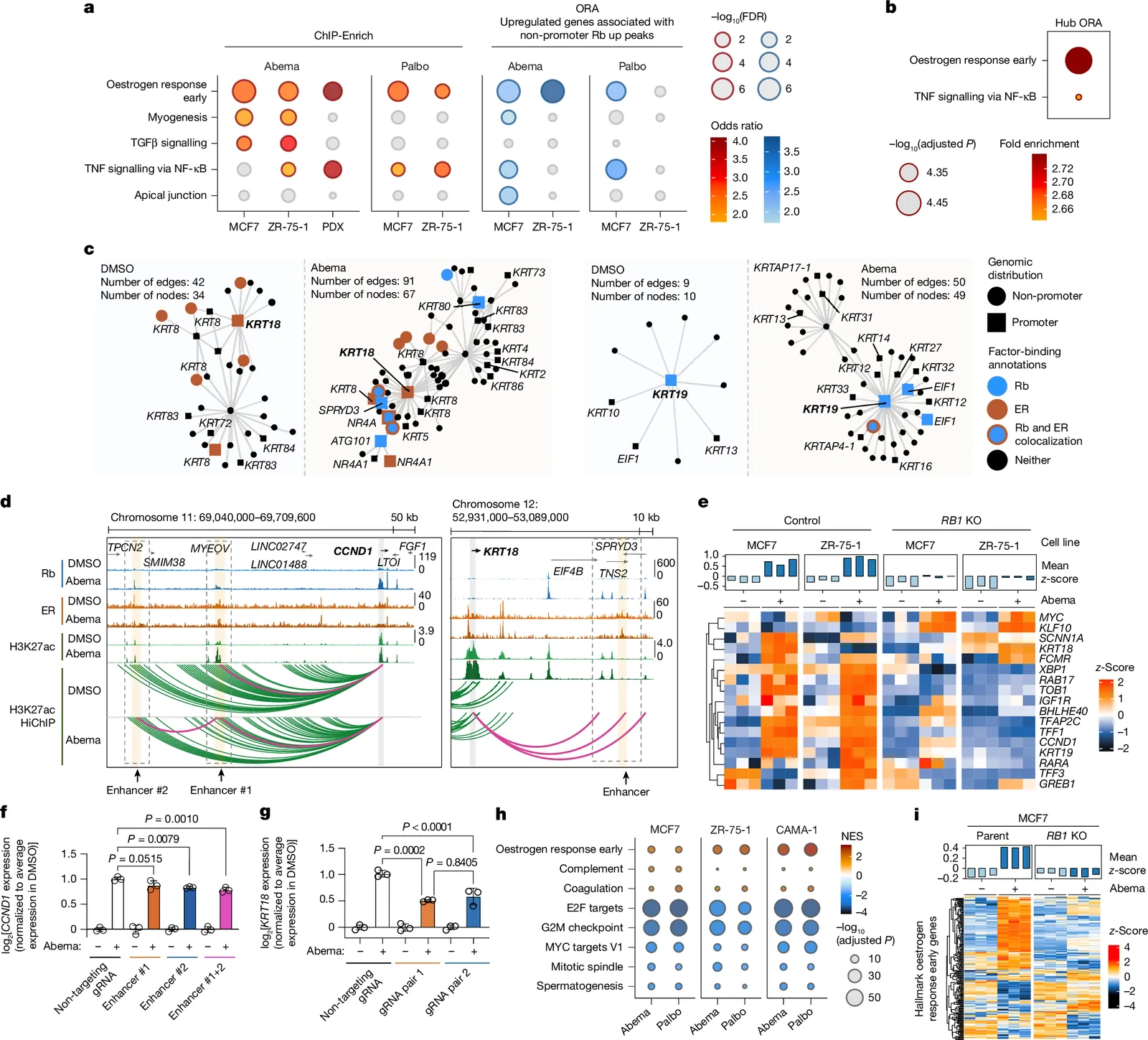
Rb engages enhancer networks to enhance the oestrogen-responsive programme. **a**,**b**, Gene set enrichment analyses (GSEAs) of genes associated with non-promoter Rb up peaks following CDK4/6 inhibition (**a**) and of genes whose promoters reside in H3K27ac HiChIP transcriptional hubs containing at least one non-promoter Rb peak in MCF7 cells following abemaciclib treatment (**b**). In **a**, the grey circles denote gene sets with FDR > 0.05. In **b**, adjusted *P* values were calculated using a hypergeometric test with Benjamini–Hochberg correction. Gene sets with adjusted *P* < 0.05 are shown. Palbo, palbociclib; ORA, over-representation analysis. **c**, Representative 3D chromatin interaction hubs containing hallmark oestrogen response early genes. Grey lines indicate chromatin interactions detected by H3K27ac HiChIP. **d**, Representative genomic regions in which non-promoter Rb up peaks (yellow highlights) are linked to hallmark oestrogen response early genes through H3K27ac HiChIP interactions. The solid grey boxes indicate promoters of oestrogen-responsive genes. The interactions in magenta denote interactions that were gained or strengthened following treatment. Representative oestrogen-receptor target genes are shown in bold in **c** and **d**. Black arrows indicate the genomic regions targeted in **f** and **g**. **e**,**i**, Heatmaps showing the expression of hallmark oestrogen response early genes linked to non-promoter Rb up peaks through an H3K27ac HiChIP interaction (**e**) or the complete gene set (**i**). Columns represent biological replicates, and rows represent genes. The bar plots indicate the mean *z*-score for each sample. KO, knockout. **f**,**g**, Expression of *CCND1* (**f**) and *KRT18* (**g**) in MCF7 cells following CRISPR–Cas9 targeting the regions indicated in **d**. Data presented as mean ± s.d.; *n* = 3 independent biological cultures per group. One-way analysis of variance (ANOVA) with Tukey’s multiple comparisons test was used. gRNA, guide RNA. **h**, Hallmark GSEA of RNA-seq data following CDK4/6 inhibitor treatment. Only gene sets with Benjamini–Hochberg-adjusted *P* < 0.05 in at least one comparison are shown. Chromatin profiling and RNA-seq experiments were performed using *n* = 2 and *n* = 3 independent biological replicates per condition, respectively. Values shown as *P* < 0.0001 correspond to the lower reporting limit of GraphPad Prism. NES, normalized enrichment score. Source data

We then returned to our Rb epigenetic profiling data and used ChIP-Enrich^24^ to examine the functional categories of genes that were linked to non-promoter Rb up peaks induced by CDK4/6 inhibition. This analysis revealed significant enrichment for oestrogen response genes—including canonical ER targets involved in luminal identity and proliferation—both in vitro and in vivo (Fig. 2a, Extended Data Fig. 3g,h and Supplementary Tables 2 and 3). Although this analysis was based on non-promoter peaks, closer inspection of individual genes revealed that Rb binding at ER target genes occurred at distal enhancers, at promoter-proximal regions and within broader regulatory domains marked by H3K27ac and H3K4me3 (trimethylation of lysine 4 on histone H3) (Extended Data Fig. 3h). Of note, although some of these genes (such as *CCND1*) contribute to cell cycle progression, they are not classical E2F-regulated targets and thus fall outside the canonical Rb–E2F axis.

Because promoter regions include well-characterized Rb–E2F interactions that could obscure novel biology, we continued to focus our ontological analysis on non-promoter Rb peaks to better understand the association between chromatin-bound Rb and oestrogen-response genes. When we restricted our analysis to non-promoter Rb up peaks linked to significantly upregulated genes, over-representation analysis also revealed significant enrichment for oestrogen response pathways (Fig. 2a, Extended Data Fig. 3g and Supplementary Table 4). Of note, these peaks showed minimal Rb occupancy before treatment, consistent with recruitment of hypophosphorylated Rb to chromatin upon CDK4/6 inhibition (Extended Data Fig. 3i). Transcriptional hubs containing non-promoter Rb up peaks were also frequently enriched for ER target genes, and 188 of 345 (54%) Rb-associated hubs also included ER-bound nodes, as defined by CUT&RUN (Fig. 2b,c and Extended Data Fig. 3g). This suggests spatial colocalization of Rb and ER within active chromatin domains.

## Rb promotes oestrogen-responsive transcription

**Extended Data Fig. 4:**
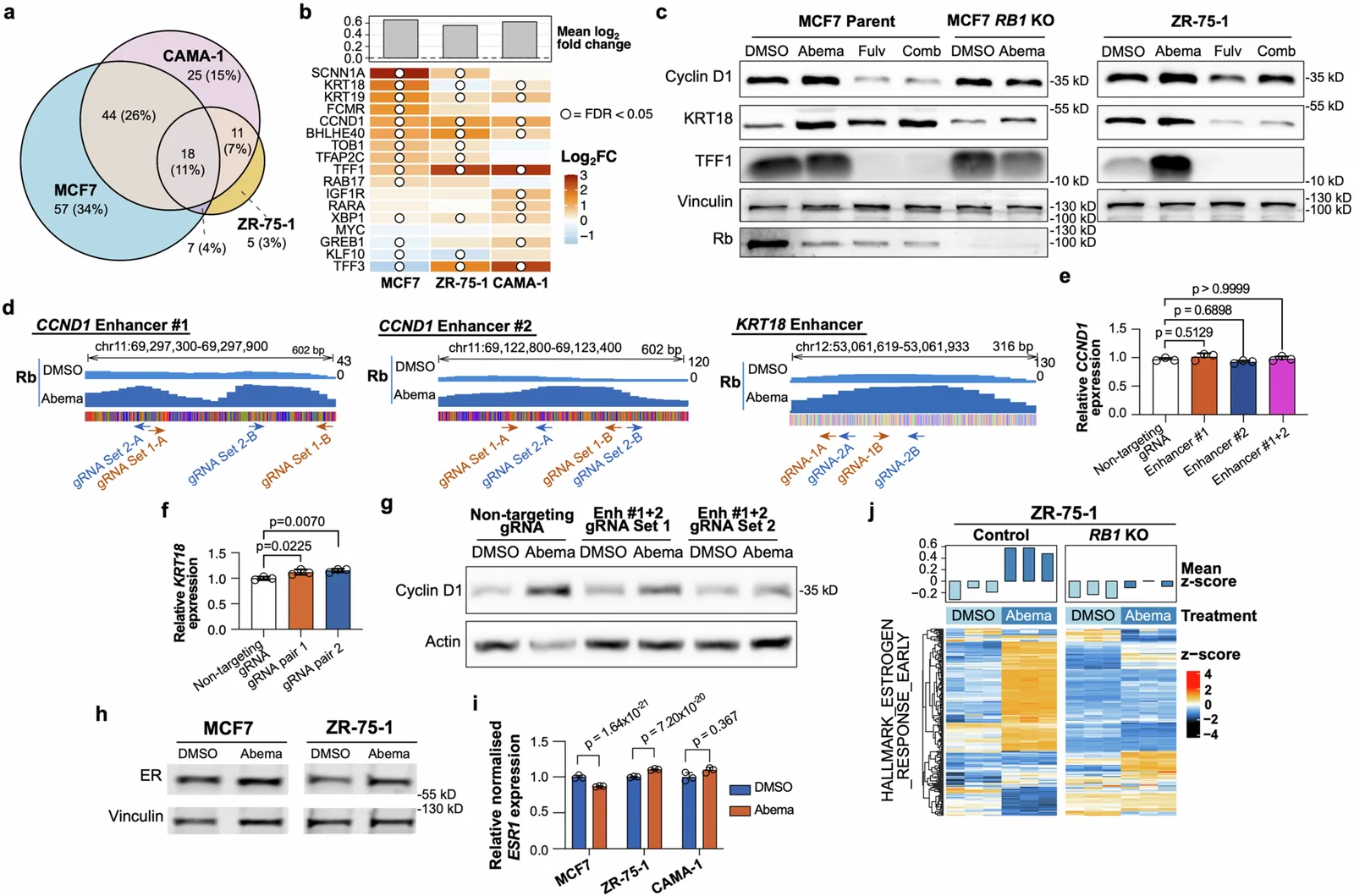
CDK4/6 inhibition-induced Rb binding at enhancers upregulates ER targets. **a**, Overlap of abemaciclib-upregulated genes from linked to non-promoter Rb up peaks by H3K27ac HiChIP loops in MCF7 cells. **b**, Heatmap showing expression of 17 *Hallmark Estrogen Response Early* genes linked to non-promoter Rb up peaks by H3K27ac HiChIP interactions. Open circles, adjusted P < 0.05 from DESeq2 (two-sided Wald test with Benjamini-Hochberg correction for multiple testing). Bar plots, mean log_2_ fold change in each cell line. **c, h**, Immunoblot analysis of ER target proteins (c) and ER (h) following the indicated treatments for 5 days. **d**, Genomic locations of Rb up peaks that contact the *CCND1* or *KRT18* promoters as indicated in Fig. 2d and the corresponding sgRNA target sites used for CRISPR-Cas9-mediated disruption. **e, f**, Basal expression of *CCND1* (e) and *KRT18* (f) in MCF7 cells treated either a non-targeting sgRNA or sgRNAs targeting the enhancer regions shown in **d**, measured by RT–qPCR. Data represent mean ± s.d. from *n* = 3 independent cultures per condition. *P* values were calculated using two-sided one-way ANOVA with Bonferroni correction. **g**, Immunoblot analysis of cyclin D1 in MCF7 cells following CRISPR-Cas9-mediated disruption of the genomic regions indicated in Fig. 2d using two independent sgRNA pairs illustrated in **d**. **i**, Relative *ESR1* expression levels from RNA-seq in ER-positive breast cancer cell lines treated as indicated for 5 days. Adjusted *P* values are from DESeq2 as in **b**. Bars indicate the mean; error bars denote s.d. **j**, Heatmap showing expression of *Hallmark Estrogen Response Early* genes in ZR-75-1 cells treated as indicated for 5 days. Rows represent individual genes and columns represent biological replicates. Bar plots indicate mean z-score in each sample. All RNA-seq were performed on *n* = 3 biologically independent samples per condition. For gel source data, see Supplementary Fig. 1. *RB1* KO, isogenic *RB1* knockout cells; Fulv, fulvestrant; Abema, abemaciclib; gRNA, guide RNA. Source data

To assess whether these Rb-bound loci physically interact with oestrogen-responsive gene promoters, we interrogated chromatin loops in our HiChIP data. We identified 405 non-promoter Rb-bound elements that looped to gene promoters in MCF7 cells. These elements also showed increased Rb binding in ZR-75-1 cells and PDX tumour tissue, and the gene promoters looping to them were not only strongly associated with gene upregulation but again also significantly enriched for oestrogen response (Extended Data Figs. 3g,j,k and 4a and Supplementary Table 5). Among them were 17 genes from the hallmark oestrogen response early gene set^25,26^—including canonical targets such as *CCND1* and *TFF1*, as well as luminal-associated genes such as *KRT18* and *KRT19*—most of which were significantly upregulated following abemaciclib treatment at the mRNA and protein levels (Fig. 2d,e and Extended Data Fig. 4b,c).

To test whether Rb contributes functionally to this transcriptional response, we used CRISPR–Cas9 to disrupt Rb-bound enhancers looping to the *CCND1* and *KRT18* promoters (Extended Data Fig. 4d). Critically, although mutation of these enhancers did not reduce basal *CCND1* or *KRT18* expression, it significantly impaired induction of these genes upon abemaciclib treatment (Fig. 2f,g and Extended Data Fig. 4e,f). Disruption of the *CCND1* enhancer also reduced cyclin D1 protein levels, confirming the functional impact of this regulatory element (Extended Data Fig. 4g). These findings support a model in which CDK4/6 inhibition recruits hypophosphorylated Rb to regulatory elements within ER-enriched chromatin hubs, enabling transcriptional activation of oestrogen-responsive genes.

To assess the broader relevance of this phenomenon, we analysed RNA sequencing (RNA-seq) data from three ER-positive breast cancer cell lines (MCF7, ZR-75-1 and CAMA-1) treated with abemaciclib or palbociclib. Oestrogen response gene sets were consistently upregulated across all models, despite there being no major change in ER expression levels (Fig. 2h and Extended Data Fig. 4h,i). Of note, CRISPR–Cas9-mediated *RB1* knockout abolished this transcriptional response (Fig. 2i and Extended Data Fig. 4j). The same effect of *RB1* knockout was observed when we specifically examined the 17 ER target genes whose promoters were directly connected via H3K27ac-marked loops to non-promoter Rb-bound regions, supporting the concept that Rb is functionally required for upregulation of these genes after CDK4/6 inhibition (Fig. 2e).

## Rb shapes responses to endocrine therapy

**Fig. 3:**
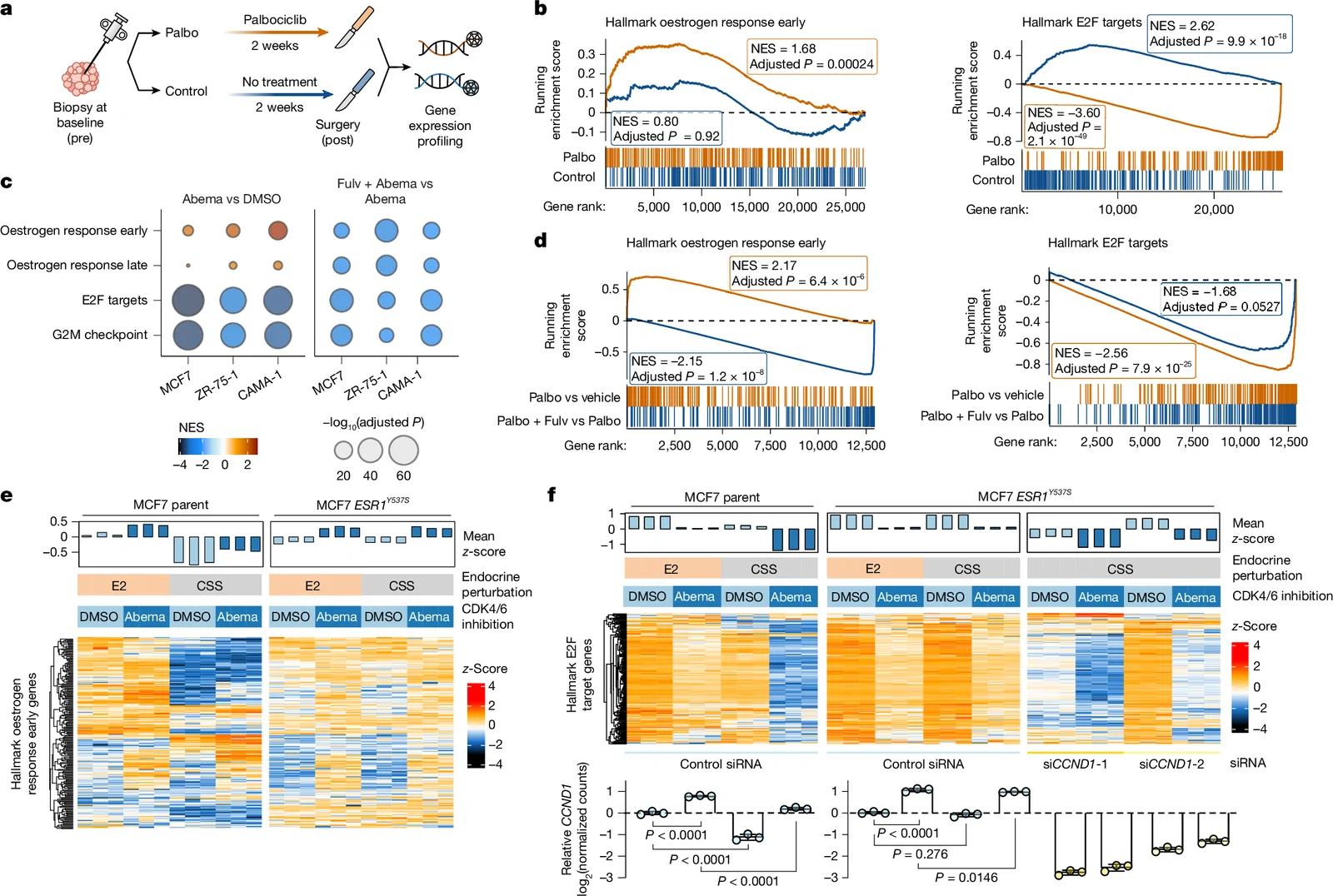
Rb-mediated ER activation shapes endocrine responses. **a**, Schematic of the POP clinical trial. Patients with primary ER^+^ breast cancer were assigned to no treatment control or palbociclib treatment, and tumour specimens were collected before treatment and after 2 weeks. Transcriptomic profiling was performed on paired tumour samples (*n* = 18 pairs for control; *n* = 44 pairs for palbociclib). **b**, GSEA enrichment and barcode plots in paired POP clinical trial samples at surgery compared with baseline (post versus pre) described in **a**. **c**,**d**, GSEA for oestrogen response-related and cell cycle-related hallmark gene sets following CDK4/6 inhibition and fulvestrant (Fulv) treatment in cell lines (**c**) and PDX tumours (**d**). In **c**, RNA-seq was performed following 5 days of treatment. In **d**, RNA-seq was performed following 19 days of treatment. **e**,**f**, Heatmaps showing the expression of hallmark oestrogen response early genes (**e**) and hallmark E2F target genes (**f**) under the indicated conditions. The rows represent genes, the columns represent biological replicates, and the bar plots indicate the mean *z*-score for each sample. In **e**, cells were cultured in charcoal-stripped serum (CSS) or stimulated with oestradiol (E2) and treated with DMSO or abemaciclib. In **f**, cells were additionally transfected with non-targeting control siRNA or siRNA targeting *CCND1* (si*CCND1*). Normalized *CCND1* expression from the same RNA-seq dataset is also shown (bottom). RNA-seq in cell lines was performed using *n* = 3 independent biological cultures per condition, and *n* = 2 tumours per condition for the PDX model. Data are presented as mean ± s.d. *P* values were calculated using one-way ANOVA followed by Bonferroni correction (two tailed). Values shown as *P* < 0.0001 correspond to the lower reporting limit of GraphPad Prism and do not represent the exact *P* value. Source data

**Extended Data Fig. 5:**
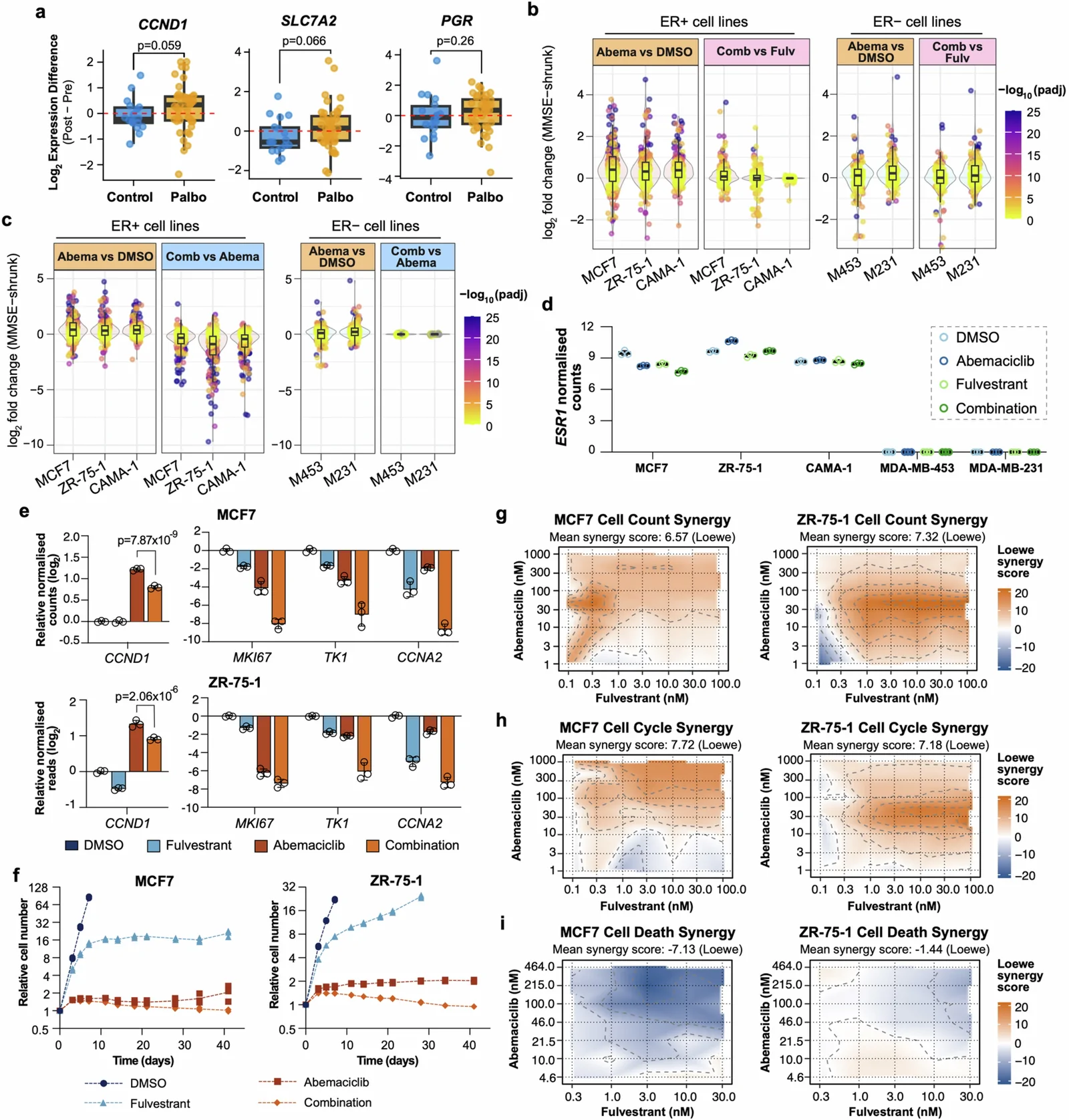
Impact of combined CDK4/6 inhibition and endocrine therapy by ER status. **a**, Expression of oestrogen-responsive genes in the POP clinical trial transcriptomic dataset. Data depicted as expression differences between post- and pre-treatment samples in control and palbociclib arms. Differential expression was assessed using limma empirical Bayes moderated t-tests with Benjamini-Hochberg correction for multiple testing; adjusted *P* values are shown (palbociclib *n* = 44 pairs; control *n* = 18 pairs). **b, c**, Distribution of log_2_ fold changes for genes in the *Hallmark Estrogen Response Early* gene set in the indicated comparisons. Each point represents one gene and is coloured according to its adjusted *P* value from DESeq2 (two-sided Wald test with Benjamini-Hochberg correction for multiple testing). **d**, Normalised RNA-seq counts of the *ESR1* gene in breast cancer cell lines treated as indicated for 5 days. Centre values indicate the mean; error bars denote s.d. **e**, RNA-seq expression of *CCND1* and E2F target genes relative to DMSO in cells treated as indicated for 5 days. Adjusted *P* values from DESeq2 (two-sided Wald test with Benjamini-Hochberg correction for multiple testing). Data represent mean ± s.d. **f**, Growth curves of H2B-GFP-expressing cells. Cell numbers were quantified by longitudinal live-cell imaging over 40 days. Each data point represents one replicate; *n* = 4 independent cultures per group. **g-i**, Two-dimensional contour plots visualising the Loewe synergy scores for abemaciclib and fulvestrant combinations following 5 days of treatment. Synergy was assessed using cell number (**g**), EdU incorporation (**h**), or propidium iodide staining (**i**). Positive scores indicate synergy and negative scores indicate antagonism. *n* = 3 technical replicates; representative of two biological experiments. Unless otherwise noted, all RNA-seq experiments were performed on *n* = 3 biologically independent samples per condition. Boxplots show the median (centre line), interquartile range (box), and 1.5×IQR (whiskers). Palbo, palbociclib; Abema, abemaciclib; ER+, ER-positive; ER-, ER-negative; Fulv, fulvestrant; Comb or combination, combined abemaciclib and fulvestrant treatment. Source data

To assess whether Rb activation enhances oestrogen-responsive gene expression in patients with luminal breast cancer, we analysed paired tumour samples from patients enrolled in the POP trial (NCT02008734). Participants in the POP trial received 2 weeks of neoadjuvant palbociclib monotherapy or no therapy (control) before surgery (Fig. 3a). Palbociclib has previously been reported to reduce Rb phosphorylation in this cohort^27^. Gene expression profiling of these tumours revealed significant suppression of E2F targets and induction of oestrogen response gene sets following treatment in the palbociclib group (*n* = 44 paired samples), but not in the control group (*n* = 18 paired samples; Fig. 3b and Extended Data Fig. 5a).

Although CDK4/6 inhibitor monotherapy has limited clinical efficacy^28^, combining these agents with endocrine therapies (anti-oestrogens) is highly effective^9^. Given the established association between elevated oestrogen response gene expression and endocrine sensitivity^29,30^, our results imply that Rb activation facilitates transcriptional induction of ER target genes, thereby reprogramming cells towards increased endocrine sensitivity.

**Extended Data Fig. 6:**
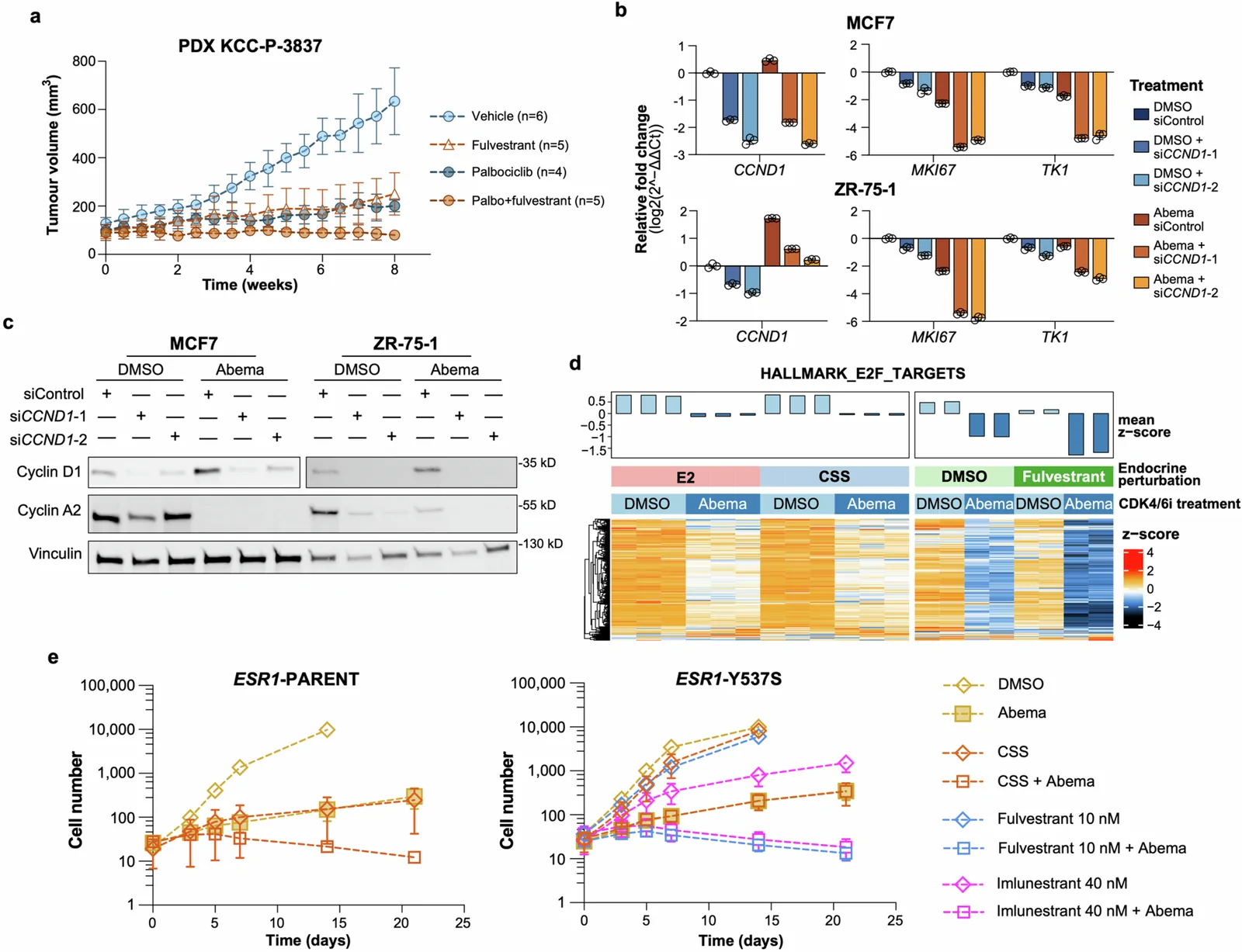
Concomitant CDK4/6 inhibition and endocrine therapy across *ESR1* genotypes. **a**, Tumour volume curves of KCC-P-3837 breast cancer PDX treated as indicated and monitored for 8 weeks. Tumour numbers for each group are indicated. Data represent mean ± s.d. **b**, Expression of *CCND1* and E2F target genes following *CCND1* or control siRNA transfection and the indicated treatments for 5 days, relative to DMSO-treated control siRNA cells, measured by RT-qPCR. Data represent mean ± s.d. from *n* = 3 biologically independent samples per group. **c**, Protein levels of cyclin D1 and cyclin A2 in cells treated as in **b**. For gel source data, see Supplementary Fig. 1. **d**, Heatmap showing expression of *Hallmark E2F Targets* genes in MCF7 *ESR1*-Y537S cells under indicated conditions. Left, cells cultured in charcoal stripped serum (CSS) medium and treated with abemaciclib with or without oestradiol (E2) stimulation (*n* = 3 independent cultures per group). Right, cells cultured in complete medium and treated with abemaciclib and fulvestrant for 5 days (*n* = 2 independent cultures per group). Rows represent individual genes and columns represent biological replicates. Bar plots indicate mean z-score in each sample. **e**, Growth curves of H2B-GFP-expressing MCF7 cells cultured under complete medium and treated with DMSO, abemaciclib, fulvestrant, or imlunestrant, or in CSS and treated with DMSO or abemaciclib. Cell number was quantified by longitudinal live-cell imaging over 21 days and the data represent mean ± s.d. from *n* = 4 independent cultures per condition. PDX, patient-derived xenograft; Abema, abemaciclib. Source data

To investigate the impact of inhibiting this Rb-induced oestrogenic transcriptional programme, we treated HR^+^ breast cancer cell lines (MCF7, ZR-75-1 and CAMA-1) with abemaciclib, the selective ER degrader fulvestrant or their combination. Consistent with our earlier findings, CDK4/6 inhibition alone elevated oestrogen response gene expression in all cell lines, an effect that was markedly attenuated by fulvestrant co-treatment and confirmed at the protein level for representative ER targets (Fig. 3c and Extended Data Figs. 4c and 5b,c). The importance of ER signalling in this response was further supported by the markedly attenuated induction of hallmark oestrogen response early genes in *RB1* wild-type, ER-negative cell lines treated with abemaciclib (Extended Data Fig. 5b–d). Of note, addition of fulvestrant enhanced the suppression of E2F target genes compared with abemaciclib alone in ER-positive models, correlating with more robust inhibition of tumour cell proliferation and sustained suppression of cellular outgrowth, without increasing cell death (Fig. 3c and Extended Data Fig. 5e–i). Similar observations were obtained in the PDX model, in which palbociclib monotherapy again induced oestrogen-responsive genes, and combination treatment yielded superior suppression of E2F targets and tumour growth (Fig. 3d and Extended Data Fig. 6a). Thus, ER-targeted therapies effectively blunt the Rb-mediated transcriptional feedback on ER target genes, leading to deeper cell cycle arrest.

As noted earlier, *CCND1*, which encodes cyclin D1 (a known mediator of resistance to CDK4/6 inhibitors^31–34^), was among the oestrogen-responsive genes induced by Rb binding to nearby distal regulatory elements. Consistent with this, fulvestrant-mediated attenuation of CDK4/6 inhibitor-induced upregulation of *CCND1* was associated with more robust suppression of E2F target genes (Extended Data Fig. 5e). Knockdown of *CCND1* in CDK4/6 inhibitor-treated cells led to a deeper suppression of E2F target genes (Extended Data Fig. 6b), implicating cyclin D1 as a key component of this Rb-induced compensatory proliferative response. This effect was also evident at the protein level, with reduced expression of the representative E2F target cyclin A2 (Extended Data Fig. 6c). These findings illustrate that Rb activation, although initially anti-proliferative, can paradoxically engage transcriptional networks that support proliferative capacity over time.

## Rb activates ER targets in *ESR1* mutants

To examine how these feedback mechanisms operate in the context of endocrine resistance, we examined *ESR1*-mutant models, which display ligand-independent ER activity and are resistant to aromatase inhibitors^35^. Introduction of the *ESR1*^*Y537S*^ mutation into the endogenous locus of MCF7 cells^35^ revealed that CDK4/6 inhibition induced oestrogen response genes—including *CCND1*—and this induction persisted under oestrogen-depleted conditions that mimic aromatase inhibitor therapy (Fig. 3e). This sustained activation of ER targets was associated with attenuated suppression of E2F target genes upon concomitant CDK4/6 inhibition and oestrogen deprivation, potentially facilitating cell cycle escape and resistance (Fig. 3f). Switching from oestrogen deprivation to a selective oestrogen receptor degrader (SERD), which can degrade mutant ER, restored suppression of E2F targets and control of tumour cell proliferation (Extended Data Fig. 6d,e). Of note, among the ER-responsive genes induced, knockdown of *CCND1* alone was sufficient to drive deeper suppression of E2F target genes in *ESR1*-mutant cells treated with abemaciclib (Fig. 3f).

These findings indicate that constitutive ER activity in *ESR1*-mutant cells can limit complete cell cycle repression during combined CDK4/6 inhibition and oestrogen deprivation. Pharmacological suppression of ER signalling with a selective oestrogen receptor degrader restores this repression, providing a mechanistic framework consistent with clinical observations and suggesting that early SERD-based therapy may help to delay the emergence of overt clinical resistance^36,37^.

## Rb relieves KDM5A-mediated repression

**Fig. 4:**
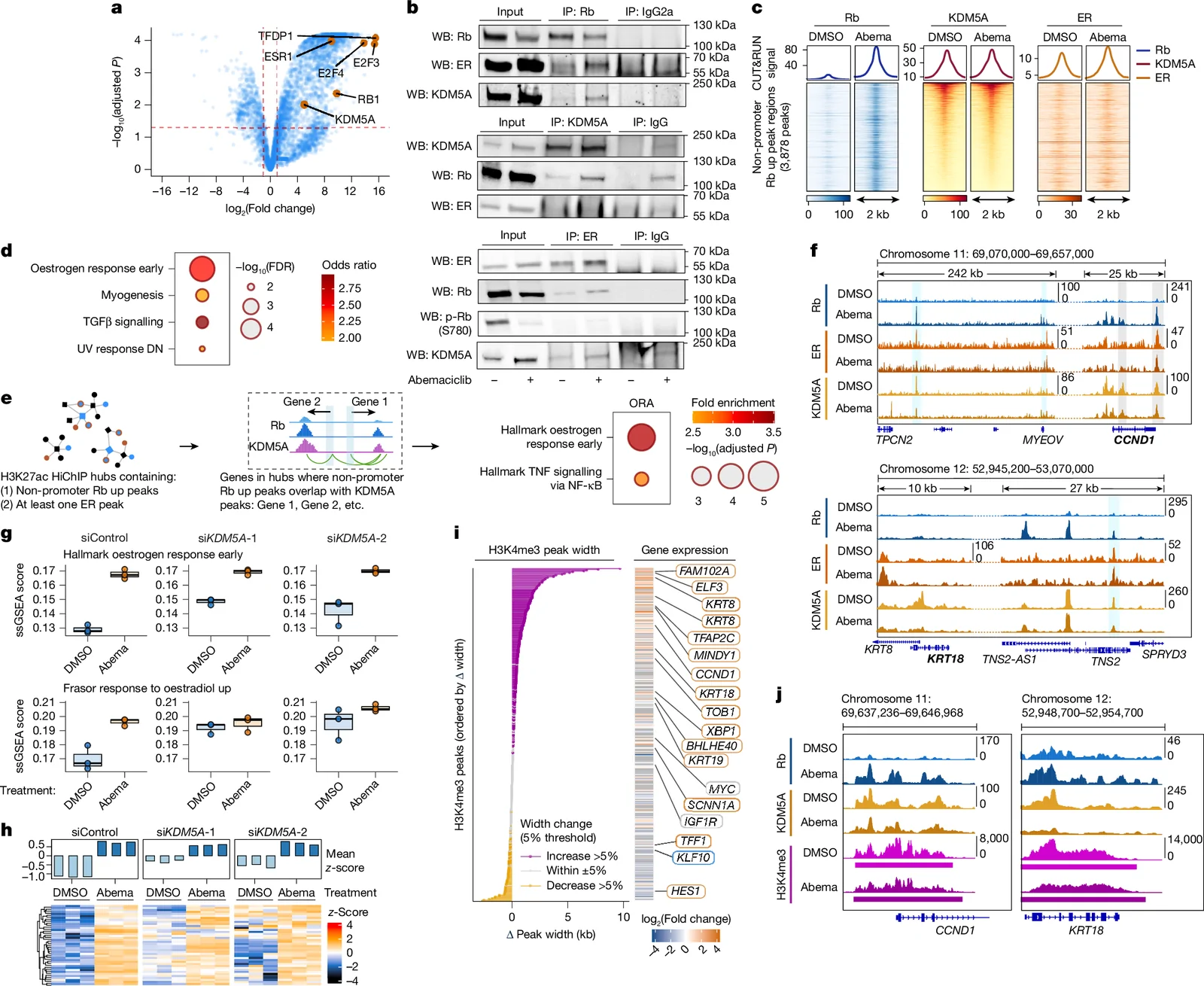
Rb derepresses the oestrogen response programme through interaction with KDM5A. Unless otherwise indicated, experiments were performed in MCF7 cells. **a**, Volcano plot of proteins enriched in Rb immunoprecipitates relative to IgG controls in nuclear lysates following abemaciclib treatment, identified by immunoprecipitation (IP)–mass spectrometry. Differential enrichment was assessed using limma’s empirical Bayes-moderated *t*-tests with Benjamini–Hochberg correction. For Rb immunoprecipitation, *n* = 3, and for IgG immunoprecipitation, *n* = 2 independent biological replicates. **b**, Immunoblot analysis of Rb, KDM5A and ER immunoprecipitates from nuclear lysates. Rb and ER immunoprecipitates are representative of two independent experiments. For gel source data, see Supplementary Fig. 1. WB, western blot. **c**, Composite profiles and heatmaps of chromatin occupancy at non-promoter Rb up peaks from Fig. 1b. The rows represent genomic regions, and the colours indicate signal intensity. For Rb and KDM5A, one representative of two biological replicates is shown. **d**,**e**, Gene set enrichment of non-promoter Rb up peaks overlapping KDM5A-binding sites using ChIP-Enrich (**d**) and genes residing within transcriptional hubs containing both non-promoter Rb up peaks overlapping KDM5A and at least one ER peak (**e**). In **e**, adjusted *P* values were calculated using a hypergeometric test with Benjamini–Hochberg correction. DN, down. **f**,**j**, Representative chromatin occupancy profiles at oestrogen-responsive genes (bold). In **f**, highlighted regions indicate non-promoter Rb up peaks associated with nearby genes (grey) or linked to promoters through H3K27ac HiChIP interactions (light blue). In **j**, the purple bars indicate H3K4me3 peak width. **g**,**h**, RNA-seq single-sample GSEA (ssGSEA) scores for oestrogen-responsive gene sets (**g**) and expression of hallmark oestrogen response early genes upregulated by abemaciclib in siControl cells (**h**) following *KDM5A* knockdown. In **g**, the points represent individual biological replicates; the boxplots show the median (centre line), interquartile range (boxes) and ±1.5× the interquartile range (whiskers). *P* values were calculated by one-way ANOVA with Bonferroni correction. In **h**, the rows represent genes and the columns represent biological replicates; the bar plots indicate mean *z*-scores for each sample. **i**, Changes in promoter-associated H3K4me3 peak width at oestrogen-responsive genes identified in **e**; each row corresponds to one peak (left). A corresponding expression heatmap of linked genes (enriched genes are labelled: orange denotes upregulated, blue shows downregulated, and grey indicates non-differentially expressed) is also shown (right). For Rb, KDM5A and H3K4me3 CUT&RUN, *n* = 2 independent cultures per condition; for ER CUT&RUN, *n* = 1 independent culture per condition. For RNA-seq, *n* = 3 biologically independent samples per condition. Source data

**Extended Data Fig. 7:**
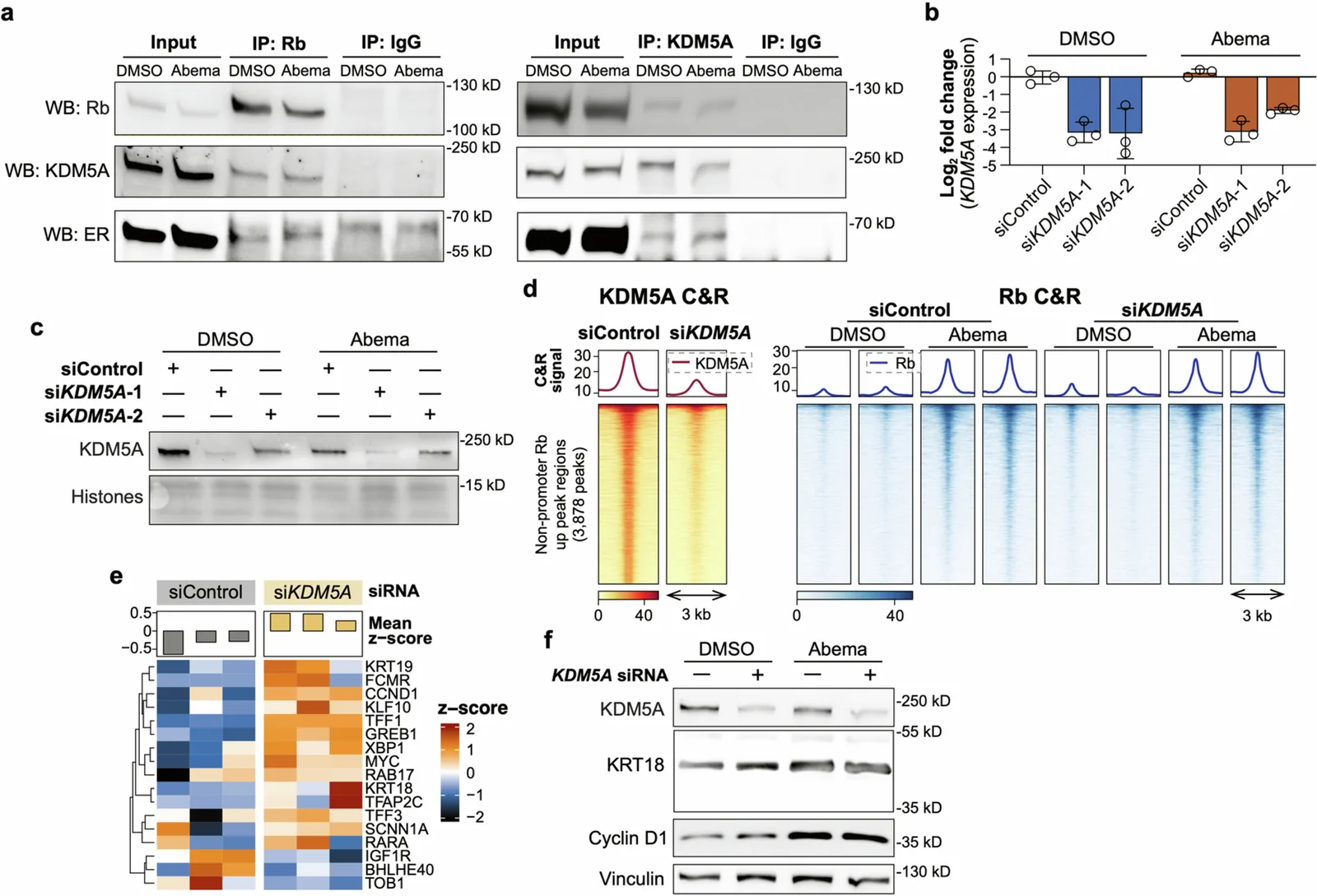
Effects of KDM5A knockdown on Rb and oestrogen-responsive genes. **a**, Immunoblot analysis of Rb and KDM5A immunoprecipitates from ZR-75-1 nuclear lysates. Experiment was not independently repeated. **b, c**, KDM5A expression measured by RNA-seq (b) and immunoblot analysis (c) in MCF7 cells transfected with control or *KDM5A* siRNA and treated as indicated. RNA-seq data represent mean ± s.d. from *n* = 3 independent cultures per condition. **d**, Composite profiles and heatmaps of KDM5A CUT&RUN signal following control or *KDM5A* siRNA transfection (left) and Rb CUT&RUN signal under indicated conditions (right), at non-promoter Rb up peak regions in Fig. 1b,c. Each row represents a genomic region; colour indicates signal intensity. KDM5A CUT&RUN, *n* = 1 reaction per condition; Rb CUT&RUN, *n* = 2 reactions per condition. **e**, Heatmap showing expression of the 17 ER target genes shown in Fig. 2e in MCF7 cells treated with control or KDM5A siRNA (si*KDM5A*-1). **f**, Immunoblot analysis of MCF7 cells transfected with control or KDM5A siRNA under indicated treatments for 3 days. KDM5A siRNA efficacy repeated 3 independent times. Cyclin D1 and KRT18 examined only in the representative experiment shown. For gel source data, see Supplementary Fig. 1. IP, immunoprecipitation; WB, western blot; Abema, abemaciclib; C&R, CUT&RUN. Source data

We next sought to identify additional chromatin-associated proteins that cooperate with hypophosphorylated Rb to promote transcriptional activation of oestrogen-responsive genes. Given the established capacity of Rb to bind to chromatin-modifying enzymes, we profiled its nuclear interactome. Immunoprecipitation for Rb followed by mass spectrometry was performed using nuclear extracts from abemaciclib-treated MCF7 cells. As expected, canonical Rb interactors were enriched, validating the specificity of the approach (Fig. 4a and Supplementary Table 6). Of note, we identified an interaction between Rb and ER, to our knowledge a previously undescribed association in this context, consistent with our observation of their colocalization within transcriptional hubs (Fig. 4a). We also observed an interaction between Rb and the histone demethylase KDM5A, a known Rb-binding partner with context-dependent roles in gene regulation and differentiation, including ER-associated transcription^38,39^ (Fig. 4a). These interactions were independently confirmed by co-immunoprecipitation and western blotting in two cell lines (Fig. 4b and Extended Data Fig. 7a). Of note, three-way reciprocal co-immunoprecipitation in MCF7 cells revealed that pulldown of any one of Rb, ER and KDM5A co-precipitated the other two proteins, providing strong evidence for the formation of a shared nuclear complex (Fig. 4b). Although enhancement of these interactions following CDK4/6 inhibition was more pronounced in MCF7 cells than in ZR-75-1 cells, the complex was detectable in both models.

To better understand the chromatin context of these interactions, we examined sites of Rb chromatin binding that were gained upon CDK4/6 inhibition and located outside gene promoters. These loci showed marked overlap with KDM5A-binding and ER-binding sites, consistent with the formation of transcriptional hubs incorporating all three factors (Fig. 4c). Neither KDM5A nor ER exhibited substantial changes in occupancy at these sites following CDK4/6 inhibition, indicating that they are already present at these loci before Rb recruitment (Fig. 4c). Furthermore, *KDM5A* knockdown did not alter Rb recruitment to these loci (Fig. 4c and Extended Data Fig. 7b–d). Together, these findings support a model in which hypophosphorylated Rb is recruited to pre-established KDM5A-bound regions at ER target genes, rather than being directly recruited by KDM5A or directing the initial formation of these hubs.

Genes associated with these Rb–KDM5A–ER co-bound regions were significantly enriched for oestrogen response signatures, reinforcing the idea that they act to coordinate hormone-responsive transcription (Fig. 4d). Further supporting this idea, over-representation analysis of genes within transcriptional hubs displaying both ER binding and Rb recruitment to a KDM5A-bound region revealed a strong bias towards genes involved in oestrogen signalling (Fig. 4e). Of note, 57% of genes within these hubs that were also listed in the hallmark oestrogen response early gene set exhibited Rb–KDM5A co-binding not only at non-promoter regions but also at their promoters (examples in Fig. 4f). Together, these findings suggest that Rb and KDM5A participate in enhancer–promoter-looping interactions that potentiate oestrogen-driven gene expression in CDK4/6-inhibited cells.

To investigate how Rb recruitment to KDM5A-bound regions promotes gene activation, we first assessed the role of KDM5A in these cells. Short interfering RNA (siRNA)-mediated knockdown of *KDM5A* (Extended Data Fig. 7b,c) substantially upregulated oestrogen response genes, including the subset of 17 MCF7 genes linked to Rb-bound enhancer–promoter loops, and increased protein expression of representative ER targets (Fig. 4g,h and Extended Data Fig. 7e,f). These findings are consistent with previous reports suggesting that Rb binding inhibits the demethylase activity of KDM5A and thereby its repressive function^40^. The absence of additional induction following combined *KDM5A* knockdown and CDK4/6 inhibition indicates that Rb promotes transcription by relieving KDM5A-mediated repression (Fig. 4g,h).

To further explore this mechanism, we assessed the levels of H3K4me3—the substrate of KDM5A—at the promoters and gene bodies of Rb–KDM5A–ER hub-associated genes. CDK4/6 inhibition led to broad spreading of H3K4me3 peaks at key oestrogen response genes, consistent with reduced KDM5A activity, accompanied by increased transcriptional output^41^ (Fig. 4i). Representative Integrative Genomics Viewer tracks confirmed increased H3K4me3 signal at selected oestrogen-responsive gene loci (Fig. 4j), supporting a model in which Rb-mediated repression of KDM5A activity facilitates chromatin changes that promote transcription.

## Discussion

The classical model of Rb function centres on its role in enforcing cell cycle arrest through repression of E2F target genes: a mechanism that underpins the clinical rationale for CDK4/6 inhibition in HR^+^ breast cancer. Here we have identified a second function of active Rb in cancer. Following CDK4/6 inhibition, hypophosphorylated Rb promotes oestrogen receptor-responsive transcription through direct engagement with chromatin-associated regulatory networks rather than as a secondary consequence of cell cycle arrest. Many of the promoter and non-promoter regions involved are unoccupied by Rb at baseline, indicating that CDK4/6 inhibition unmasks a latent transcriptional activity of Rb not previously observed at scale. These findings expand on earlier suggestions of the functional versatility of Rb by defining a chromatin-resident, gene-activating role with therapeutic consequences.

This dual functionality reframes Rb as a dynamic, context-dependent regulator of transcription in cancer cells. By activating pro-proliferative ER target genes such as *CCND1* while repressing E2F targets, Rb can both enhance and limit the therapeutic effects of CDK4/6 inhibition. On the one hand, Rb-mediated upregulation of ER targets underpins the observed synergy between CDK4/6 inhibitors and endocrine therapies. On the other hand, in *ESR1*-mutant models, the Rb-driven transcriptional program persists despite oestrogen deprivation and can limit complete cell cycle repression during CDK4/6 inhibition. This effect is reversed by pharmacological ER degradation, providing a mechanistic framework for the superior activity of SERDs in this setting.

To define the molecular basis of this transcriptional response, we mapped the nuclear Rb interactome and identified interactions with ER and the histone demethylase KDM5A. Many ER target genes showing non-promoter Rb recruitment also exhibited promoter co-occupancy by Rb and KDM5A, suggesting coordinated regulation across distal and promoter-associated elements. CDK4/6 inhibition increased H3K4me3 deposition at ER-regulated genes, whereas KDM5A depletion phenocopied the transcriptional effects of Rb activation and abolished further induction by abemaciclib. Together, these findings indicate that pharmacologically activated Rb promotes ER target gene expression, at least in part, by antagonizing KDM5A-mediated repression. Although ER signalling is a major contributor to this response in luminal breast cancer cells, our data do not establish that ER recruits Rb to chromatin, and additional transcription factors or chromatin-associated regulators may also participate.

More broadly, our findings challenge the longstanding view of Rb as an exclusively repressive tumour suppressor. Pharmacological activation of Rb does more than restore cell cycle control; it also engages transcriptional programs that can influence therapeutic response. More generally, these results raise the possibility that other tumour suppressors may possess context-dependent regulatory functions that become apparent only when they are pharmacologically activated.

## Methods

### Cell culture

MCF7 (HTB-22), ZR-75-1 (CRL-1500), CAMA-1 (HTB-21), MDA-MB-453 (HTB-131), MDA-MB-231 (HTB-26), OVCAR-3 (HTB-161) and Kuramochi (JCRB0098) cells were obtained from the American Type Culture Collection. MCF7 *ESR1*^*Y537S*^ cells were provided by S. Chandarlaparty^35^. MCF7, ZR-75-1, MDA-MB-231 and CAMA-1 cells were cultured in RPMI-1640 medium (11875-093, Gibco) supplemented with 10% fetal bovine serum (FBS; SH30054.03, GE HyClone; SFBS-AU, Bovogen Biologicals), 1× GlutaMAX (35050061, Gibco) and 1% HEPES (15630080, Gibco). OVCAR-3 and Kuramochi cells were cultured in RPMI-1640, supplemented with 10% FBS and 1× GlutaMAX. MDA-MB-453 cells were cultured in DMEM supplemented with 10% FBS. All cells were maintained at 37 °C and 5% CO_2_ in a humidified incubator. Cells were maintained below 90% confluency and routinely tested to confirm lack of mycoplasma contamination via PCR testing. Cell lines were authenticated by STR fingerprinting.

### In vitro drug treatment studies

Abemaciclib methanesulfonate (HY-16297) and palbociclib isethionate (HY-A0065) were purchased from MedChemExpress. INX-315 was obtained from Incyclix Bio. Fulvestrant (S1191) and imlunestrant (E1301) were purchased from Selleck Chemicals. All drugs were diluted in dimethyl sulfoxide (DMSO) for in vitro studies. Unless otherwise noted, abemaciclib and palbociclib were used at 500 nM, INX-315 was used at 300 nM, fulvestrant was used at 5 nM and imlunestrant was used at 40 nM.

For cell cycle analysis by BrdU, MCF7, OVCAR-3 and Kuramochi cells were treated with DMSO or drug (abemaciclib, palbociclib or INX-315, respectively) for 2 days; ZR-75-1 cells were treated for 4 days (abemaciclib or palbociclib). For CUT&RUN, MCF7, OVCAR-3 and Kuramochi cells were treated for 2 days, and ZR-75-1 cells were treated for 4 days. For dose matrix drug combination assays, cells were treated with abemaciclib and fulvestrant in a dose matrix using the Tecan D300e Digital Dispenser, with a treatment length of 5 days.

For hormone-deprivation and stimulation experiments, cells were cultured in phenol-free RPMI-1640 (11835030, Gibco) supplemented with 1× GlutaMAX, 1% HEPES and 10% charcoal-stripped FBS. Oestradiol (E2758, Sigma) dissolved in ethanol was used at 1 nM.

### Cell cycle analysis by flow cytometry

Thirty minutes before end point, 10 μM BrdU (B5002, Sigma) was applied to the cells. Cells were stained using the Near-IR Fixable Viability Stain (L34976, Invitrogen) for 20 min at room temperature. Cells were fixed and permeabilized using the FoxP3/Transcription Factor Staining Buffer Set (00-5523-00, eBioscience). DNA was denatured using 2 N HCl + 0.5% (v/v) Triton X-100 for 30 min. The acid was neutralized using 0.1 mol l^−1^ Na_2_B_4_O_7_•10H_2_O (pH 8.5) followed by 0.5% BSA in PBS. Cells were then stained with BrdU antibody (clone 3D4, 560209, BioLegend; 1:20) for 1 h. Before acquisition on the BD A3 Symphony, DNA was stained using FxCycle Violet Stain (F10347, Invitrogen). A minimum of 10,000 live cell events were recorded for each sample and data were analysed using FlowJo (v10.8.1).

### Dose matrix drug combination assays

#### Cell counts

Cells were fixed by adding 16% paraformaldehyde (PFA; C004, ProSciTech) to the media to attain a concentration of 4% PFA and incubated at room temperature for 10–15 min. Washed cells were then incubated with the DAPI (D1306, Invitrogen)–Hoechst 33342 (H1399, Thermo Fisher) stain solution (0.19% Triton X-100, 2.5 μg ml^−1^ DAPI and 5 μg ml^−1^ Hoechst in 50 mM Tris pH 7.6). The stained nuclei were imaged using the Cellomics CX7 LED or LZR (Thermo Fisher), and the CX7 software was used for segmentation and nuclei counting. Nuclei counts were used for synergy analysis.

#### Cell cycle

EdU was added to the cells at 10 μM and incubated at 37 °C for 45 min. Of PFA, 16% was added to attain 3.7% final concentration to fix the cells. EdU was labelled using the Click-iT EdU Cell Proliferation Kit for Imaging (C10340, Thermo Fisher) as per the manufacturer’s protocol. Once the staining cocktail was removed, cells were washed once in PBS containing 3% BSA, and nuclei were stained with DAPI and Hoechst as above. The EdU signal and nuclei were imaged using the CX7 LZR. The per cell data were analysed using the CellProfiler (v3.0.0) software. The percentage of cells in S phase was used for synergy analysis.

#### Cell death

Media and PBS washes from each well were collected to capture all floating or dead cells. Adherent cells were trypsinized and combined with floating cells in the media. Cells were centrifuged at 400*g* for 4 min at 4 °C. All cells were washed with PBS twice. Cells were stained with 80 μl propidium iodide from the Annexin V/PI Kit (556547, BD) at 1:20 in 1× binding buffer on ice for 15 min as per the manufacturer’s protocol. Cells were then analysed on the LSR II flow cytometer using the BD FACSDiva software (v9.0). Percentages of live and dead cells were calculated using FlowJo (v10.5.3), and this information was used for synergy analyses.

### Synergy analysis

Loewe synergy scoring in the SynergyFinder R package (v3.14.0)^42^ and ggplot2 (v3.2.5) were used to analyse and visualize the data.

### Cell proliferation assays

Live cells constitutively expressing H2B–GFP were imaged using the Incucyte SX5 at multiple time points. Using the images, cells were segmented based on GFP expression to obtain cell counts.

### In vivo experiments

The KCC-P-3837 PDX model has been previously described^43^. NOD.Cg-*Prkdc*^*scid*^*Il2rg*^*tm1Wjl*^ (NSG) mice were used for all in vivo experiments. Mice were housed under specific pathogen-free conditions at 19–21 °C and 40–60% relative humidity with ad libitum access to food and water. Animals were maintained on a 14-h light–10-h dark cycle (lights on at 07:00 and off at 20:00) with 30-min dawn and dusk transitions. Red lighting was used after hours during the dark phase. No formal statistical methods were used to predetermine sample sizes. Sample sizes were selected based on previous experiences on the PDX model used in this study. Before treatment initiation, aged-matched mice were randomized into groups of equal average tumour volume. Downstream analyses of mouse tissue, including immunohistochemistry and image analysis, were performed in a blinded manner. Individuals conducting the assay and/or analysing the data were unaware of treatment allocation until data gathering was complete.

#### Rb chromatin profiling and immunohistochemistry

Tumour pieces of 1–2 mm^3^ were implanted into the thoracic mammary fat pads of 7-week-old NGS female mice (obtained from the Peter MacCallum Cancer Centre Animal Facility) implanted with 0.3 mg oestradiol silicon pellets. When tumours reached an average of 250 mm^3^ (using the formula width^2^ × length × 0.5), mice were randomized into two treatment groups: abemaciclib or vehicle control. Abemaciclib and vehicle were prepared as previously described^44^. Mice were treated with 90 mg kg^−1^ abemaciclib by daily oral gavage, five doses in total, and then euthanized by CO_2_ asphyxiation or cervical dislocation 2–4 h after the last dose. This study was performed in compliance with federal laws and institutional guidelines as approved by the Animal Ethics Experimentation Committee of the Peter MacCallum Cancer Centre. Humane end points included any diameter of any single tumour measuring at greater than or equal to 15 mm, weight loss of more than or equal to 20%, any tumour ulceration or infection, loss of ability to ambulate, laboured respiration, impaired movement or ruffled fur. No animals exceeded the approved humane end point criteria during the course of this study.

#### RNA-seq and tumour growth

The KCC-P-3837 has been previously described^43^. At surgery, 4-mm^3^ sections of tumour tissue were implanted into the left and right fourth inguinal mammary glands of 6–8-week-old female NSG mice (Australian BioResources). Tumour growth was assessed by calliper measurement and mice were randomized to treatment arms when tumours reached 200–250 mm^3^. Mice were treated with palbociclib (100 mg kg^−1^ in water 5 days per week by oral gavage), fulvestrant (5 mg per mouse in 100 μl peanut oil once weekly by subcutaneous injections), the combination of palbociclib and fulvestrant, or vehicles only. One cohort of mice was treated for 56 days to produce tumour growth kinetics, whereas a second cohort was treated for 19 days and then euthanized by CO_2_ exposure and cervical dislocation. Tumours were resected, processed and snap frozen immediately following euthanasia. Experiments, procedures and end points were approved by the Garvan Institute of Medical Research Animal Ethics Committee. In brief, mice were monitored regularly for tumour burden and clinical signs of distress and were euthanized upon reaching predefined humane end points, including tumour ulceration, necrosis, infection, evidence of local invasiveness, impaired mobility, inability to access food or water, development of ascites, 20% or more body weight loss, or other indicators of compromised welfare. Animals were euthanized when tumour volume reached or exceeded 1,500 mm^3^. As euthanasia was initiated following tumour measurement, the final recorded tumour volume occasionally exceeded this threshold at the terminal assessment.

### CRISPR–Cas9 gene editing

Alt-R S.p. Cas9 nuclease-purified Cas9 protein (1081059, Integrated DNA Technologies (IDT)) and single guide RNAs (sgRNAs; custom ordered from IDT) were complexed at a 1:5 molar ratio to form ribonucleoproteins (RNPs). For each nucleofection reaction, 36.6 pmol of Cas9 and 150 pmol sgRNA were used. The RNP complexes were incubated at room temperature for 10 min. For each reaction, cells were suspended in SF Cell Line Nucleofector solution (V4XC-2032, Lonza) containing Cas9 protein and gRNA complexes. The Lonza 4D-Nucleofector X Unit and the MDA-MB-453 program were used for nucleofection.

sgRNA sequences were as follows. For *RB1*, sg*RB1*-2: 5′-AAA CAA TCA AAG GAC CGA GA; for *KRT18* enhancer, gRNA-1A: 5′-CAG GGC TCT AGA GTT CAC AG; gRNA-1B: 5′-AGC TTG GCA GCA GAG GAG GG; gRNA-2A: 5′-GTG GTG GCG GTA AGA GTC TG; gRNA-2B: 5′-GTG GGG AGG AGT TTT CAC AG; for *CCND1* enhancer #1, set 1A: 5′-ACACAAGACACGCTGCACGG; set 1B: 5′-AGGAAGCTTGCTGAACACCG; set 2A: 5′-GAGGTTACCCCTCATAATGG; set 2B: 5′-GGGGTCAAGAGGAAGCTTAG; for *CCND1* enhancer #2, set 1A: 5′-GAGCTGACTCGATTTGCCCG; set 1B: 5′-GCAGCACTCTGTACCCAGAA; set 2A: 5′-TGTTTCTGAGATCACAGGCG; set 2B: 5′-TCAGTGGTTTGCAGAGACGT; and for non-targeting control sgRNA, sgControl: 5′-CAT TTC TCA GTG CTA TAG AG.

### Generation of isogenic *RB1*-knockout cell lines

Viable bulk populations of edited cells defined by negative staining for propidium iodide at 1 μg ml^−1^ were single cell sorted by the BDFACS Aria Fusion 3 or 5 Flow Cytometer into each well of a 96-well plate. PCR amplification from genomic DNA was performed using the following primers: forward 5′-CTG TCC CTT GAA TGT TTG GTA G, reverse 5′-TCT GGA GAG GAA GAT TAA GAG GAC; and PCR amplicons were sequenced by Sanger sequencing. Cell lysates were analysed for *RB1* knockout by western blotting. Verified *RB1*-knockout clones were expanded and cryopreserved for further studies.

### Generation of nuclear GFP-expressing cells

Cell lines expressing H2B–GFP were generated using the lentiviral plasmid LV-GFP (Addgene #25999). In brief, lentivirus was generated using HEK 293T cells transfected with FuGENE HD (E2311, Promega) and pCMV-VSVG (Addgene #8454), pRSV-REV (Addgene #12253), pMDLg/pRRE (Addgene #12251) and LV-GFP. Concentrated lentivirus was used to transduce cell lines. Ten days post-transduction, GFP-positive cells were sorted on BD FACSAria Fusion 3 or 5 Flow Cytometers to capture cells within 50–75% brightness. At least 400,000 cells were collected post-sorting, and cell line identity was revalidated by STR profiling.

### siRNA knockdown assays

Attached cells were transfected with siRNA by complexation with DarmaFECT 3 Transfection Reagent (T-2003, Horizon Discoveries) in Opti-MEM (31985062, Gibco). After 24 h, the media were replaced with fresh culture medium with drug treatment or DMSO control. Knockdown efficiency was measured by gene expression by quantitative PCR (qPCR) with reverse transcription or 3′ RNA-seq.

siRNA used were purchased from Horizon Discoveries and are as follows: for *CCND1* knockdown, siRNA targeting *CCND1*-1 (si*CCND1*-1; J-003210-17) and si*CCND1*-2 (J-003210-18); for *KDM5A* knockdown, si*KDM5A-*1 (J-003297-22) and si*KDM5A*-2 (J-003297-23); and for non-targeting control siRNA, siControl (DHA-D-001810-01-05).

### Chromatin profiling

#### Cell line sample preparation

Cells were trypsinized with TrypLE (12604021, Gibco), centrifuged at 400*g* and resuspended in fresh complete medium. Cells were counted manually with a haemocytometer, and an average of two counts per sample were used.

#### CUT&RUN assay, library preparation and sequencing

The CUT&RUN Assay Kit (86652, CST) was used. All buffers were prepared as per the CST CUT&RUN Assay Kit protocol. For each CUT&RUN reaction, 500,000 cells were used. Cells were washed, bound to Con A beads and incubated with primary antibody overnight at 4 °C as per the manufacturer’s instructions. After one wash with digitonin buffer, pA/G-MNase was added to cells for binding at 4 °C for 1 h. Cells were washed twice, and calcium chloride was added to activate the MNase for chromatin digestion at 4 °C for 30–60 min. A modification to the manufacturer’s protocol, 0.1% SDS, proteinase K and 300 mM NaCl were added to the STOP buffer. The modified STOP buffer was added to cells to stop the digestion reaction and release all DNA material. Lysed samples in STOP buffer were incubated at 55 °C for 1 h for cell lines or overnight for tumour tissue, and then centrifuged at room temperature at 16,000*g* for 2 min. Using a magnetic concentrator to collect Con A beads, the supernatant was collected for DNA purification with the DNA Purification Buffers and Spin Columns kit, as per the manufacturer’s protocol (14209, CST). Size selection was performed by adding AMPure XP beads (A63881, Beckman Coulter) to the purified CUT&RUN DNA at 0.5× to remove large DNA fragments. The remainder of DNA fragments were collected and purified by adding an extra 1.3× (to attain a total of 1.8×) AMPure beads. DNA-bound beads were washed with 80% ethanol twice, and DNA fragments were eluted in 10 mM Tris pH 8.0. Concentration and fragment sizes of the DNA were measured using the TapeStation D1000 High Sensitivity kit (5067-5584 and 5067-5585, Agilent) on the Agilent TapeStation 4150.

Primary antibodies used included Rb (clone 4H1; 61121, CST; 1 μg per reaction for cell lines and 1.5 μg for tumour tissue), ER (8644, CST; 0.8 μg), H3K4me3 (9751, CST; 1 μg), KDM5A (ab194286, Abcam; 1 μg), mouse IgG2a (61656, CST; 1 μg per reaction for cell lines and 1.5 μg for tumour tissue) and rabbit IgG (66362, CST; 1 μg).

For library construction, the concentration of DNA was determined from the TapeStation concentration reading between 100 and 700 bp. The NEBNext Ultra II DNA Library Prep Kit (E7645S, NEB) and Unique Dual Indices (E6440S, NEB) were used. We followed the CST DNA Library Prep Kit for Illumina Protocol that was optimized for CUT&RUN, which included modifications to adaptor dilutions, AMPure XP bead ratios, determining the number of PCR amplification cycles, and the PCR anneal and extension step, which had been shortened to 13 s compared with the standard protocol. Amplified DNA was cleaned up using AMPure XP beads and eluted in 10 mM Tris pH 8.0. CUT&RUN libraries were quantified using the TapeStation D1000 (5067-5582 and 5067-5583, Agilent) on the TapeStation, then pooled and sequenced on an Illumina NextSeq 2000 instrument, paired end, obtaining 5–10 million clusters per sample.

#### Tissue sample preparation

Tumours were extracted from euthanized mice and rinsed in PBS. The tissue was first cut longitudinally, then sliced into thin sheets using a microtome blade. The resulting pieces were approximately 4 mm × 4 mm × 1 mm, one piece per tumour. Each piece was used for one single DynaTag reaction.

#### DynaTag assay, library preparation and sequencing

Cleavage under DynaTag, a modified CUT&Tag method adapted from Hunold et al., was used^21^. In brief, tissue slices were washed in DynaTag buffer (25 mM HEPES pH 7.5 (15630-080, Gibco), 110 mM KCl (AM9640G, Invitrogen), 10 mM NaCl (AM9760G, Invitrogen), 1 mM MgCl_2_ (AM9530G, Invitrogen), 1× spermidine (27287S, CST), 1× protease inhibitor cocktail (7012L, CST)), bound to Con A beads, and incubated overnight at 4 °C with primary antibody (Rb (61121, CST) or mouse IgG2a) in antibody buffer (DynaTag buffer + 1% w/v BSA (A8577, Sigma; 10 ml) and digitonin (16359L, CST)). Anti-mouse secondary antibody (0.5 μg; ab46540, Abcam) and pre-loaded pA–Tn5 complex (1:100 dilution; C0107001, Diagenode) binding were performed at room temperature for 1 h each. Tagmentation was initiated by incubating samples at 37 °C for 1 h in antibody buffer supplemented with 10 mM MgCl_2_. Upon tagmentation, samples were resuspended in denaturation buffer (10 mM Tris-HCl (15568-025, Invitrogen), 50 mM NaCl (AM9760G, Invitrogen), 0.2% SDS (15553-035, Invitrogen), 0.15 mg ml^−1^ proteinase K (10012S, CST) and 16.67 mM EDTA (15575020, Invitrogen)) and incubated at 58 °C for 3 h. Denaturation was stopped by purification of the tagmented DNA fragments using the MinElute PCR Cleanup Kit (28004, Qiagen), as per the manufacturer’s protocol. The library was prepared using Illumina DNA/RNA UD Indexes Set A, Tagmentation (20091654, Illumina) and NEBNext 2× PCR Mix (M0541L, NEB). Amplified DNA was cleaned up using AMPure XP beads and eluted in EB buffer. Libraries were quantified using the TapeStation D1000 on the TapeStation, then pooled and sequenced on an Illumina NextSeq 2000 instrument, paired end, obtaining 5–10 million clusters per sample.

### RNA extraction

RNA was extracted using the NucleoSpin RNA plus kit (740984, Macherey-Nagel) per the manufacturer’s protocol.

### Reverse transcription quantitative PCR

RNA was converted to cDNA using the High-Capacity cDNA Reverse Transcription Kit (4368814, Thermo Fisher) as per the manufacturer’s protocol. SYBR Select Master Mix (4472919, Applied Biosystems) was used for the qPCR, which was run on an Applied Biosystems StepOne Plus instrument. The ^ΔΔ^Ct method was used to analyse the data. Primer sequences used for qPCR were as follows: *KRT18* forward: 5′-TCG CAA ATA CTG TGG ACA ATG C, reverse: 5′-GCA GTC GTG TGA TAT TGG TGT; *MKI67* forward: 5′-AGA AGA AGT GGT GCT TCG GAA, reverse: 5′-AGT TTG CGT GGC CTG TAC TAA; *TK1* forward: 5′-GCC AAA GAC ACT CGC TAC AG, reverse: 5′-CCC CTC GTC GAT GCC TAT G; *CCND1* forward: 5′-GCT GCG AAG TGG AAA CCA TC, reverse: 5′-CCT CCT TCT GCA CAC ATT TGA A; and *HSP90AB1* (housekeeping) forward: 5′-AGA AAT TGC CCA ACT CAT GTC C; reverse: 5′-ATC AAC TCC CGA AGG AAA ATC TC.

### RNA library preparation and sequencing

Libraries were prepared using the Quanteq Lexoen 3′ mRNA FW kit by the Peter MacCallum Cancer Centre Molecular Genomics Core. Single-end 100-bp reads were sequenced on a NextSeq 2000 instrument (Illumina), with a goal of 3–5 million reads per sample.

### Western blotting

#### Protein lysate preparation

For the detection of ER, protein lysates were prepared using 1× cell lysis buffer (CST) as previously described^23^. For the detection of Rb and KDM5A expression, lysates were prepared by lysing equal numbers of cells in 4× Laemmli sample buffer (1610747, Bio-Rad) diluted to 1× with water and with 5% β-mercaptoethanol added and heated to 95 °C for 13 min.

#### Western blotting

Western blotting was performed as previously described^23^. Primary antibodies used include Rb (554136, BD; 1:1,000), phospho-Rb S780 (8180, CST; 1:1,000), phospho-Rb S807/811 (8516, CST; 1:1,000), ER (8644, CST; 1:1,000), KDM5A (ab194286, Abcam; 1:5,000), histone H3 (9715, CST; 1:1,000), cyclin D1 (55506, CST; 1:1,000), KRT18 (4548, CST; 1:1,000), TFF1 (15571, CST; 1:1,000), cyclin A2 (ab32386, Abcam; 1:1,000), actin (A3854, Sigma-Aldrich; 1:10,000) and vinculin (V9131, Sigma-Aldrich; 1:5,000). Secondary antibodies used include StarBright Blue 520 goat anti-mouse IgG (12005867, Bio-Rad) and StarBright Blue 700 goat anti-rabbit IgG (12004162, Bio-Rad), both at 1:2,500. Western blot images were acquired on ChemiDoc MP Imaging System (Bio-Rad) and visualized using Image Lab Software (v6.1).

### Immunohistochemistry

Tumour tissue extracted from euthanized mice were immediately fixed in 10% neutral-buffered formalin overnight and stored in 70% ethanol. Fixed tissue pieces were dehydrated with ethanol and xylene and embedded in paraffin. The embedded tissues were sectioned at 10 μm thickness and dewaxed, rehydrated and subject to heat-mediated antigen retrieval in citrate buffer pH 6.0. After washing, samples were incubated in 3% hydrogen peroxide for 5 min, washed again and incubated for 1 h at room temperature in primary antibodies to Ki-67 (ab16667, Abcam), phospho-Rb Ser807/811 (8516, CST) and total-Rb (554136, BD Biosciences). Secondary antibodies were ImmPRESS anti-rabbit (MP-7451-NB, Novus) or ImmPRESS anti-mouse (MP-7452-NB, Novus). Signal development was performed using the Dako Liquid DAB^+^ Substrate Chromogen System (K3468, Agilent Technologies) before counterstaining with haematoxylin. Whole-slide images were acquired using an Olympus SLIDEVIEW VS200 microscope. The number of positive tumour cells was calculated using QuPath^45^.

### Nuclear co-immunoprecipitation

Nuclear co-immunoprecipitation (co-IP) was performed using the Active Motif Nuclear Complex Co-IP kit (54001, Active Motif) as per the manufacturer’s protocol. In brief, nuclei were extracted with the hypotonic buffer. Nuclear lysates were prepared using the digestion cocktail. Lysates were cleared using mouse IgG2a (61656, CST) and rabbit IgG (ab37415, Abcam) bound to protein A/G Dynabeads (10002D and 10004D, Thermo Fisher), incubated at 4 °C for 1–3 h. Cleared lysates were incubated with primary antibody in 1× low buffer overnight at 4 °C, and protein A/G Dynabeads were added to the lysate for another 1 h at 4 °C. The beads were washed for a total of six times using the 1× low buffer. Proteins were eluted using 4× Laemmli sample buffer (1610747, Bio-Rad) diluted to 2× with water and with 10% (v/v) β-mercaptoethanol added, and heated to 95 °C for 5 min. Primary antibodies used for co-IP include Rb (9039, CST), ER (8644, CST), mouse IgG2a (61656, CST) and rabbit IgG (ab37415, Abcam).

### Mass spectrometry

#### Peptide preparation

Samples eluted from nuclear co-IP were reduced with 5 mM TCEP (646547-10X1ML, Merck), alkylated with 20 mM methyl methanethiosulfonate (23011, Thermo Fisher) and acidified using phosphoric acid (695017-100ML, Sigma-Aldrich). Samples were then loaded onto S-Trap Micro Spin Columns, following the manufacturer’s instruction (Protifi) and digested with trypsin (90057, Thermo Fisher) overnight at 37 °C. Peptides were sequentially eluted with 50 mM Tris-HCl (pH 8.5), 0.2% formic acid (695076-100ML, Sigma-Aldrich) and 50% acetonitrile (1000291000, Merck). Eluates were snap frozen in liquid nitrogen and freeze dried overnight. Immediately before mass spectrometry, samples where resuspended in 30–40 μl of resuspension buffer (2% acetonitrile + 0.05% trifluoroacetic acid in mass spectrometry grade water) and briefly sonicated in a water bath. Samples were centrifuged at maximum speed for 10 min, and the top 15 μl of each supernatant was collected for analysis.

#### Data-independent acquisition

Liquid chromatography–tandem mass spectrometry analyses were performed on an Orbitrap Ascend mass spectrometer (Thermo Fisher Scientific) as previously described^46^. The liquid chromatography system (Ultimate 3000) was equipped with an Acclaim PepMap nano-trap column (Dionex C18, 100 Å, 75 µm × 2 cm) and an Acclaim PepMap RSLC analytical column (Dionex C18, 100 Å, 75 µm × 50 cm). Tryptic peptides were loaded onto the enrichment column at an isocratic flow rate of 5 μl min^−1^ with 2% (v/v) acetonitrile containing 0.05% (v/v) trifluoroacetic acid for 6 min, after which the enrichment column was switched in-line with the analytical column. The eluents were 0.1% (v/v) formic acid in water (solvent A) and 100% (v/v) acetonitrile with 0.1% formic acid (solvent B), both supplemented with 5% DMSO. The gradient was run at 300 nl min^−1^ as follows: (1) 0–6 min, 3% B; (2) 6–7 min, 3–4% B; (3) 7–82 min, 4–25% B; (4) 82–86 min, 25–40% B; (5) 86–87 min, 40–80% B; (6) 87–90 min, 80–3% B; and (7) 90–90.1 min, 80–3% B, followed by equilibration at 3% B for 10 min before the next injection.

For data-independent acquisition (DIA) experiments, full mass spectrometry scans were acquired at a resolution of 120,000 (at *m*/*z* 200), with a scan range of 350–1,400 *m*/*z* in profile mode. The full mass spectrometry AGC target was set to 250% with an injection time of 50 ms. The AGC target for fragment spectra was 2,000%. Fragmentation was performed across 50 windows of 13.7 Da width with a 1-Da overlap. MS2 resolution was set to 30,000 with a maximum injection time of 55 ms. Normalized collision energy was set to 30%, and data were acquired in centroid mode with positive-ion polarity.

### Bioinformatic analysis

#### RNA-seq

Fastq files were trimmed for adapters and low-quality reads using BBDuk (v38.90) via the bbduk.sh script^47^ with the following parameters: ktrim = r k = 23 mink = 11 hdist = 1 tpe tbo qtrim = r trimq = 5 minlen = 20. Trimmed reads were aligned to the hg38 reference genome (GRCh38.p15; https://www.ncbi.nlm.nih.gov/assembly/GCA_000001405.15) using STAR (v2.7.5b)^48^. For PDX samples, mouse reads were filtered out using XenofilteR (v1.6)^49^. Read counts were generated using featureCounts from the Subread package (v2.0.1)^50^, with annotations from GENCODE (gencode.v35.annotation.gtf^51^). Normalization was performed using the median-of-ratios size-factor method implemented in DESeq2. Differential gene expression analysis was performed using DESeq2 (v1.46.0)^52^. Genes with an adjusted *P* < 0.05 were considered differentially expressed.

Gene expression heatmaps were generated using ComplexHeatmap (v2.22.0)^53^. Gene-wise *z*-scores were calculated from DESeq2 size-factor-normalized read counts and used for heatmap visualization. Genes were clustered by hierarchical clustering using Euclidean distance and complete linkage.

#### Microarray data

Raw microarray data for palbociclib-treated patients and untreated control patients were accessed from our previous publication^27^ (http://microarrays.curie.fr/publications/U981-GustaveRoussy/pop/). All participants provided written informed consent before study participation. Ethical approval and trial conduct have been previously described^27^. The dataset was filtered to include only ER-positive and human epidermal growth factor receptor 2 (HER2)-negative patients, resulting in 18 control patients and 44 patients treated with palbociclib. Raw CEL files were imported into R using the read.celfiles function from the oligo R package. Data normalization was performed using the robust multichip average algorithm implemented in the oligo package with the parameter target = ‘core’. Probeset annotations were mapped to gene symbols using the hugene21sttranscriptcluster.db annotation package via the annotateEset function from the affycoretools R package. Quality control filtering was applied to remove lowly expressed genes using a median intensity threshold of 1.5, determined through visual inspection of transcript median distributions in histogram plots^54^. Differential expression analysis was conducted using the limma R package^55^ to identify genes significantly altered between control and palbociclib-treated groups.

#### GSEA of transcriptomics datasets

GSEA was conducted using clusterProfiler (v4.14.6)^56^. Hallmark gene sets were sourced from the Molecular Signatures Database (MSigDB)^25,26^ via msigdbr (v24.1.0). For cell line and PDX RNA-seq datasets, genes were ranked by shrunken log_2_ fold changes calculated using the adaptive shrinkage ‘ashr’ method. For the POP clinical transcriptomics dataset, genes were ranked by the Wald statistic. Enrichment scores were computed using the running-sum statistic implemented in GSEA. Single-sample GSEA was performed using GSVA (v2.0.7) with α = 0.25.

#### CUT&RUN and DynaTag

Adapters and low-quality reads were removed from Fastq files using BBDuk (v38.90)^47^ with the following parameters: ktrim = r k = 23 mink = 11 hdist = 1 tpe tbo qtrim = r trimq = 5 minlen = 20. Trimmed reads were aligned to hg38 (GRCh38.p15; https://www.ncbi.nlm.nih.gov/assembly/GCA_000001405.15) using Bowtie2 (v2.3.4.1) with the following parameters: –dovetail–local–very-sensitive–no-mixed–no-discordant–phred33 -I 10 -X 700. For PDX DynaTag samples, trimmed reads were aligned to a hybrid genome consisting of hg38 (GCA) and mm10 (UCSC). The mm10 genome FASTA and annotation files were obtained from UCSC (https://hgdownload.soe.ucsc.edu/goldenPath/mm10/bigZips/). Reads aligning to ChIP blacklisted regions or mitochondrial DNA were removed using Picard (v3.0.0)^57^, samtools (v1.9)^58^ and bedtools (v2.27.1)^59^. Duplicate reads were retained for downstream analysis.

Peaks were called using MACS2 (v2.2.7.1)^60^ callpeak with the matched IgG controls specified using the–control parameter and the options–format BAMPE -gsize hs -q 0.05. Broad H3K4me3 peaks were called using MACS2 callpeak with the options–format BAMPE -g hs –broad–broad-cut-off 0.1 -q 0.05, using matched IgG controls as input.

Differential analysis was performed using DiffBind (v3.16.0)^61^, with relative log expression (RLE) background normalization using the following parameters: method = DBA_DESEQ2, normalize = DBA_NORM_RLE, library = DBA_LIBSIZE_BACKGROUND, background = TRUE. Differential binding was defined using a false discovery rate-adjusted *P* value threshold of 0.1 for CUT&RUN datasets and 0.2 for DynaTag datasets.

Peaks were annotated to genomic features using ChIPseeker (v1.42.0)^62^. Promoters were defined as regions spanning −2,000 bp to +500 bp relative to the transcription start site (TSS). Peaks overlapping promoter regions were classified as promoter peaks, and all remaining peaks were classified as non-promoter peaks.

BigWig files were generated using the bamCoverage function from deepTools with the following parameters: -binSize 20, -smoothLength 60, -scaleFactor scalefactor, -extendReads, where scale factors were derived from DiffBind RLE background normalization. BigWig files for negative input, H3K4me3 and H3K4me1 samples were normalized using bins per million mapped reads. BigWig tracks were visualized using the Integrative Genomics Viewer^63^ or deepTools.

Motif enrichment analysis was carried out using HOMER (v4.11)^64^ with the parameters; -size 200 -len 6,10,15,20 -p 14. Enrichment ratios represent motif frequency in the input regions relative to HOMER-generated random genomic background.

GSEA of genomic regions was conducted using ChIP-Enrich (v2.30.0)^24^, assigning each peak region to the gene whose TSS was nearest to the peak midpoint. Gene set over-representation analysis (ORA) was performed using clusterProfiler (v4.14.6)^56^ on genes located within 100 kb of the genomic regions of interest and significantly upregulated by treatment (log_2_ fold change > 0, adjusted *P* < 0.05). MSigDB hallmark gene sets were used in both methods. Odds ratios represent the observed versus expected frequency of genes associated with the genomic regions of interest within each gene set.

To assess the transcriptional regulatory function of Rb-bound non-promoter regions, Rb CUT&RUN and RNA-seq data were integrated using BETA (v1.0.7)^22^. Genes were ranked according to regulatory potential scores calculated by BETA based on the proximity and abundance of Rb-bound regions relative to gene promoters. The regulatory potential distributions of upregulated and downregulated genes were compared using the one-tailed Kolmogorov–Smirnov test implemented in BETA.

#### Chromatin interaction and transcriptional hub analysis

H3K27ac HiChIP loop data from our previous study (GSE157381)^23^ were used for these analyses. Processed loop calls, binned into 5-kb genomic regions, were obtained from the published dataset. To identify chromatin interactions associated with chromatin-bound Rb, each 5-kb region was annotated for overlap with Rb CUT&RUN peaks using a minimum threshold of 1 bp. Rb-bound regions were classified as promoter or non-promoter based on the genomic annotation of the overlapping Rb peak. Regions overlapping both promoter and non-promoter Rb peaks were classified as promoter regions.

For HiChIP ORA, gene set ORA using MSigDB hallmark gene sets was performed on protein-coding genes whose promoters were directly connected to non-promoter Rb regions through H3K27ac HiChIP interactions. To assess co-occupancy with ER, all 5-kb genomic windows were annotated for overlap with ER CUT&RUN peaks using a minimum overlap threshold of 1 bp.

Transcriptional hubs were identified from the H3K27ac HiChIP interaction network by constructing an igraph object with 5-kb regions as nodes and HiChIP interactions as edges. Network communities were detected using the Louvain clustering algorithm implemented in the igraph (v2.1.4)^65^, with default parameters. For hubs ORA, protein-coding genes located within transcriptional hubs containing at least one non-promoter Rb peak were subjected to gene set ORA using MSigDB hallmark gene sets. Hub visualizations were generated using ggraph (v2.2.2).

To identify hubs associated with KDM5A, ER and Rb, hubs were required to contain at least one non-promoter Rb up peak overlapping a KDM5A consensus peak (more than 1 bp) and at least one ER peak (promoter or non-promoter) within the hub. Protein-coding genes with promoters located within these hubs were subjected to gene set ORA. For promoter H3K4me3 width analysis, this gene set was further restricted to genes with promoter-associated H3K4me3 broad peaks present in all samples and overlapping the TSS.

#### Mass spectrometry

DIA data were analysed using the direct DIA workflow with default settings in Spectronaut (v19.9) against the reviewed UniProt *Homo sapiens* database (downloaded October 2024). Trypsin digestion was specified with up to two missed cleavages. Methyl methanethiosulfonate alkylation of cysteine was set as a fixed modification, whereas N-terminal acetylation and methionine oxidation were specified as variable modifications. Protein and peptide-spectrum match (PSM) identifications were filtered at a 1% false discovery rate. Precursor filtering was based on *Q* value, quantification was performed at the MS2 level and cross-run normalization was set to automatic.

For differential binding analysis, raw protein group quantity data from Spectronaut were processed as follows. Proteins with two or three NaN values in both immunoprecipitation conditions were excluded. Remaining NaN values were imputed using one of two methods: (1) if one replicate was missing, the average of the other two replicates within the same condition was used; (2) if only one replicate in the treatment arm had a value, the two NaN values were imputed as zero. log_2_-transformed quantity values were then analysed for differential binding using the limma package (v3.62.2)^55^ with empirical Bayes moderation, and results were visualized with ggplot2.

#### Other statistical analyses

For datasets that passed the normality test, a two-tailed unpaired Student’s *t*-test or one-way ANOVA followed by Bonferroni’s or Tukey’s multiple comparisons test was used. A two-tailed Wilcoxon rank-sum test was applied to non-normally distributed or ranked data.

Information on replication relevant to each figure is included in the legends. No statistics were used to predetermine sample sizes.

### Reporting summary

Further information on research design is available in the Nature Portfolio Reporting Summary linked to this article.

## Online content

Any methods, additional references, Nature Portfolio reporting summaries, source data, extended data, supplementary information, acknowledgements, peer review information; details of author contributions and competing interests; and statements of data and code availability are available at https://doi.org/10.1038/s41586-026-10886-w.

## Supplementary information

Supplementary Information Supplementary Figs 1–4, including uncropped western blots corresponding to the immunoblots presented in the main and Extended Data figures, and representative flow cytometry gating strategies.

Reporting Summary

Supplementary Tables Supplementary Tables 1–6.

Peer Review File

## Source data

Source Data Fig. 1

Source Data Fig. 2

Source Data Fig. 3

Source Data Fig. 4

Source Data Extended Data Fig. 1

Source Data Extended Data Fig. 2

Source Data Extended Data Fig. 3

Source Data Extended Data Fig. 4

Source Data Extended Data Fig. 5

Source Data Extended Data Fig. 6

Source Data Extended Data Fig. 7

## Data Availability

Sequencing datasets generated in this study, including raw and processed data, are available at the Gene Expression Omnibus (GEO) repository under SuperSeries accession GSE303576. Mass spectrometry proteomics data generated in this study have been deposited to the ProteomeXchange Consortium via the PRIDE^66^ repository under accession PXD066496. Previously published H3K27ac ChIP–seq and HiChIP datasets (GSE157222 and GSE157381 (ref. ^23^)) used in this study are publicly available through the GEO. Reference genomes hg38, mm10 and sacCer3 were downloaded from https://www.ncbi.nlm.nih.gov/assembly/GCA_000001405.15, http://hgdownload.cse.ucsc.edu/goldenPath/hg19/bigZips and https://ftp.ncbi.nlm.nih.gov/genomes/all/GCF/000/146/045/GCF_000146045.2_R64/GCF_000146045.2_R64_genomic.fna.gz, respectively. Gene expression data from the POP trial samples are publicly available (http://microarrays.curie.fr/publications/U981-GustaveRoussy/pop/). Source data are provided with this paper.

## Code availability

Data were analysed using publicly available software. The custom code used for sequencing analyses and generation of figures and tables is available on GitHub (https://github.com/Goel-Laboratory/Rb-transcription-limits-tumour-suppression) and has been archived on Zenodo^67^.
