# Supplementary material for: Rb-driven transcription limits its tumour-suppressive effects in breast cancer
Source: Nature. 2026 Aug 12;657(8131):517–24. doi: 10.1038/s41586-026-10886-w (PMC13558077; doi:10.1038/s41586-026-10886-w)
Supplement: Supplementary file 1 [file 41586_2026_10886_MOESM1_ESM.pdf]

nature portfolio

https://doi.org/10.1038/s41586-026-10886-w

Supplementary information

Rb-driven transcription limits its tumour-suppressive effects in breast cancer

In the format provided by the authors and unedited

Supplementary Fig. 1 I Original uncropped western blot images.

Fig. 4b

Rb IP

All samples were run on the same gel, and total Rb, ER, and KDM5A were immunoblotted on the same membrane.

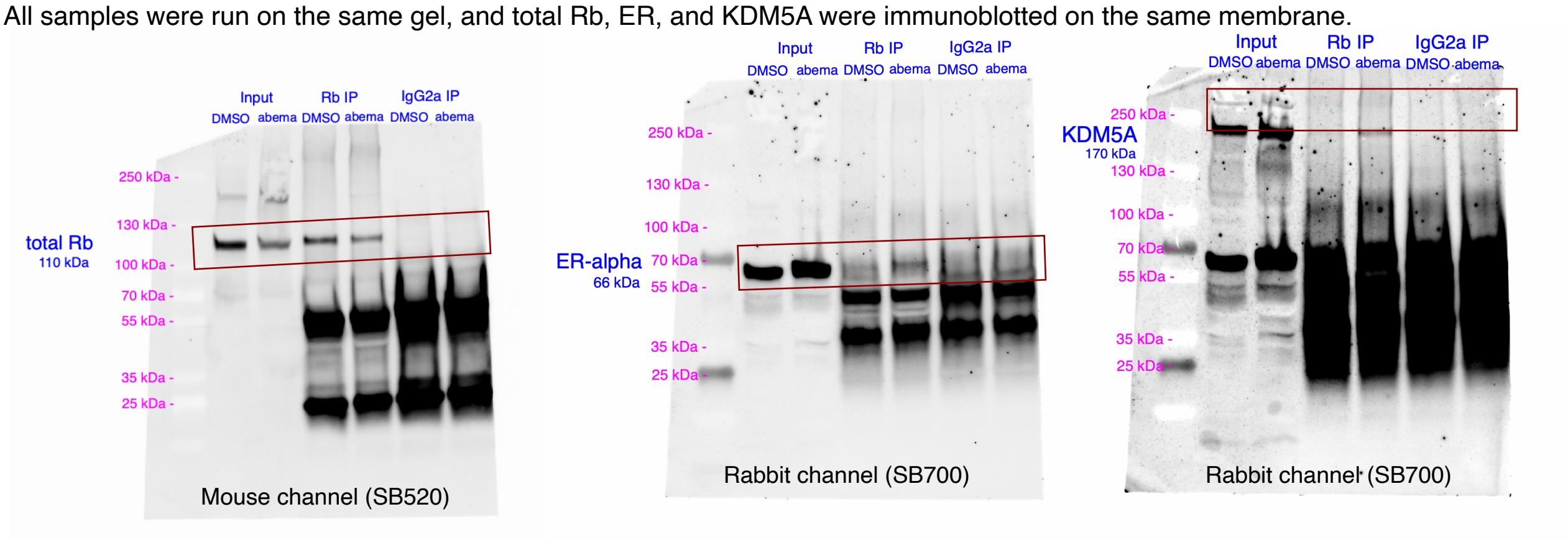

KDM5A IP

All samples were run on the same gel, and total Rb, ER, and KDM5A were immunoblotted on the same membrane.

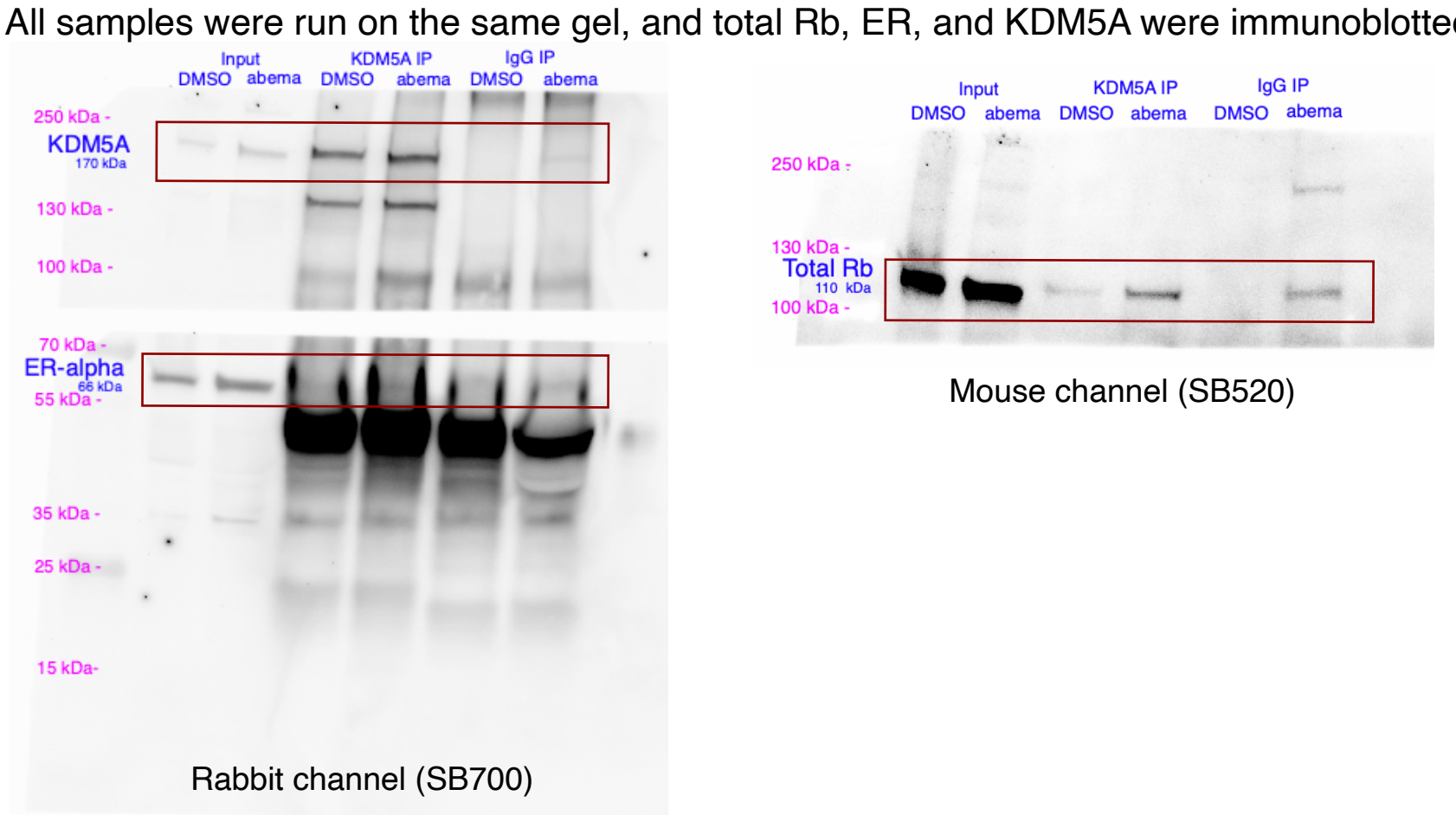

ER IP

All samples were run on the same gel, and total Rb, ER, and KDM5A were immunoblotted on the same membrane.

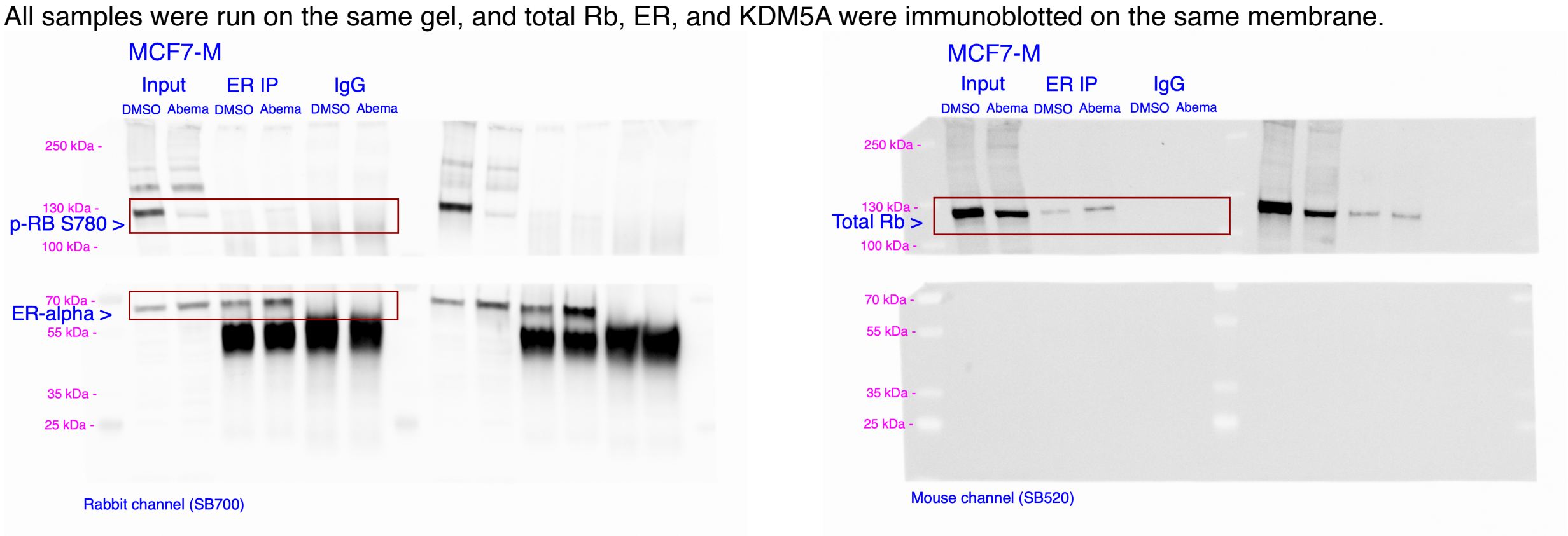

All samples were loaded by equal number of nuclei.

Supplementary Fig. 1 (Continued)

Extended Data Fig. 1b

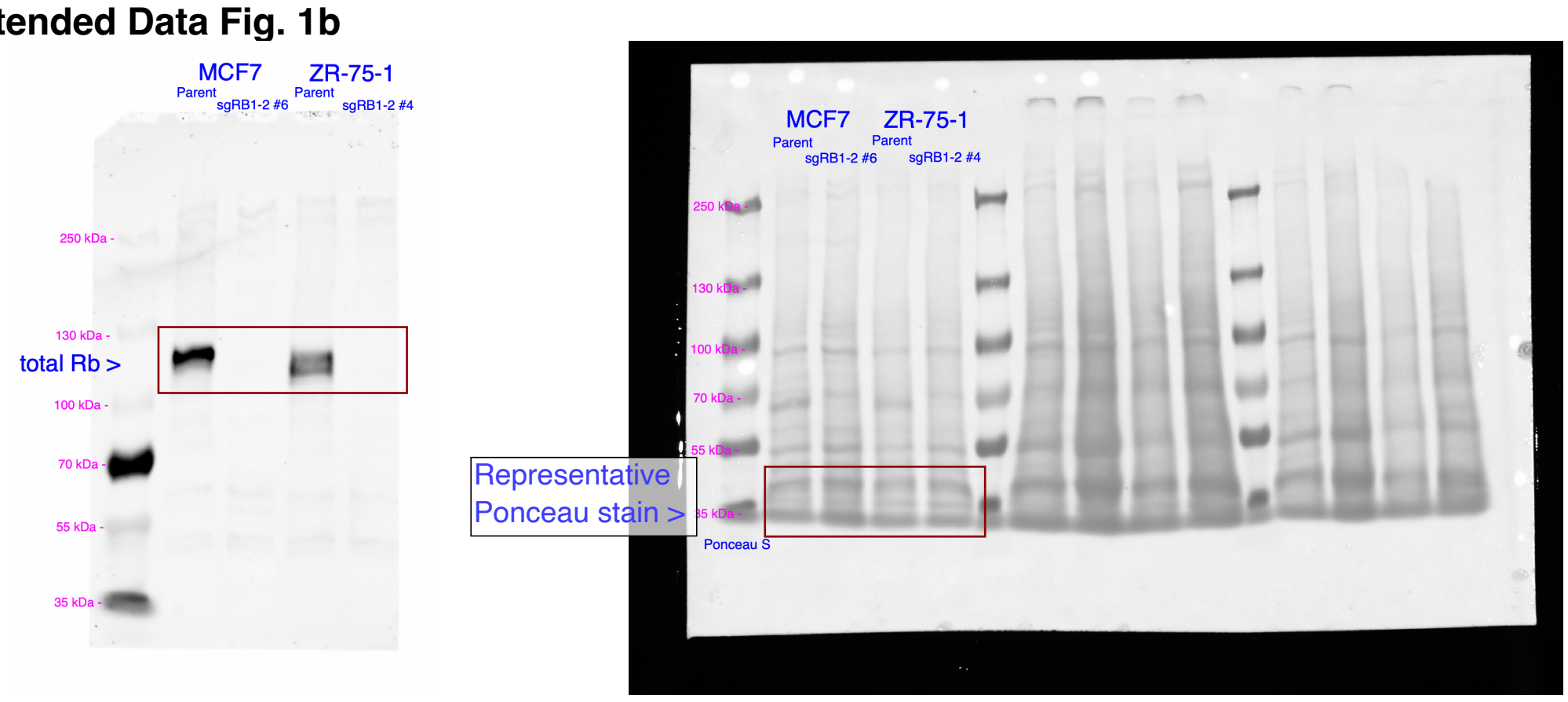

All four samples were loaded onto the same gel and were loaded by equal number of cells. Loading control is total protein stained by Ponceau S on the same membrane.

Extended Data Fig. 1c

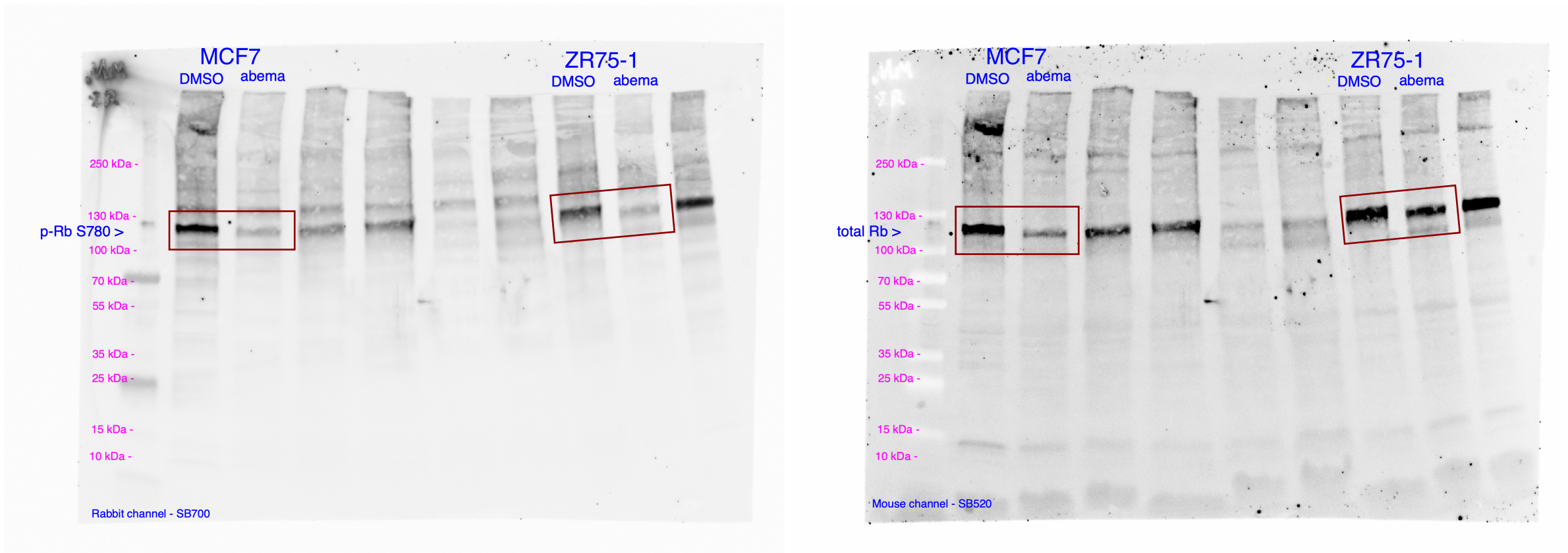

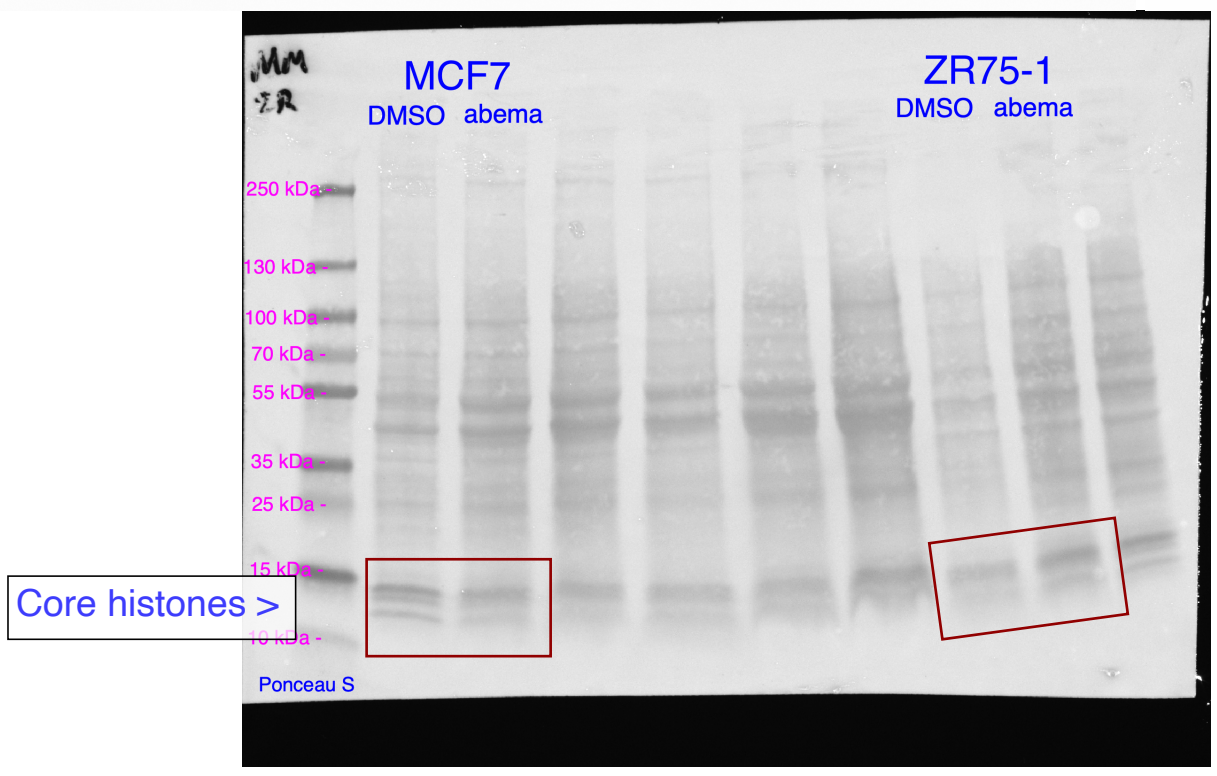

All samples were loaded onto the same gel, and were loaded by equal number of cells. Loading control is core histones stained by Ponceau S on the same membrane.

Extended Data Fig. 1g

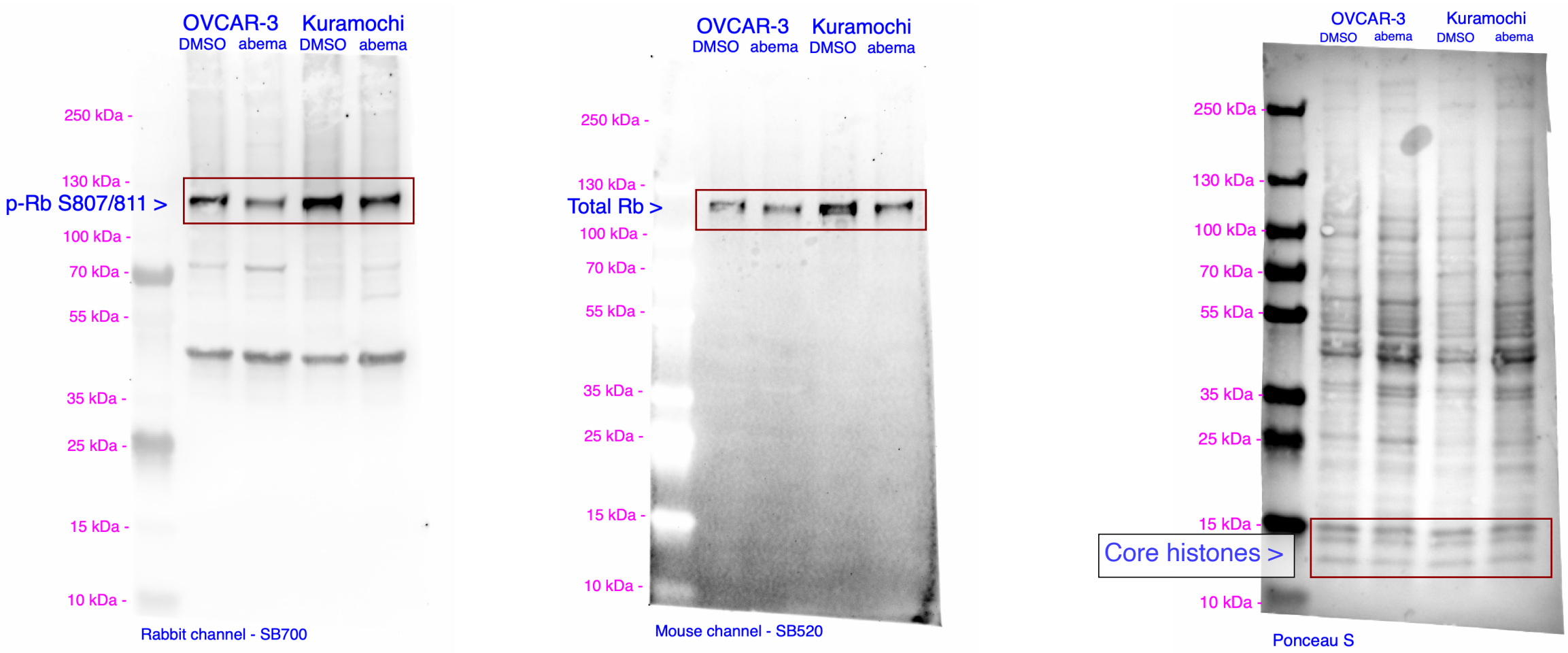

All samples were loaded onto the same gel, and samples were loaded by equal number of cells. pRb S807/S811 and total Rb were multiplex on the same membrane. Loading control is core histones stained by Ponceau S of the same membrane.

Supplementary Fig. 1 (Continued)

Extended Data Fig. 4c

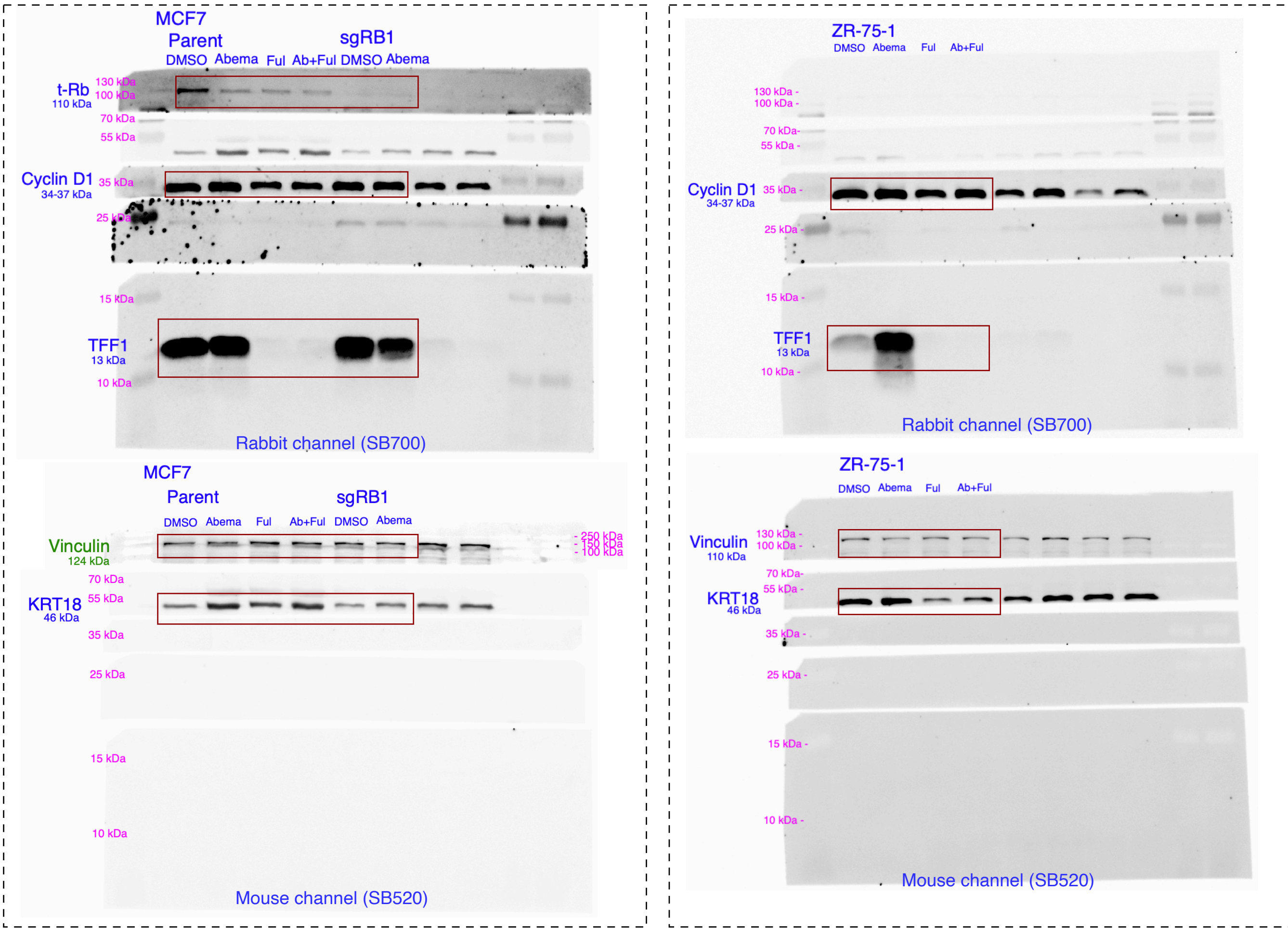

Samples were loaded by equal total protein. For each cell line, all samples were run on the same gel, and the total Rb, cyclin D1, TFF1, KRT18 and loading control vinculin were immunoblotted on the same membrane.

Extended Data Fig. 4g

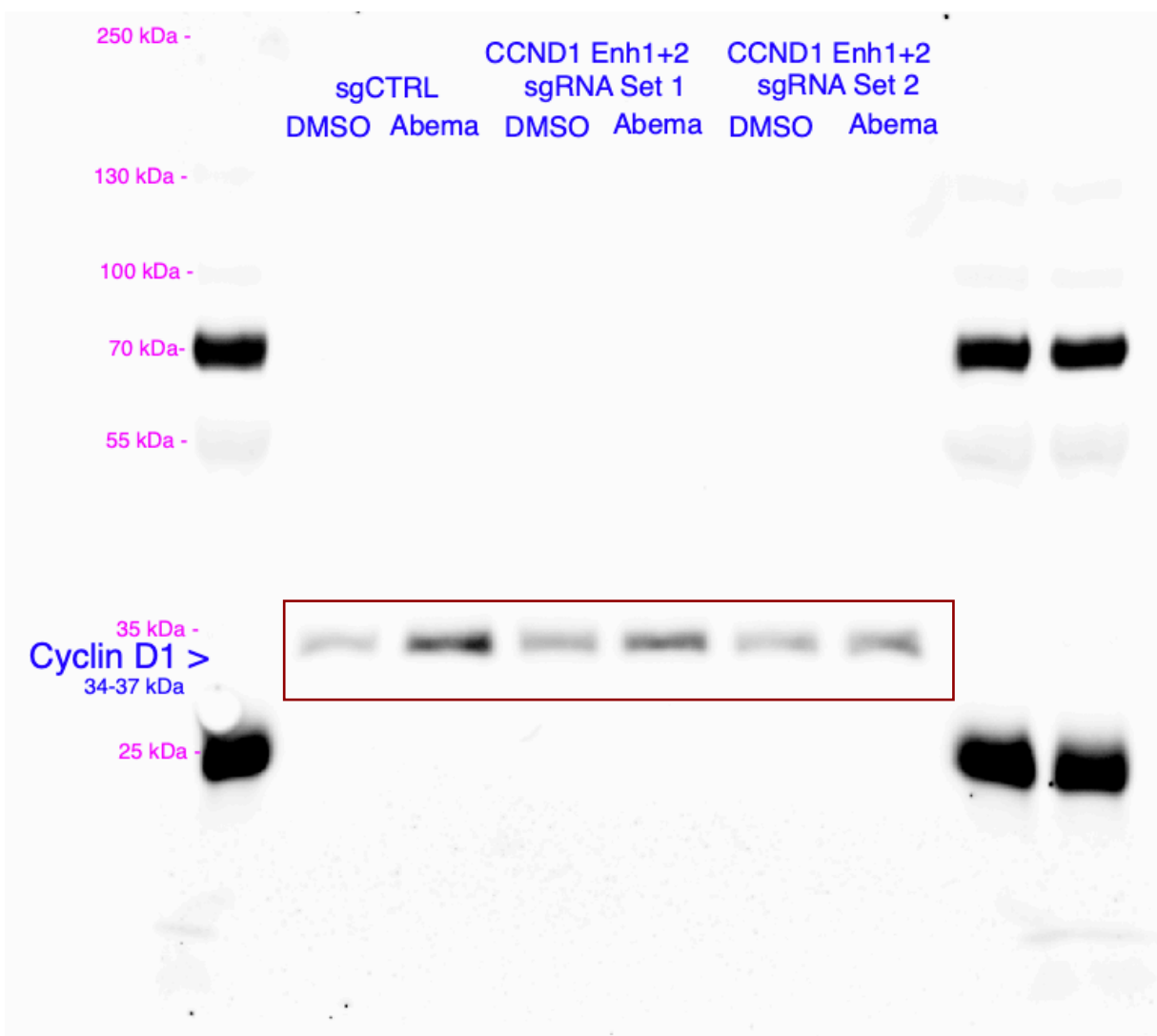

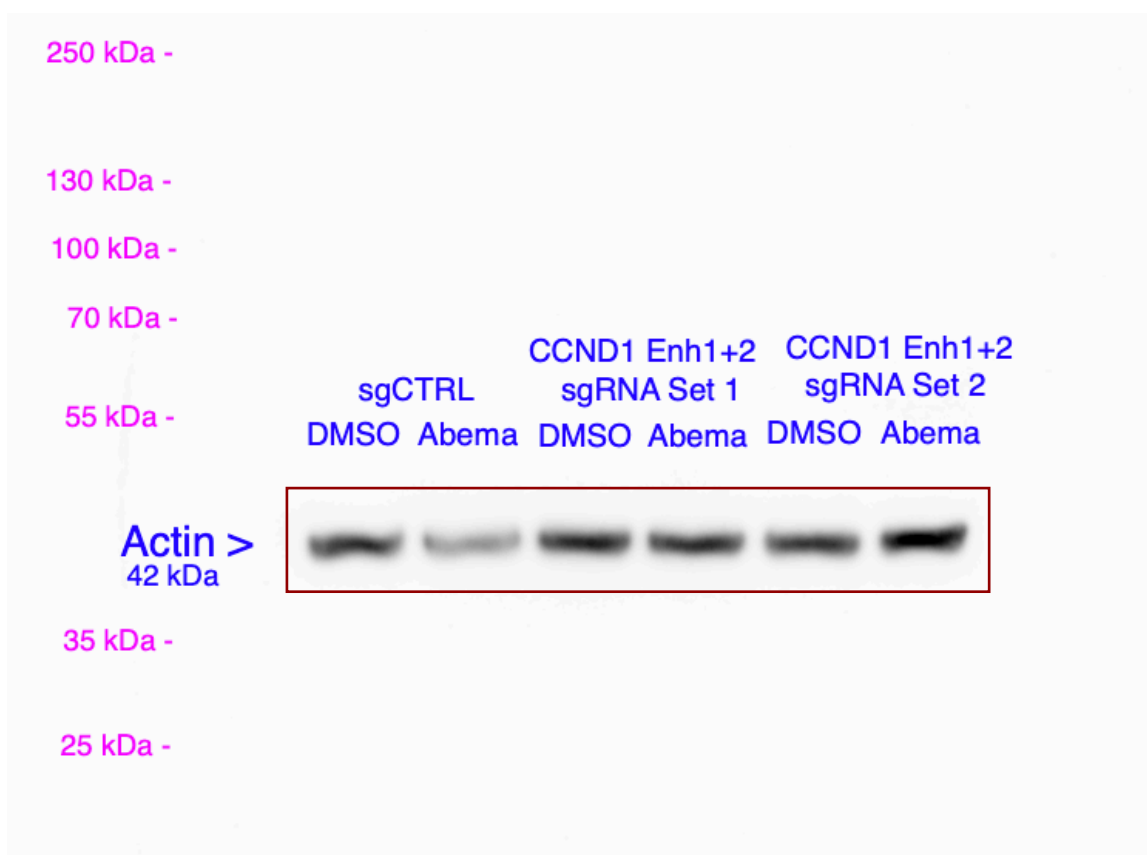

All 6 samples were loaded onto the same gel, and samples were loaded by equal total protein. Loading control is actin staining on the same membrane.

Supplementary Fig. 1 (Continued)

Extended Data Fig. 4h

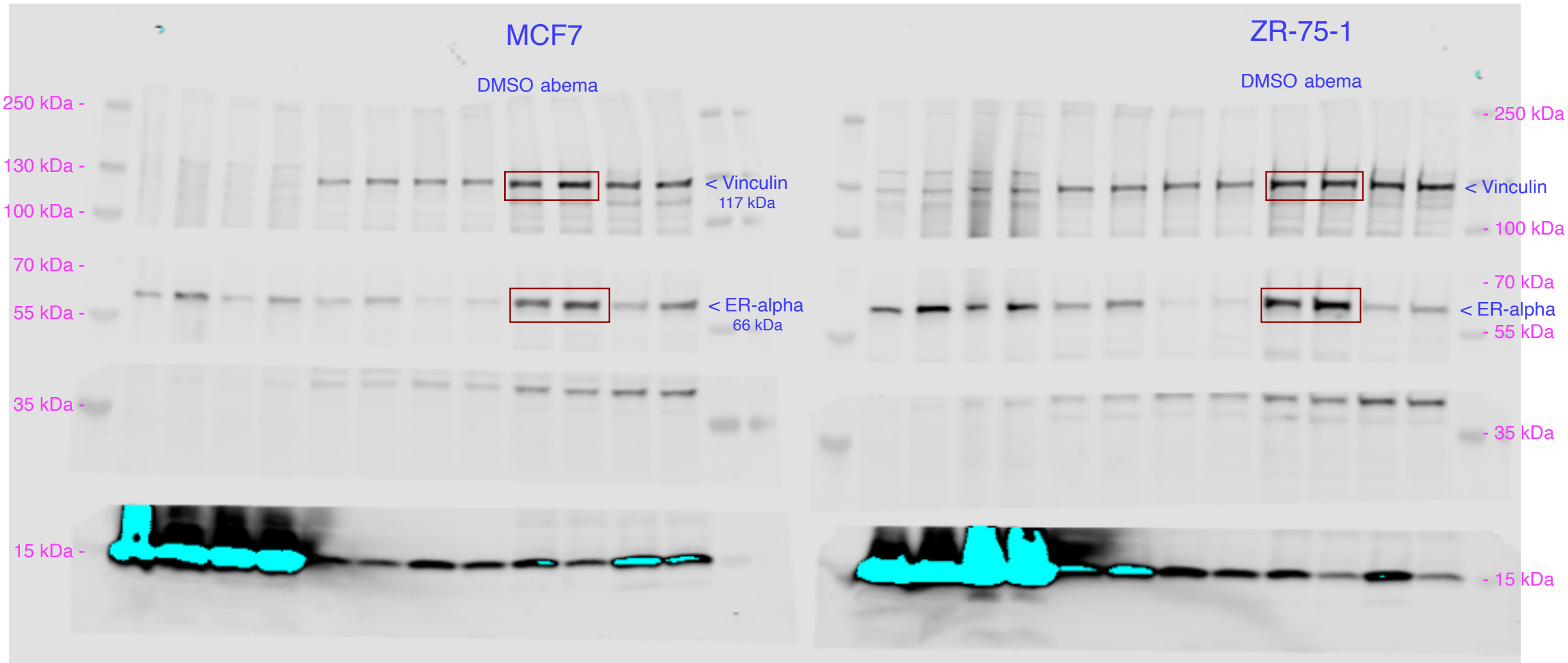

For each cell line, the samples were loaded onto the same gel and equalised by total protein. Loading control is vinculin staining on the same membrane.

Extended Data Fig. 6c

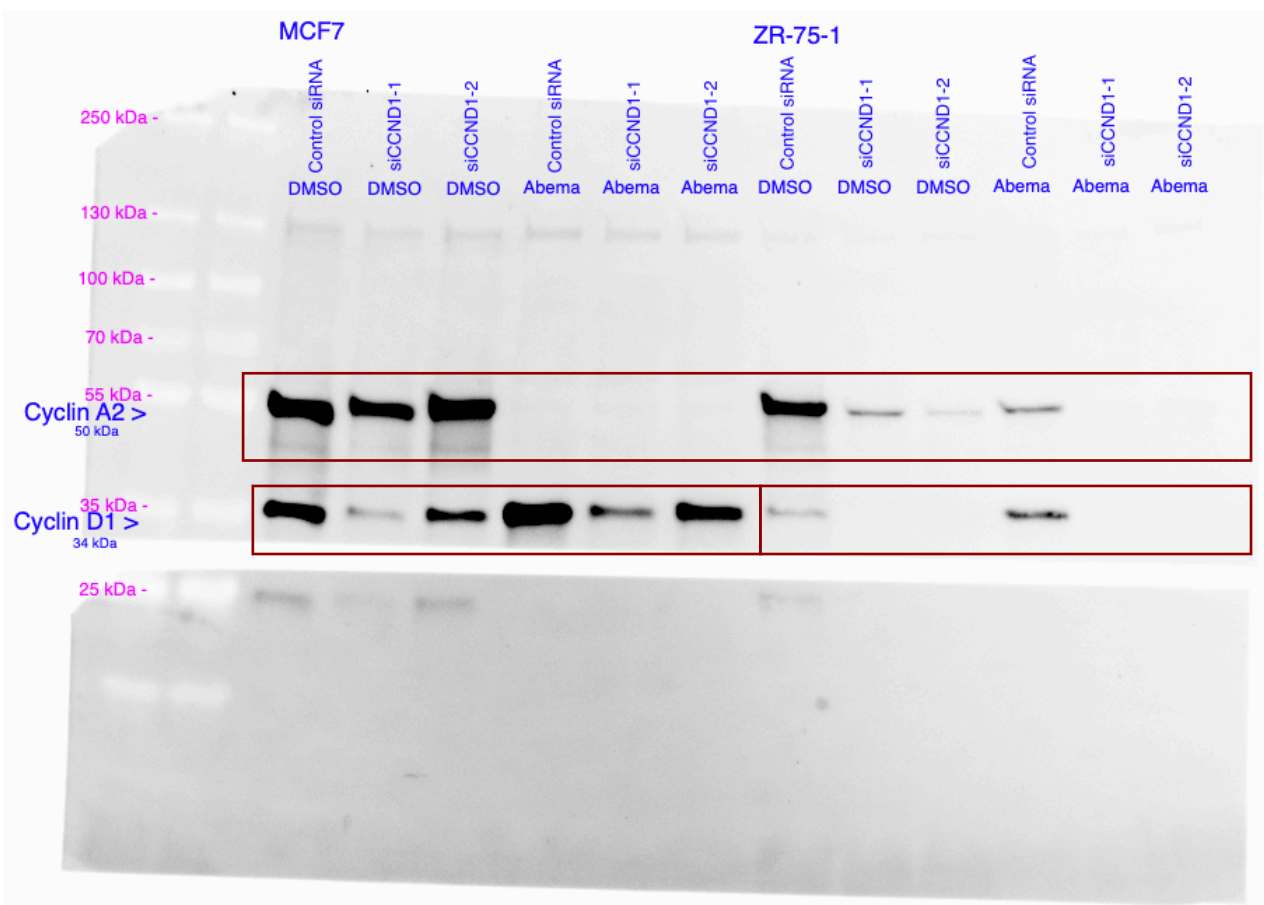

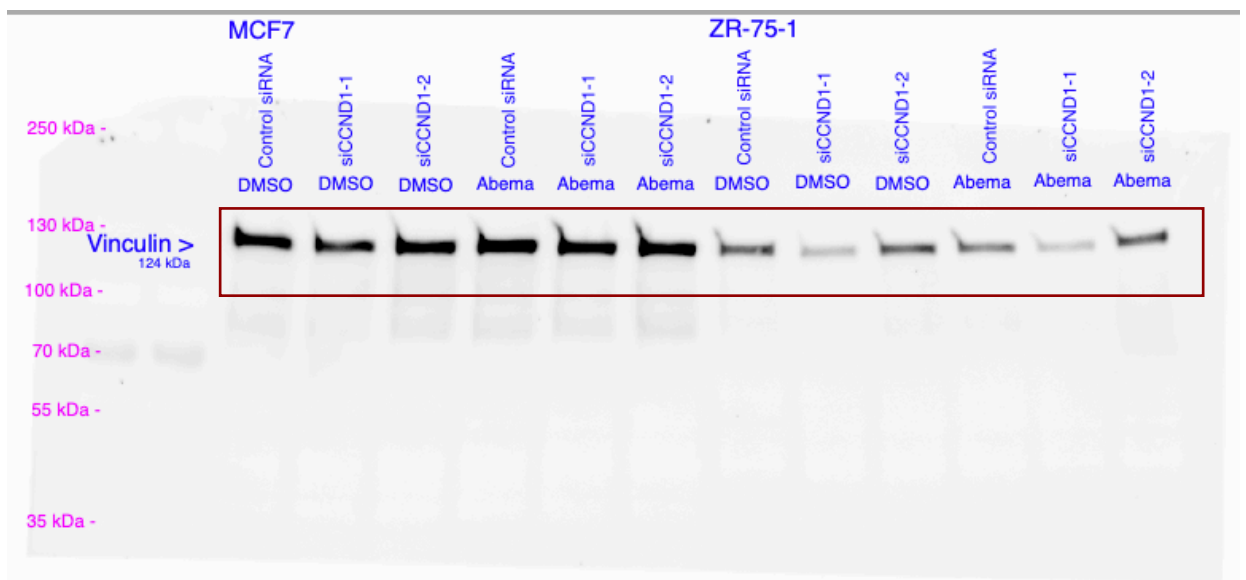

All samples were loaded on the same gel. Samples were loaded by equal total protein. Cyclin A2, cyclin D1, and the loading control vinculin staining were done on the same membrane.

Extended Data Fig. 7a

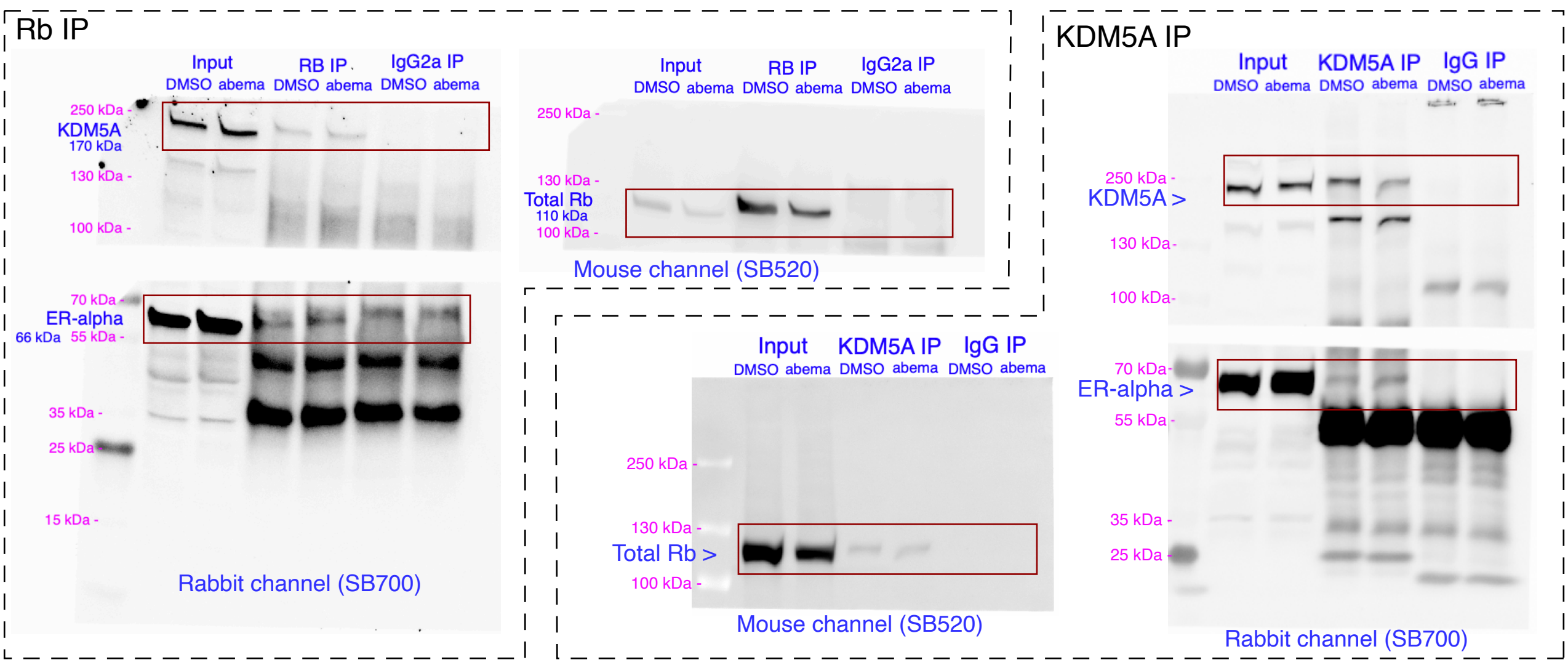

For each IP, the Inputs and IP samples were loaded onto the same gel. Samples were loaded by equal number of nuclei. KDM5A, ER, and total Rb were immunoblotted on the same membranes.

Supplementary Fig. 1 (Continued)

Extended Data Fig. 7c

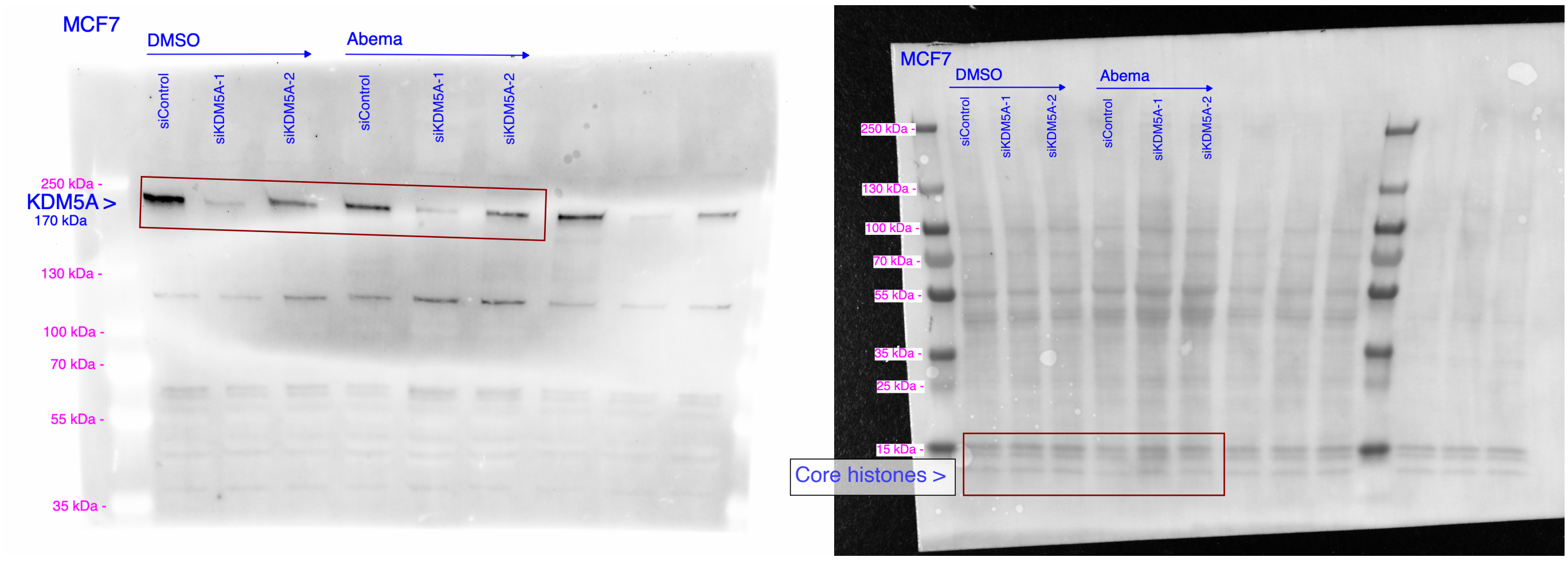

All 6 samples were loaded onto the same gel and equalised by number of cells. Loading control is core histones stained by Ponceau S on the same membrane.

Extended Data Fig. 7f

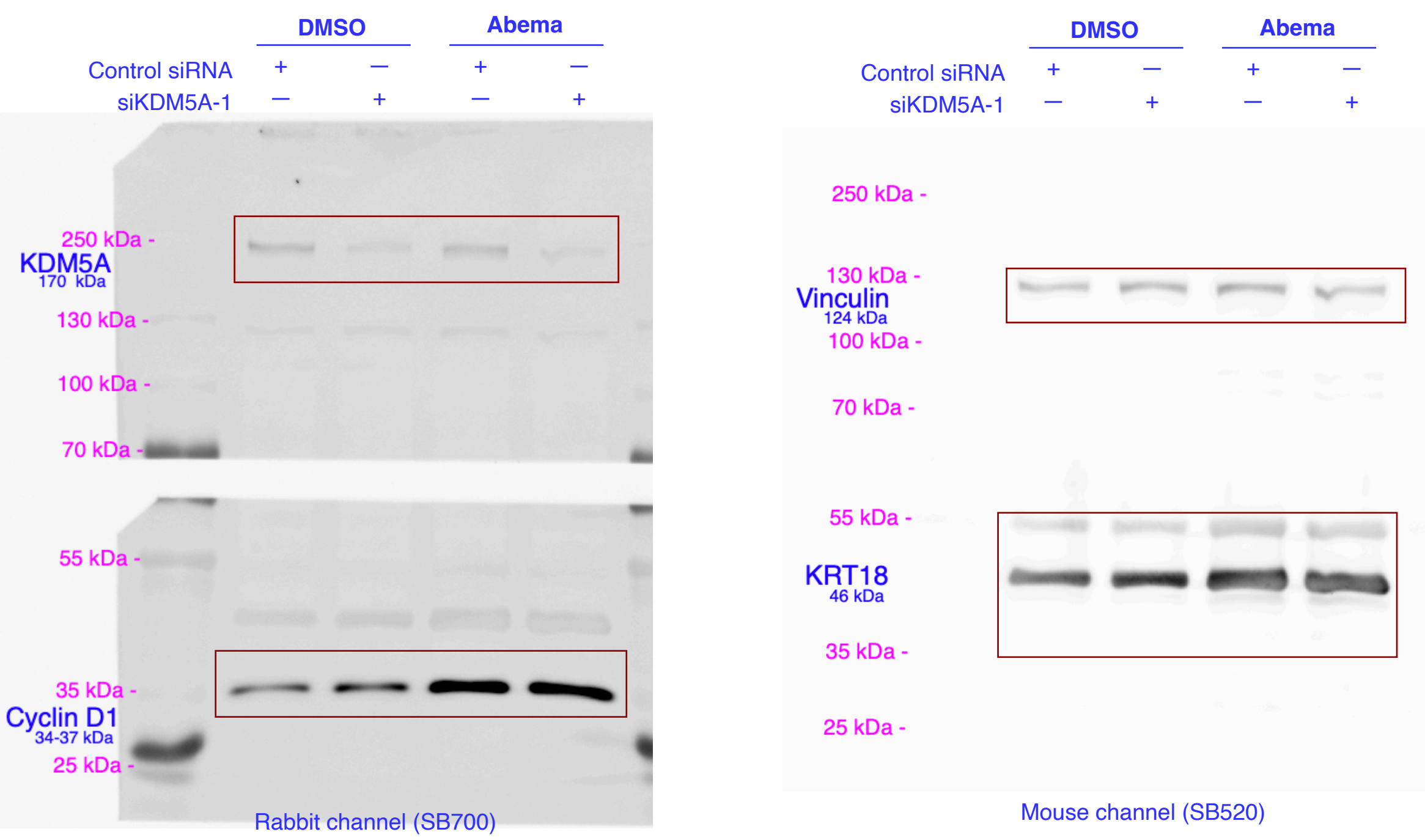

All 4 samples were loaded onto the same gel and normalised by equal total protein. KDM5A, cyclin D1, KRT18 and the loading control vinculin were immunoblotted on the same membrane.

MCF7 RB KO Sort Strategy

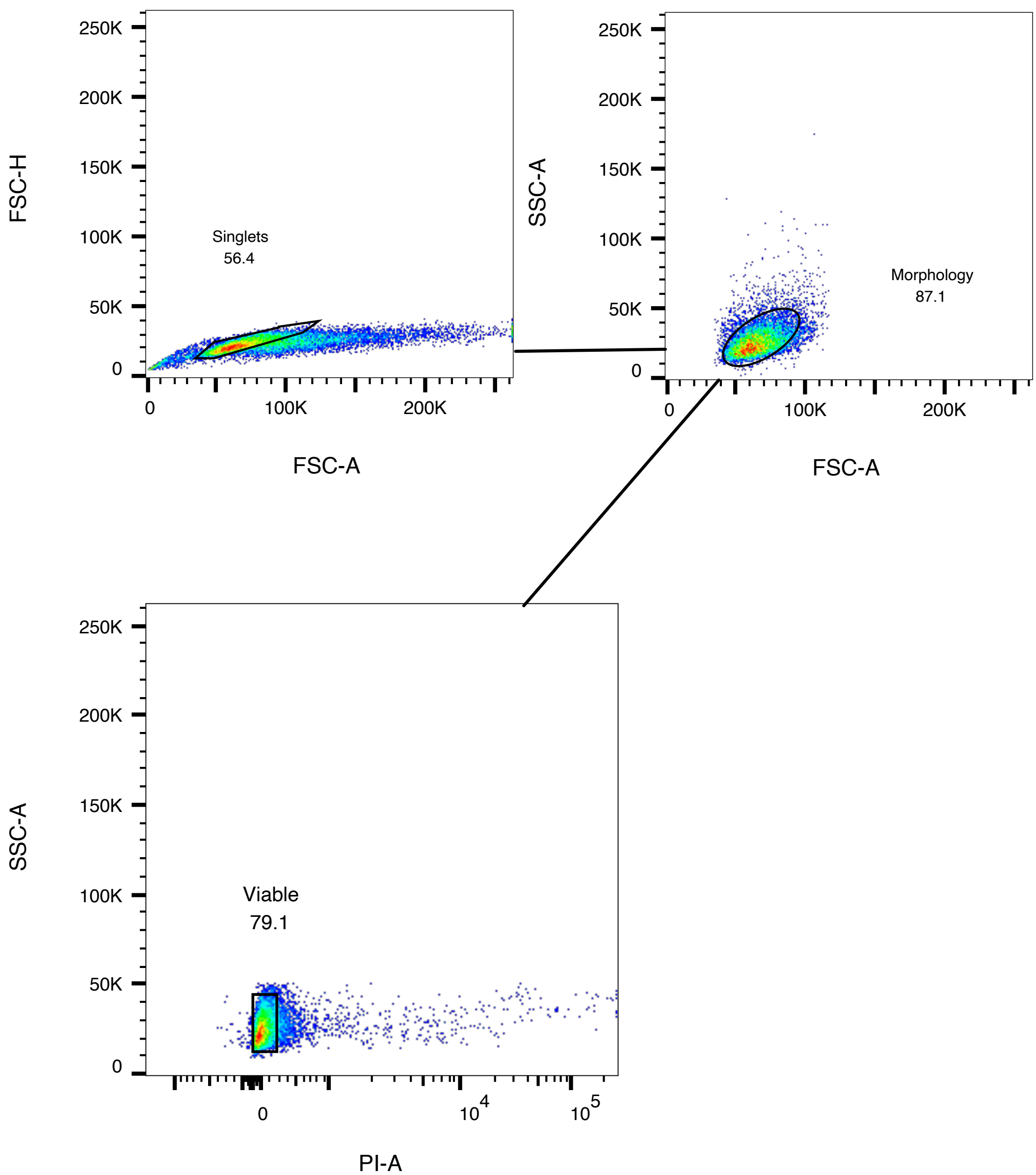

Supplementary Fig. 2 | Gating strategy for flow cytometric cell sorting for MCF7 single cells after CRISPR knock out for the generation of isogenic RB1 knockout cell lines.

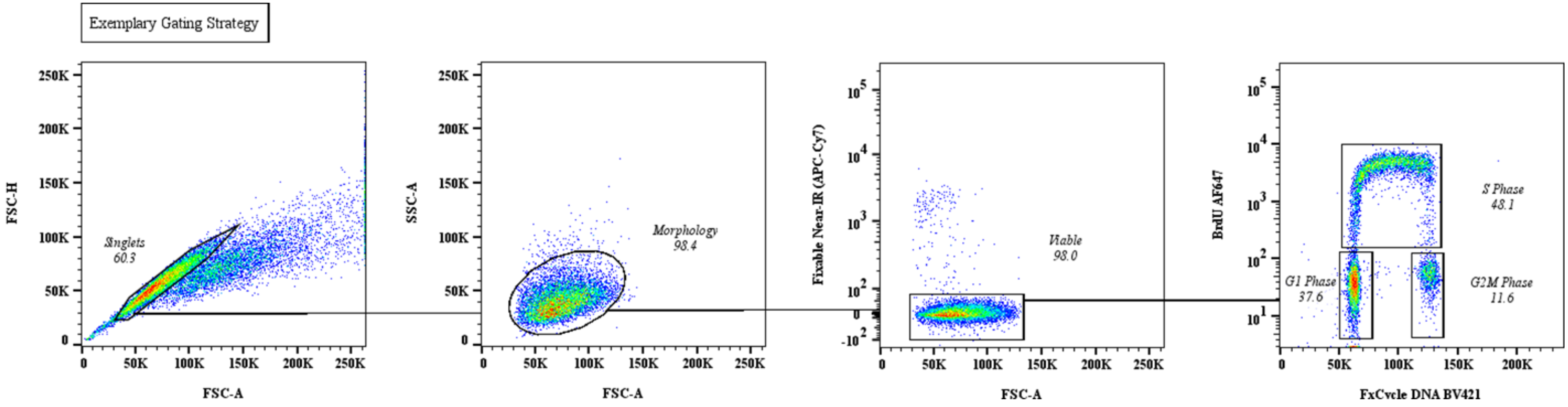

Supplementary Fig. 3 | Gating strategy for flow cytometric cell cycle analysis of tumour cells by BrdU incorporation and DNA content.

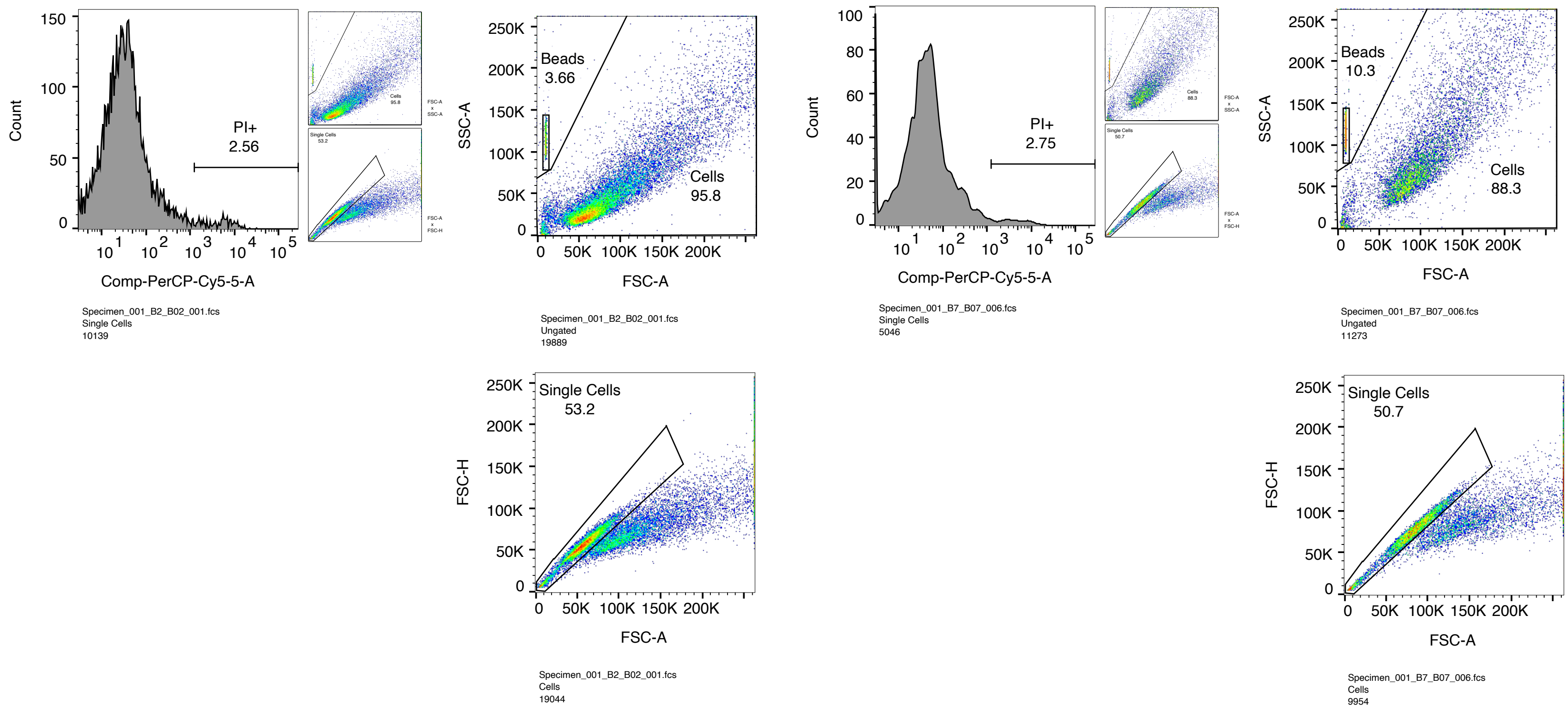

Supplementary Fig. 4 | Gating strategy for flow cytometric analysis of cell death defined by propidium iodide staining in tumour cells.
