# Supplementary material for: Rb-driven transcription limits its tumour-suppressive effects in breast cancer
Source: Nature. 2026 Aug 12;657(8131):517–24. doi: 10.1038/s41586-026-10886-w (PMC13558077; doi:10.1038/s41586-026-10886-w)
Supplement: Supplementary file 2 [file 41586_2026_10886_MOESM2_ESM.pdf]

nature portfolio

Corresponding author(s): Shom Goel

Last updated by author(s): Jun 27, 2026

Reporting Summary

Nature Portfolio wishes to improve the reproducibility of the work that we publish. This form provides structure for consistency and transparency in reporting. For further information on Nature Portfolio policies, see our Editorial Policies and the Editorial Policy Checklist.

Statistics

For all statistical analyses, confirm that the following items are present in the figure legend, table legend, main text, or Methods section.

n/a	Confirmed	
☐	☒	The exact sample size (*n*) for each experimental group/condition, given as a discrete number and unit of measurement
☐	☒	A statement on whether measurements were taken from distinct samples or whether the same sample was measured repeatedly
☐	☒	The statistical test(s) used AND whether they are one- or two-sided<br>*Only common tests should be described solely by name; describe more complex techniques in the Methods section.*
☒	☐	A description of all covariates tested
☐	☒	A description of any assumptions or corrections, such as tests of normality and adjustment for multiple comparisons
☐	☒	A full description of the statistical parameters including central tendency (e.g. means) or other basic estimates (e.g. regression coefficient) AND variation (e.g. standard deviation) or associated estimates of uncertainty (e.g. confidence intervals)
☐	☒	For null hypothesis testing, the test statistic (e.g. *F*, *t*, *r*) with confidence intervals, effect sizes, degrees of freedom and *P* value noted<br>*Give P values as exact values whenever suitable.*
☒	☐	For Bayesian analysis, information on the choice of priors and Markov chain Monte Carlo settings
☒	☐	For hierarchical and complex designs, identification of the appropriate level for tests and full reporting of outcomes
☒	☐	Estimates of effect sizes (e.g. Cohen's *d*, Pearson's *r*), indicating how they were calculated

Our web collection on statistics for biologists contains articles on many of the points above.

Software and code

Policy information about availability of computer code

Data collection	RNA-seq libraries were generated using the QuantSeq 3' mRNA-Seq Library Prep Kit FWD (Lexogen) and sequenced as single-end 100 bp reads on an Illumina NextSeq 2000 instrument. Cut&Run and DynaTag libraries were processed using paired-end sequencing on the same platform. Cell growth data were acquired using the Incucyte SX5 imaging system. Flow cytometry data were acquired on the BD A3 Symphony, LSR II flow cytometer, or BDFACS Aria Fusion 3 or 5.
Data analysis	RNA-seq data analysis:BBDuk (v38.90), STAR (v2.7.5b), XenofilteR (v1.6), Subread (v2.0.1), DESeq2 (v1.46.0), clusterProfiler (v4.14.6), msigdbr (v24.1.0), ComplexHeatmap (v2.22.0)<br>Microarray data analysis:oligo (v1.70.1), hugene21sttranscriptcluster.db (v8.8.0), affycoretools (v1.78.0), limma (v3.62.2)<br>Cut&Run and DynaTag data analysis:BBDuk (v38.90), Bowtie2 (v2.3.4.1), Picard (v3.0.0), samtools (v1.9), bedtools (v2.27.1), MACS2 (v2.2.7.1), DiffBind (v3.16.0), ChIPseeker (v1.42.0), HOMER (v4.11), ChIP-Enrich (v2.30.0), BETA (v1.0.7), clusterProfiler (v4.14.6), deepTools (v3.5.0)<br>H3K27ac Hi-ChIP: igraph (v2.1.1), ggraph (v2.2.1)<br>Mass Spectrometry Data Analysis: Spectronaut (v19.9), limma (v3.62.2)<br>Synergy analysis: SynergyFinder (v.3.14.0), ggplot2 (v.3.2.5)<br>Flow cytometry: BD FACSDiva software (v9.0) , FlowJo (v.10.8.1 and v.10.5.3)<br><br>Data were analysed using publicly available software. Custom code used for sequencing analyses and generation of figures and tables is available at GitHub (https://github.com/Goel-Laboratory/Rb-transcription-limits-tumour-suppression) and has been archived at Zenodo (doi:10.5281/zenodo.20789312).

For manuscripts utilizing custom algorithms or software that are central to the research but not yet described in published literature, software must be made available to editors and reviewers. We strongly encourage code deposition in a community repository (e.g. GitHub). See the Nature Portfolio guidelines for submitting code & software for further information.

Data

Policy information about availability of data

All manuscripts must include a data availability statement. This statement should provide the following information, where applicable:

- Accession codes, unique identifiers, or web links for publicly available datasets
- A description of any restrictions on data availability
- For clinical datasets or third party data, please ensure that the statement adheres to our policy

Sequencing datasets generated in this study, including raw and processed data, are available at the Gene Expression Omnibus (GEO) repository under SuperSeries accession GSE303576. Mass spectrometry proteomics data generated in this study have been deposited to the ProteomeXchange Consortium via the PRIDE66 repository under accession PXD066496. Previously published H3K27ac ChIP-seq and HiChIP datasets (GSE157222 and GSE157381 (ref.23)) used in this study are publicly available through GEO. Reference genomes hg38, mm10, and sacCer3 were downloaded from https://www.ncbi.nlm.nih.gov/assembly/GCA_000001405.15, http://hgdownload.cse.ucsc.edu/goldenPath/hg19/bigZips and https://ftp.ncbi.nlm.nih.gov/genomes/all/GCF/000/146/045/GCF_000146045.2_R64/GCF_000146045.2_R64_genomic.fna.gz, respectively. Gene expression data from the POP trial samples are publicly available at http://microarrays.curie.fr/publications/U981-GustaveRoussy/pop/.

Research involving human participants, their data, or biological material

Policy information about studies with human participants or human data. See also policy information about sex, gender (identity/presentation), and sexual orientation and race, ethnicity and racism.

Reporting on sex and gender	This manuscript includes an analysis of tumour samples from the previously completed POP trial (NCT02008734; Arnedos et al, Annals of Oncology 2018). This trial recruited 101 subjects with early-stage breast cancer. The overwhelming majority of these cancers occur in biological females, and eligibility was restricted to biological females to allow for study of tumour biology in this specific endocrine context.
Reporting on race, ethnicity, or other socially relevant groupings	Subjects of all ethnicities and races were eligible to participate in the POP trial. Data on race and ethnicity of subjects were not available at the time of preparing this manuscript. The study recruited subjects from France.
Population characteristics	The median age of subjects in the POP trial was 54 years (palbociclib group 54 years; control group 56 years). Forty-five percent of patients were pre-menopausal and 55 percent were post-menopausal. All patients had a diagnosis of breast cancer. Analysis for this manuscript was restricted to samples from subjects with hormone receptor-positive, HER2-negative breast cancer as these tumours are the subject of this study.
Recruitment	Eligible subjects were informed about their study by treating physicians at participating sites in France. All patients gave written informed consent prior to enrolment. Patients were required to have good ECOG performance status and adequate organ function, and hence patients with significant co-morbidity or frailty may have been excluded. Patients were randomised to receive palbociclib or or no treatment in a 3:1 fashion.
Ethics oversight	The study was conducted according to the principles of Good Clinical Practice and the Declaration of Helsinki; approved by an institutional review board (CSET 2013: 2016) and by a National Ethics Committee (Eudract 2013-002967-24, 25/09/2013).

Note that full information on the approval of the study protocol must also be provided in the manuscript.

Field-specific reporting

Please select the one below that is the best fit for your research. If you are not sure, read the appropriate sections before making your selection.

☒ Life sciences ☐ Behavioural & social sciences ☐ Ecological, evolutionary & environmental sciences

For a reference copy of the document with all sections, see nature.com/documents/nr-reporting-summary-flat.pdf

Life sciences study design

All studies must disclose on these points even when the disclosure is negative.

Sample size	No statistical methods were used to predetermine sample size. Sample sizes were chosen on the basis of established practice in the field, prior experience with the experimental systems (including responses to treatment), and the reproducibility of observed effects across independent biological replicates.
Data exclusions	One sample, abemaciclib IgG IP replicate 1, was omitted from the mass spectrometry analysis. Principle component analysis identified this sample as a clear outlier relative to the remaining biological replicates. All other samples were retained for analysis.
Replication	Replication was performed as appropriate for each experimental system. CUT&RUN profiling of Rb occupancy in MCF7 parental and RB1-knockout cells and ZR-75-1 cells following abemaciclib treatment was independently performed twice, each time with 1 to 2 biologically independent samples per condition. CUT&RUN profiling for Rb in MCF7 and ZR-75-1 cells following palbociclib and in OVCAR-3 and Kuramochi

cells following INX-315 was performed once, with two individual biological replicates per treatment arm. CUT&RUN profiling of KDM5A occupancy was independently repeated twice, and ER and H3K4me3 profiling were performed once. DynaTag Rb profiling in the PDX model was performed once, with each treatment arm consisting of tumour samples from two individual mice. Growth curves of the PDX model under palbociclib and fulvestrant treatment was repeated twice. RNA-seq of the treated tumours was performed once.

Immunoblot analyses of p-Rb and total Rb were performed once. Co-immunoprecipitation experiments were independently repeated twice for Rb and ER immunoprecipitations in MCF7 cells and once for KDM5A immunoprecipitation in MCF7 cells and Rb/KDM5A immunoprecipitation in ZR-75-1 cells. Rb immunoprecipitation mass spectrometry was performed using three biological replicates for Rb immunoprecipitation and three biological replicates for IgG controls. One abemaciclib-treated IgG immunoprecipitation replicate was excluded because principal component analysis identified it as an outlier relative to the remaining samples. All other data met quality-control criteria and were included in the analyses. Cell-cycle analyses were performed once for each cell line. Incucyte proliferation assays and drug-combination synergy experiments were independently repeated twice.

RNA-seq experiments in MCF7 cells treated with abemaciclib and fulvestrant were performed using three independent biological replicates. The DMSO and abemaciclib arms were independently repeated twice. RNA-seq experiments in ZR-75-1, CAMA-1, MDA-MB-453, MDA-MB-231 cells treated with abemaciclib and fulvestrant were performed once using three independent biological replicates. RNA-seq experiments in MCF7, ZR-75-1, and CAMA-1 cells treated with palbociclib were performed once using three independent biological replicates. RNA-seq experiments in MCF7 ESR1-PARENT and ESR1-Y537S cells treated with E2 or hormone-deprived media were performed twice using three independent biological replicates. RNA-seq in the same cells and treatments with the addition of CCND1 siRNA was performed once, with three biologically independent replicates. RNA-seq experiments in MCF7 cells with KDM5A siRNA knockdown were performed once using three independent biological replicates. KDM5A siRNA knockdown and western blotting for ER target genes was performed once but independent from the RNA-seq experiment. CRISPR-mediated deletion of the CCND1 enhancer was independently repeated twice, once for RT-qPCR and once for immunoblotting, whereas KRT18 enhancer deletion was performed once.

All independent replication attempts yielded results consistent with the conclusions presented.

Randomization

Prior to treatment initiation, aged-matched mice were randomized into groups of equal average tumor volume. Randomization is not relevant to other experiments for this study as cell-based assays require specific conditions depending on treatment arm

Blinding

In vivo experiments were performed in a blinded fashion when possible. Downstream analyses of mouse tissue (immunohistochemistry, image analysis) experiments were performed in a blinded fashion such that the people conducting and/or analyzing the assay were not aware of treatment groups until data gathering was complete. Blinding was not performed for in vitro experiments as the individual performing drug treatment of cells was also involved in collecting and labeling processed samples with details of treatment assessed.

Reporting for specific materials, systems and methods

We require information from authors about some types of materials, experimental systems and methods used in many studies. Here, indicate whether each material, system or method listed is relevant to your study. If you are not sure if a list item applies to your research, read the appropriate section before selecting a response.

Materials & experimental systems

n/a	Involved in the study	
☐	☒	Antibodies
☐	☒	Eukaryotic cell lines
☒	☐	Palaeontology and archaeology
☐	☒	Animals and other organisms
☐	☒	Clinical data
☒	☐	Dual use research of concern
☒	☐	Plants

Methods

n/a	Involved in the study	
☒	☐	ChIP-seq
☐	☒	Flow cytometry
☒	☐	MRI-based neuroimaging

Antibodies

Antibodies used

Flow cytometry:
BrdU antibody (clone 3D4, BioLegend, 560209; 1:20)

CUT&RUN and DynaTag:
Rb (clone 4H1; CST, 61121; 1 µg per reaction for cell lines, 1.5 µg for tumour tissue), ER (CST, 8644; 0.8 µg), H3K4me3 (CST, 9751; 1 µg), KDM5A (Abcam, ab194286; 1 µg), anti-mouse secondary antibody (Abcam, ab46540; 0.5 µg per reaction), mouse IgG2a (CST, 61656; 1 µg per reaction for cell lines, 1.5 µg for tumour tissue), and rabbit IgG (CST, 66362; 1 µg).

Western blot:
Primary antibodies used include Rb (BD 554136; 1:1,000), phospho-Rb S780 (CST, 8180; 1:1,000), phospho-Rb S807/811 (CST, 8516; 1:1,000), ER (CST, 8644; 1:1,000), KDM5A (Abcam ab194286; 1:5,000), Histone H3 (CST, 9715; 1:1,000), Cyclin D1 (CST, 55506; 1:1000), KRT18 (CST, 4548; 1:1000), TFF1 (CST, 15571; 1:1000), Cyclin A2 (Abcam, ab32386; 1:1000), actin (Sigma-Aldrich A3854; 1:10,000), and vinculin (Sigma V9131; 1:5,000). Secondary antibodies used include StarBright™ Blue 520 Goat Anti-Mouse IgG (Bio-rad, 12005867) and StarBright™ Blue 700 Goat Anti-Rabbit IgG (Bio-rad, 12004162), both at 1:2,500.

Immunohistochemistry:

	Ki-67 (Abcam, ab16667), phospho-Rb Ser807/811 (CST, Cat. 8516), and total-Rb (BD Biosciences Cat. 554136). ImmPRESS anti-Rabbit (Novus Cat. MP-7451-NB) or ImmPRESS anti-mouse (Novus Cat. MP-7452-NB).<br><br>Nuclear co-IP:<br>Rb (CST, 9039), ER (CST, 8644), mouse IgG2a (CST, 61656), and rabbit IgG (Abcam, ab37415)
Validation	Antibodies were validated for each species and usage as described on manufacturer's website, except:<br>Rb (clone 4H1; CST, 61121) antibody was validated for CUT&RUN using the isogenic MCF7 and ZR-75-1 Rb knockout cell lines. CUT&RUN signal was strongly reduced in RB1 knockout cells relative to parental controls. The ER antibody (CST 8644) was validated for CUT&RUN by the manufacturer in MCF7 human ER+ breast cancer cells. The KDM5A antibody (Abcam, ab194286) was validated for CUT&RUN in human breast cancer cells by multiple orthogonal approaches. First, KDM5A CUT&RUN peaks showed substantial concordance with publicly available KDM5A ChIP-seq datasets. Second, KDM5A CUT&RUN signal was reduced following siRNA-mediated depletion of KDM5A compared with non-targeting siRNA controls. These data are presented in the manuscript. The H3K4me3 antibody (CST 9751) was validated by the manufacturer in HCT 116 human colorectal carcinoma cells and generated enrichment patterns consistent with previously reported genomic distributions.<br><br>The BrdU antibody was used for detection of incorporated BrdU in accordance with the manufacturer's validated application specifications for flow cytometry. Specific staining was observed only in BrdU-labelled samples.<br><br>Antibodies against total Rb (BD Biosciences Cat. 554136), phospho-Rb (CST 8516, 8180), ER (CST 8644), KDM5A (Abcam, ab194286), cyclin D1 (CST 55506), KRT18 (CST 4548), TFF1 (CST, 15571), cyclin A2 (Abcam, ab32386), actin (Sigma-Aldrich A3854), vinculin (Sigma V9131) and other proteins analysed by immunoblotting and immunoprecipitation were used in these methods for which validation data were provided by the manufacturers. Antibody specificity was supported by the expected molecular weight of detected proteins and consistency with previously reported biological effects. For total Rb, specificity was also validated by CRISPR knockout of RB1 gene. For KDM5A and cyclin D1, immunoblotting specificities of these antibodies were further supported by siRNA-mediated knockdown, which resulted in a reduction in protein signal accompanied by reduced transcript abundance measured by RNA-seq.<br><br>Anti-Ki67 antibody [SP6] (Abcam, ab16667) was knockout validated by the manufacturer for IHC-P. The phospho-Rb (Ser807/811) antibody (CST 8516) and total Rb antibody (BD Biosciences 554136) were validated for IHC-P in human cells by their manufacturers. For immunohistochemistry analyses, antibody performance was additionally supported by staining patterns consistent with the known biology of the target proteins and expected treatment-associated changes in tumour proliferation and Rb pathway activity.

Eukaryotic cell lines

Policy information about cell lines and Sex and Gender in Research

Cell line source(s)	All cell lines were obtained from ATCC. Cell lines used include:<br>MCF7, ZR-75-1, CAMA-1, MDA-MB-453, MDA-MB-231, OVCAR-3, Kuramochi, and HEK293T.
Authentication	Cell line identity was confirmed by short tandem repeat analysis.
Mycoplasma contamination	All cell lines tested negative for mycoplasma by PCR.
Commonly misidentified lines<br>(See ICLAC register)	No commonly misidentified cell lines were used in this study.

Animals and other research organisms

Policy information about studies involving animals; ARRIVE guidelines recommended for reporting animal research, and Sex and Gender in Research

Laboratory animals	M. musculus, NOD.Cg-Prkdcscid Il2rgtm1Wjl (NSG), female, 6-10 weeks old
Wild animals	The study did not involve wild animals.
Reporting on sex	The overwhelming majority of ER+ breast cancers occur in biological females, therefore only female mice were used to allow for the study of tumour biology in this specific endocrine context.
Field-collected samples	The study did not involve samples collected from the field.
Ethics oversight	All mouse experiments were performed in compliance with federal laws and institutional guidelines as approved by the Animal Ethics Experimentation Committee of the Peter MacCallum Cancer Centre or the Garvan Institute of Medical Research Animal Ethics Committee.

Note that full information on the approval of the study protocol must also be provided in the manuscript.

Clinical data

Policy information about clinical studies

All manuscripts should comply with the ICMJE guidelines for publication of clinical research and a completed CONSORT checklist must be included with all submissions.

Clinical trial registration	*Provide the trial registration number from ClinicalTrials.gov or an equivalent agency.*
Study protocol	*Note where the full trial protocol can be accessed OR if not available, explain why.*
Data collection	*Describe the settings and locales of data collection, noting the time periods of recruitment and data collection.*
Outcomes	*Describe how you pre-defined primary and secondary outcome measures and how you assessed these measures.*

Plants

Seed stocks	*Report on the source of all seed stocks or other plant material used. If applicable, state the seed stock centre and catalogue number. If plant specimens were collected from the field, describe the collection location, date and sampling procedures.*
Novel plant genotypes	*Describe the methods by which all novel plant genotypes were produced. This includes those generated by transgenic approaches, gene editing, chemical/radiation-based mutagenesis and hybridization. For transgenic lines, describe the transformation method, the number of independent lines analyzed and the generation upon which experiments were performed. For gene-edited lines, describe the editor used, the endogenous sequence targeted for editing, the targeting guide RNA sequence (if applicable) and how the editor was applied.*
Authentication	*Describe any authentication procedures for each seed stock used or novel genotype generated. Describe any experiments used to assess the effect of a mutation and, where applicable, how potential secondary effects (e.g. second site T-DNA insertions, mosiacism, off-target gene editing) were examined.*

Flow Cytometry

Plots

Confirm that:

☒ The axis labels state the marker and fluorochrome used (e.g. CD4-FITC).

☒ The axis scales are clearly visible. Include numbers along axes only for bottom left plot of group (a 'group' is an analysis of identical markers).

☒ All plots are contour plots with outliers or pseudocolor plots.

☒ A numerical value for number of cells or percentage (with statistics) is provided.

Methodology

Sample preparation

Cell cycle analysis:
Thirty minutes prior to endpoint, 10 µmol/L BrdU (Sigma, B5002) was applied to the cells. Cells were stained using the Near-IR Fixable Viability Stain (Invitrogen, L34976) for 20 minutes at room temperature. Cells were fixed and permeabilised using FoxP3/Transcription Factor Staining Buffer Set (eBioscience, 00–5523–00). DNA was denatured using 2N HCl + 0.5% (v/v) Triton X-100 for 30 minutes. The acid was neutralised using 0.1 mol/L Na2B4O7.10H2O (pH 8.5) followed by 0.5% BSA in PBS. Cells were then stained with BrdU antibody (clone 3D4, BioLegend, 560209) for 1 hour. Prior to acquisition on the BD A3 Symphony, DNA was stained using FxCycle Violet Stain (Invitrogen, F10347). A minimum of 10,000 live cell events were recorded for each sample.

Cell death:
Media and PBS washes from each well were collected in a U-bottom 96-well plate to capture all floating/dead cells. Adherent cells were trypsinised and combined with floating cells in the media. Cells were centrifuged at 400 g for 4 min at 4C. All cells were washed with PBS twice. Cells were stained with 80 µL propidium iodide (PI) at 1:20 in 1X Binding Buffer on ice for 15 min. Cells were then analysed on the LSR II flow cytometer

Isogenic Rb knock out cell line sorting:
Viable bulk populations of edited cells defined by negative staining for propidium iodide at 1 ug/ml were single cell sorted by the BDFACS Aria Fusion 3 or 5 Flow Cytometer into each well of a 96-well plate.

Instrument

BD A3 Symphony, LSR II flow cytometer, BDFACS Aria Fusion 3 or 5

Software

FACSDiva software (BD Biosciences) was used to collect the data, and the data was analyzed using FlowJo (TreeStar).

Cell population abundance

5,000-10,000 cells were analysed for each sample.

Gating strategy

Cell cycle analysis:

Gating strategy

Debris and non-singlets were eliminated from analysis by single cell gates based on FSC-H and FSC-A. Nonviable cells were eliminated from gates on cells that stained negative for the Near-IR Fixable Viability Stain. Viable single cells were gated by BrdU and DNA content stained with FxCycle Violet stain, then gates for G1, S, and G2/M phases were applied to this population.

Cell death:
Debris and non-singlets were eliminated from analysis by single cell gates based on FSC-H and FSC-A. Gates for nonviable cells were applied to cells that stained positive for PI staining.

Isogenic Rb knockout cell line sorting:
Debris and non-singlets were eliminated from analysis by single cell gates based on FSC-H and FSC-A. For viable cells, gates were applied to cells that stained negative for PI.

☒ Tick this box to confirm that a figure exemplifying the gating strategy is provided in the Supplementary Information.
