# Supplementary material for: Rb-driven transcription limits its tumour-suppressive effects in breast cancer
Source: Nature. 2026 Aug 12;657(8131):517–24. doi: 10.1038/s41586-026-10886-w (PMC13558077; doi:10.1038/s41586-026-10886-w)
Supplement: Supplementary file 3 [file 41586_2026_10886_MOESM4_ESM.pdf]

nature portfolio

Peer Review File

Rb-driven transcription limits its tumour suppressive effects in breast cancer

Corresponding Author: Dr Shom Goel

Any redactions in this file are there to maintain patient confidentiality, the confidentiality of unpublished data, or to remove third-party material.

This file contains all reviewer reports in order by version, followed by all author rebuttals in order by version.

Version 0:

Reviewer comments:

Referee #1

(Remarks to the Author)
This paper focuses on a previously undescribed role for the tumor suppressor Rb in breast cancer cell lines. The authors, by means of cutting-edge techniques, highlight that inactive Rb is present in transcriptional hubs where it cooperates with the Estrogen Receptor (ER) to stimulate estrogen responsive genes. The authors provide evidence for this novel role of Rb, which is supported by the evidence of a physical interaction between ER and Rb, and delineate the fundamental role of the histone demethylase KDM5A as a key component of this complex.

The paper is original and novel as it highlights a role of inactive Rb that was previously unknown. The authors acknowledge prior studies focusing on the genomic role of inactive Rb as a modulator of different gene programs. However, this paper delineates for the first time that a direct link between Rb and estrogen signaling exists and has fundamental implications for the activity of CDK4/6 inhibitors.

The methodological approach is solid and the data are presented clearly. I have minor remarks as follows:
- The CUT&RUN data have been generated in MCF7 and T47D cells treated with abemaciclib for 2 days and 4 days, respectively. Extended data in figure 1 presents the same data with Palbociclib but the duration of treatment is not reported in the figure legend and is not clear in the Methods section. Please, clarify.
- The HiChIP data from a previous publication from the authors are also used in this paper. Going back to the original paper, these data were generated in MCF7 treated with abemaciclib for 7 days (vs 2 days in the CUT&RUN experiments presented here). This experimental discrepancy should be acknowledged and the potential bias introduced by the different duration of treatment in the two experiments should be discussed.
- Extended data Figure 4 I: a graphical problem shows blurred images in the curves; please, fix.

The conclusions drawn are mostly acceptable. In my opinion, the description of the role of Rb in stimulating Estrogen Response after short-term treatment with CDK4/6 inhibitors is very important to explain the synergism between endocrine therapy and CDK4/6 inhibitors, which is been known for decades, but never really explained experimentally. This is the real strength of the message of this manuscript. However, the Authors also make speculative conclusions about the implications of their discovery in the context of acquired resistance to endocrine therapy or to CDK4/6 inhibitors. These conclusions and speculations are not completely justified by the data. Please see the observations below:

- Lines 226-228. The Authors state “This persistent activation of ER targets impaired full suppression of E2F-target genes upon concomitant CDK4/6 inhibition and oestrogen deprivation, facilitating cell cycle escape and thus emergence of resistance (Fig. 3h and Extended Data Fig. 4i).” This is not proven by the data presented as no data on resistance are provided. Please clarify that this is a speculation. Please reword accordingly (for example… “potentially facilitating cell cycle escape …”)

- Lines 229-232 The Authors report: “These findings provide a mechanistic basis for how ESR1 mutations alone can drive resistance to combined CDK4/6 inhibitor and aromatase inhibitor therapy, explaining both their enrichment in resistant tumours and the clinical benefit of continuing CDK4/6 inhibition when switching to a selective oestrogen receptor degrader

in the setting of ESR1 mutation emergence." All these statements are not supported by the data presented. Firstly, these data presented are produced in CDK4/6i sensitive cells, treated for short time with CDK4/6 inhibitors. No data are provided in CDK4/6 resistant cells. Therefore, it is not clear why the data presented may "provide a mechanistic basis" for ESR1 mutations being involved in mechanisms of resistance to CDK4/6 inhibitors. Secondly, many other biologic explanations may be hypothesized for the enrichment of ESR1 mutations at resistance to CDK4/6 inhibitors and for the efficacy of switching to an oral SERD while ESR1 mutations emerge. These speculations should be heavily rephrased, or avoided altogether here.

- Discussion:
-lines 294-295: "The promoter and non-promoter sites around ER target genes contributing to this phenomenon are often unbound by Rb at baseline". Here should be reported, for clarity, that these data are not shown in the paper.

- lines 307-308: "On the other hand, in endocrine-resistant tumours – such as those harbouring ESR1 mutations – the same Rb-driven transcriptional program may sustain oncogenic expression, thereby limiting therapeutic efficacy." This hypothesis is not supported by the data presented. The data only suggest that the Rb-driven transcriptional program described here is still in place when an exogenous ESR1 allele is expressed in an otherwise ESR1 WT cell lines. I would not over-emphasize this piece of data and reduce the speculations on the potential role of inactive Rb in the setting of acquired resistance.
- lines 309-310: "This dynamic role has important implications for defining optimal treatment strategies after progression on CDK4/6 inhibitors." Again, not supported by the data. This is a speculation. It is not even clear what are the "important implications" here, since these implications are not explained. I would not refer to the resistance setting.

Suggested improvements:
- The volcano plot in figure 4A reports some genes of interest deriving from the IP-MS experiment. A full list of genes with significant interaction with Rb in this experiment should be reported as a Supplementary file. This is essential to ensure reprocucibility of this important experiment and will better support the focus on KDM5A in the final part of the manuscript.
- A better explanation of why the Authors have focused specifically on KDM5A among the other interactors should be provided for clarity.

Referee #2

(Remarks to the Author)
Summary of the key results

This is an interesting and well presented study examining the impact of pharmacological CDK 4/6 inhibition on activated Retinoblastoma protein localisation on chromatin. The authors use a relatively new and unbiased technique (CUT&RUN) to demonstrate Rb re-localising to multiple novel chromatin sites following abemaciclib/palbociclib treatment of ER+ BC cell lines. Comparable findings are also seen in a short window of opportunity study and two patient xenografts. They focus on non-promoter regions and uncover a novel induction of ER target genes, mediated by Rb dependant inhibition of KDM5A mediated ESR1 repression, resulting in increased tumour cell proliferation. The effects persist following CRISPR mediated ESR1 mutant generation in the cell lines used and could represent an escape mechanism for resistance to CDK4/6 inhibitors now widely used in early and advanced breast cancers.

Originality and significance: if not novel, please include reference

The authors build on their own prior work having undertaken and published a similar approach using ATAQseq and CHIPseq also in breast cancer cell lines (Watt A doi: 10.1038/s43018-020-00135-y). In their previous work they focused on enhancer regions and demonstrated comparable effects in ER positive and triple negative cell lines with CDK4/6i driving 'luminal differentiation and apoptotic evasion'. The novelty of the current work lies in their discovery that non-phosphorylated Rb itself acts to enhance transcriptional programmes promoting tumour cell growth. Specifically they demonstrate Rb represses KDM5A, already known to repress Estrogen Receptor function. They go on to confirm Rb mediated induction of ER responsive genes such as CCND1 which appears novel.

There is certainly some novelty in demonstration of a role of Rb as a transcriptional activator, specifically through the inhibition of KDM5A. However the induction of CCND1 has previously been shown and this has not been referenced (eg DOI: 10.1158/1535-7163.MCT-17-0290). This has also been shown in ER negative cell lines and it is not clear why the authors have limited this paper to 2 ER positive cell lines only. It is an intriguing thought that ER could potentially be derepressed in ER negative cell lines. I would like to see these data to enhance the novelty of the paper.

The significance in clinical translation even in ER+ breast cancer, is less well evidenced and could be strengthened. In ER expressing breast cancers CDK4/6 inhibitors are almost always administered with an estrogen targeting approach, either aromatase inhibition or a SERM/SERD. The authors demonstrate the the Rb induced activation of ER can be antagonised by ER targeting as expected. So how is this relevant to BC treatment? They hypothesise that the new found role of Rb "may even explain the capacity of CDK4/6 inhibition to restore endocrine sensitivity in resistant tumours." This should be investigated in this paper in my opinion. The in vivo and in vitro experiments in supplementary figure 4 could have been extended to look at resistance emergence with examination of the impact of Rb in this setting. This would significantly strengthen the paper and potentially provide extremely useful data for translational studies. The drug doses should be modified, however, to enhance applicability (see below).

Data & methodology: validity of approach, quality of data, quality of presentation

The concentrations of abemaciclib and palbociclib used in this manuscript are well above the published IC50s (eg DOI: 10.1158/1535-7163.MCT-17-0290) and at least twice the Cmax levels in human PK studies (therefore far above steady state levels). In vivo dosing is also high and this is evident from the growth curves presented in supplementary figure 4 b-g where synergy is less at the dose levels used in most of the studies and there is little additional benefit seen with combination vs single agent therapy. The translational relevance of the study would be significantly enhanced if drug concentrations were used that were achievable clinically. This is made all the more relevant with data demonstrating off target kinase inhibition, which differs between the CDK4/6 inhibitors, and may go some way to explain the differences in patterns of gene expression seen with abemaciclib and palbociclib in this study (eg figure 2a; reference that could be discussed doi. 10.1158?1541-7786.MCR-17-0468.). The off target effects (eg Wnt activation) may also impact on 'differentiation' (see below) and it would be useful to see the chromatin interaction hubs with palbociclib (figure 2c) and to see more discussion of this as a drawback.

Conclusions: robustness, validity, reliability

The interpretation that their findings in this paper "..the pharmacological activation of Rb.... reveals its role in enforcing lineage identity in tumour cells through enhancer-mediated gene activation" appears to draw more on their previous work. In this paper the term 'lineage' comes in the abstract and discussion only and one reference on B cells. There is no description of what is meant by this and no justification for the use of the term. Induction of ER in ER positive breast cancer cell lines can hardly be said to alter the lineage of those cell lines and the theory seems mainly to be a hangover from their 2021 paper that included both ER positive and negative cell lines (although did not look at ER transcription). As above, why not determine the impact on ER signalling in ER negative cells as previous studies have demonstrated increased CCND1 expression in multiple TNBC cell lines and xenografts (DOI: 10.1158/1535-7163.MCT-17-0290).

References: appropriate credit to previous work?
Some additional references and discussion suggested above

Clarity and context: lucidity of abstract/summary, appropriateness of abstract, introduction and conclusions.

Overall the paper is extremely well written and is very clear. The only exception is the link to lineage identity which is not explained at all and is not justified.

Referee #3

(Remarks to the Author)
This manuscript reports a novel function of the retinoblastoma (Rb) protein that activates a pro-proliferative gene program following treatment of breast cancer models and patients with CDK4/6 inhibitors. Rb is well known as a tumor suppressor that represses expression of cell cycle genes and is inactivated by CDK4/6 phosphorylation in breast and other cancers. While CDK4/6 inhibitors such as abemaciclib are used in the clinic, their efficacy is narrowly limited to certain cancer types, and they are used in combination therapy that also downregulates estrogen signaling in HR-positive breast cancer for reasons that are not completely understood. Here, using the Cut&Run approach for unbiased genomic screening, the authors identified a set of previously uncharacterized sites that are bound by Rb in response to abemaciclib treatment. These sites are outside of typical cell-cycle gene promoters and are correlated with signatures of gene activation. Remarkably, the authors link the presence of Rb at such gene enhancer regions to activation of several estrogen-responsive genes, including the gene encoding CycD1, which may explain why CDK4/6 inhibitors and anti-estrogen receptor (ER) therapies cooperate in certain breast cancer treatments. Finally, a mechanism for gene activation is supported in which Rb binds and inhibits the histone demethylase KDM5A.

Considering the great potential of using cell-cycle inhibitors as cancer therapies and the need to understand the response to CDK inhibitors in the tumor, the significance of this research is high. Several of the specific findings here are not novel, which may limit the appeal of the report to those interested in cancer biology and therapies. For example, that RB binds the genome at sites outside of cell-cycle genes, that RB antagonizes KDM5A to activate transcription, and that expression of CycD1 and other ER genes underlies the need for dual ER and CDK4/6 inhibition in therapy have been studied. On the other hand, the linking of these ideas in a mechanism that follows CDK4/6 inhibitor treatment will be an advance in our understanding of how RB pathway biology and these inhibitors impact each other. The genomic profiling experiments here are thorough, and there are several key experiments, including the RB and RB binding-site KO experiments, that provide strong support for the model. The overall experimental approach, which includes multiple model cell lines and patient samples, is for the most part robust. Additional details and supporting evidence for the proposed mechanism and its relevance are needed before publication is recommended. Several specific concerns are outlined below:

1) Tables of genes for Rb-up peaks at promoters and at nearby sites should be included. For example, lists of genes used for analysis in Fig. 1e and especially Fig. 2a should be provided.

2) Lines 129-136: The inclusion of the HiChIP data here adds little support to the overall model, and the conclusion is potentially misleading. The increased number of H3K27ac-marked noted and interconnections at hubs with nonpromoter Rb peaks reflects the chromatin structure at these locations but may be unrelated to the presence of Rb. Moreover, while H3K27ac may be correlated with transcriptional activity, there is lack of data here for transcriptional activity at those sites. Therefore, the conclusion that there is an “association between Rb’s integration into regulatory networks and the

transcriptional activity of those networks" is overstated from this analysis.
3) Line 150: How many out of the 3878 / 2240 RB non-promoter peaks are actually connected to genes upregulated by abemaciclib, i.e. how many genes are included in the analysis in Fig. 2a? What is the overlap in these genes between MCF-7 and ZR-75-1 cells?

4) Lines 139-142: How many genes with non-promoter Rb-up peaks induced by abemaciclib are estrogen response genes? Provide a table with gene names.

5) Lines 158-163: For the set of 405 non-promoter Rb-bound elements that looped to gene promoters (considered in Extended data Fig. 3F), it is not clear to what extent these genes are upregulated following abemaciclib treatment. This is only described for the 17 genes from the Hallmark Estrogen Response Early set. Also, how many of these 405 RB-bound elements identified in MCF7 cells are also bound by RB in ZR-75-1 cells and the xenograft model, and how many of these genes are upregulated in ZR-75-1 cells after CDK4/6 inhibition? The authors should provide tables naming the genes in the particular groups, and venn diagrams showing the overlap between the cellular models. Furthermore, the authors should clearly show whether the subgroup that contains the 17 estrogen-responsive genes that have enhancer-bound RB-looping looks comparable in the three cellular models.

The overall concern here (motivating points 3-5) is that data are being cherry-picked to support the authors' hypothesis, and more evidence is needed to support the generality of RB-activated gene expression that has meaningful consequences.

6) Line 168: Do KTR18 protein levels increase after abemaciclib treatment and does mutation of the enhancer prevent this potential increase? The authors should show a change in protein level (e.g. by Western blot) to demonstrate the significance of the change in mRNA level. Also, considering the importance in the overall model given to activation of CCND1, the authors should perform this experiment mutating the Rb-binding site impacting CCND1 expression, or they should provide an explanation of why it is technically not possible.

7) Protein levels of some genes should also be tested to support the mRNA expression studies presented in Figs. 2E-2H and in Extended Fig. 4H.

8) The experiment in Fig. 3C comparing the effects of Abema vs DMSO and Abema+fulv vs Abema on gene expression should also be done in the isogenic Rb KO lines to show Rb dependency of this result.

9) A table listing the interaction partners identified in the mass spectrometry screen presented in Fig. 4A should be included.

10) An immunoprecipitation experiment in an additional cell line should be performed to support the result in Fig. 4B.

11) Lines 253-257: The authors should demonstrate that knockdown of KDM5A or ER leads to loss of RB recruitment on enhancers. Although it is implied in the overall model, there is no evidence that either of the proteins recruits RB to the enhancers. Does ER recruit KDM5A or does KDM5A bind to DNA directly, as shown in (PMID: 18270511)? Overall, the concern here is that characterization of the complex is insufficient and that the model is insufficiently developed with respect to how the specificity of RB recruitment at these sites and in response to drug treatment is achieved.

12) Fig. 4g: This result needs to be supported by qPCR and Western blot for a few ER target genes, including CCND1. Also, the authors should report how many of the 405/17 genes described in lines 157-163 are upregulated in DMSO/siKDM5A-treated cells.

Referee #4

(Remarks to the Author)
I co-reviewed this manuscript with one of the reviewers who provided the listed reports.

Version 1:

Reviewer comments:

Referee #1

(Remarks to the Author)
I read the revised manuscript and the related supplementary material with interest. The paper has been significantly improved by this revision. I think this version includes all the requested changes I had previously made. Congratulations to the Authors for this important work.

Referee #2

(Remarks to the Author)
The authors have strengthened the manuscript in response to reviewers' comments.
However, I disagree with some of the interpretation of the response to reviewer 2 point 1 and the text and supplementary

figure 5b,c. The treatment with abemaciclib does induce an oestrogenic transcriptional programme but this is not inhibited by fulvestrant, perhaps unsurprisingly in the demonstrated absence of induction of the ESR1 transcript in an ER negative cell line. This needs greater clarity in the text as the implication from the new data is that the recruitment of Rb to the hubs is NOT ER dependent, at least not in its entirety. I note reviewer 3 (point 11) requested either KDM5A or ER knockdown an the authors undertook the former demonstrating no change in the Rb recruitment. I need to see the knockdown of ER to confirm whether the effect is entirely ER mediated, given the data from ER negative cell lines.
Otherwise no additional issues with the response

Referee #3

(Remarks to the Author)
The authors have sufficiently addressed a number of concerns, and we remain impressed with the overall novelty of the study, its significance, and the evidence supporting a number of conclusions. We have a couple of remaining points that should be addressed before publication:

1) The Venn diagrams provided to the reviewer should be included as Supplementary figures in the manuscript to increase transparency. This includes (1) the intersection of
significantly upregulated genes among MCF-7 and ZR-75-1 within the set of non-promoter Rb up-peaks (shown on page 16 of the reviewer response); and (2) the intersection of significantly upregulated genes among MCF-7, ZR-75-1, and CAMA-1 cells within the set of genes that form loops with Rb non-promoter up-peaks (shown on page 18 of the reviewer response). Here the authors should also clarify that the set of 18 genes that is the intersection of all three gene sets includes the 17 estrogen response genes highlighted in the manuscript.

2) Based on the minimal differences observed in the Rb-KDM5a IP with addition of abemaciclib in the new Extended Data Fig 7a, the authors should at least acknowledge in the main text the alternative interpretation that this effect could be a specific feature observed in MCF7 cells.

Referee #4

(Remarks to the Author)
I co-reviewed this manuscript with one of the reviewers who provided the listed reports.

Version 2:

Reviewer comments:

Referee #2

(Remarks to the Author)
The authors make a very cogent argument regarding the request to demonstrate ER knockdown and I concur that this would not add sufficiently to the manuscript to justify the additional experimental work. I have no further comments and congratulate the authors on their excellent body of work.

Open Access This Peer Review File is licensed under a Creative Commons Attribution 4.0 International License, which permits use, sharing, adaptation, distribution and reproduction in any medium or format, as long as you give appropriate credit to the original author(s) and the source, provide a link to the Creative Commons license, and indicate if changes were made.
In cases where reviewers are anonymous, credit should be given to 'Anonymous Referee' and the source.
The images or other third party material in this Peer Review File are included in the article's Creative Commons license, unless indicated otherwise in a credit line to the material. If material is not included in the article's Creative Commons license and your intended use is not permitted by statutory regulation or exceeds the permitted use, you will need to obtain permission directly from the copyright holder.
To view a copy of this license, visit https://creativecommons.org/licenses/by/4.0/

Response to Referees

Manuscript: Watt, Ahn et al. Rb-Driven Transcriptional Programs Counteract Its Tumour Suppressor Function in Luminal Breast Cancer (Manuscript ID: 2025-08-22316A).

We thank the reviewers for their constructive comments to help strengthen our manuscript. We have worked diligently to address reviewer questions and concerns by providing new data and revising the text as appropriate. Below, please find the original reviewer comments in bold text and our responses indicated in normal type face. In each case, we provide the new data within this document and indicate how the manuscript has been revised to incorporate these data.

Response to Referee #1

We thank the reviewer for their detailed reading of our manuscript and thoughtful comments. We include below a point-by-point response to the comments and indicate where new data has been added to our manuscript to strengthen our findings.

Reviewer summary: This paper focuses on a previously undescribed role for the tumor suppressor Rb in breast cancer cell lines. The authors, by means of cutting-edge techniques, highlight that inactive Rb is present in transcriptional hubs where it cooperates with the Estrogen Receptor (ER) to stimulate estrogen responsive genes. The authors provide evidence for this novel role of Rb, which is supported by the evidence of a physical interaction between ER and Rb, and delineate the fundamental role of the histone demethylase KDM5A as a key component of this complex.

The paper is original and novel as it highlights a role of inactive Rb that was previously unknown. The authors acknowledge prior studies focusing on the genomic role of inactive Rb as a modulator of different gene programs. However, this paper delineates for the first time that a direct link between Rb and estrogen signaling exists and has fundamental implications for the activity of CDK4/6 inhibitors. The methodological approach is solid and the data are presented clearly.

We thank the reviewer for this thoughtful summary of our work and for recognising the novelty of the findings. We appreciate the reviewer's clear articulation of the central advance of the study - namely the identification of a previously unrecognised role for Rb in estrogen receptor-driven transcriptional regulation. We are also grateful for the reviewer's acknowledgement of the conceptual implications of this mechanism for understanding the activity of CDK4/6 inhibitors in breast cancer. Finally, we appreciate the positive assessment of the methodological approach and clarity of the data presentation.

1) **The CUT&RUN data have been generated in MCF7 and T47D cells treated with abemaciclib for 2 days and 4 days, respectively. Extended data in figure 1 presents the same data with Palbociclib but the duration of treatment is not reported in the figure legend and is not clear in the Methods section. Please, clarify.**

   We thank the reviewer for pointing this out. Palbociclib treatment of MCF7 and ZR-75-1 cells was for 2 and 4 days, respectively – the same duration as was used for our abemaciclib experiments. We have updated the Methods section of our manuscript to include this information (lines 440-442). Importantly, our data demonstrate consistent patterns of Rb chromatin binding in abemaciclib- and palbociclib-treated cells.

2) **The HiChIP data from a previous publication from the authors are also used in this paper. Going back to the original paper, these data were generated in MCF7 treated with abemaciclib for 7 days (vs 2 days in the CUT&RUN experiments presented here). This**

experimental discrepancy should be acknowledged and the potential bias introduced by the different duration of treatment in the two experiments should be discussed.

The reviewer is correct that the CUT&RUN (2 days) and H3K27ac HiChIP (7 days) experiments were performed at different treatment durations. As noted, this discrepancy represents a limitation of the integrative analysis, as transient chromatin interactions present at earlier time points may not be fully captured in the 7-day HiChIP dataset. We have now explicitly acknowledged this difference in treatment duration and discussed the potential limitations of integrating these datasets in the revised manuscript (lines 134-137).

Despite the difference in timepoints, our experimental observations support the relevance of the HiChIP data for interpreting the Rb CUT&RUN results. Specifically, we observe increased H3K27ac signal at Rb-bound regulatory regions as early as 2 days after treatment initiation, indicating that activation of these enhancers is already underway at the timepoint when CUT&RUN profiling was performed. This suggests that the enhancer-promoter interactions captured in the 7-day HiChIP dataset likely reflect regulatory relationships that are already emerging at earlier stages of CDK4/6 inhibition.

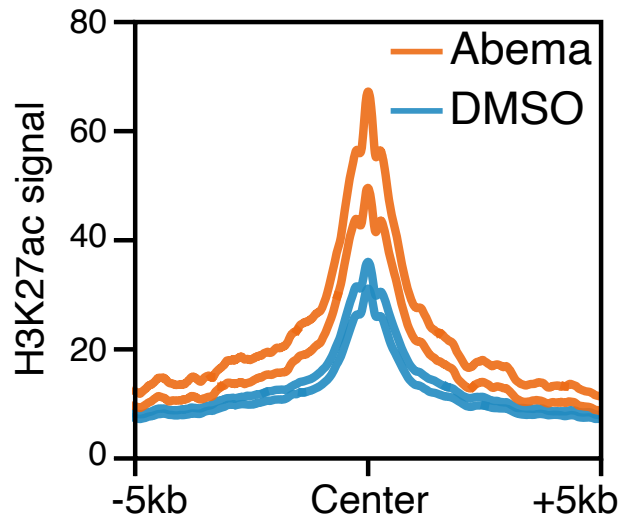

Composite H3K27ac signal intensity at Rb non-promoter up peak regions in MCF7 cells treated with DMSO or abemaciclib 500 nM for two days.

(Data included here for reviewer's reference).

3) **Extended data Figure 4I: a graphical problem shows blurred images in the curves; please, fix.**

The reviewer is correct that the curves in Extended Data Fig. 4i were difficult to visualise due to a graphical issue. We have corrected this problem in the revised figures.

Furthermore, we have repeated and expanded this experiment to improve the robustness of the analysis. The growth curves are now displayed in separate plots for parental and *ESR1*-Y537S cells, which allows clearer visualisation of the trajectories. We have also extended the experiment to include treatment arms incorporating selective oestrogen receptor degraders (SERDs). These updated data are now presented in Extended Data Fig. 6e.

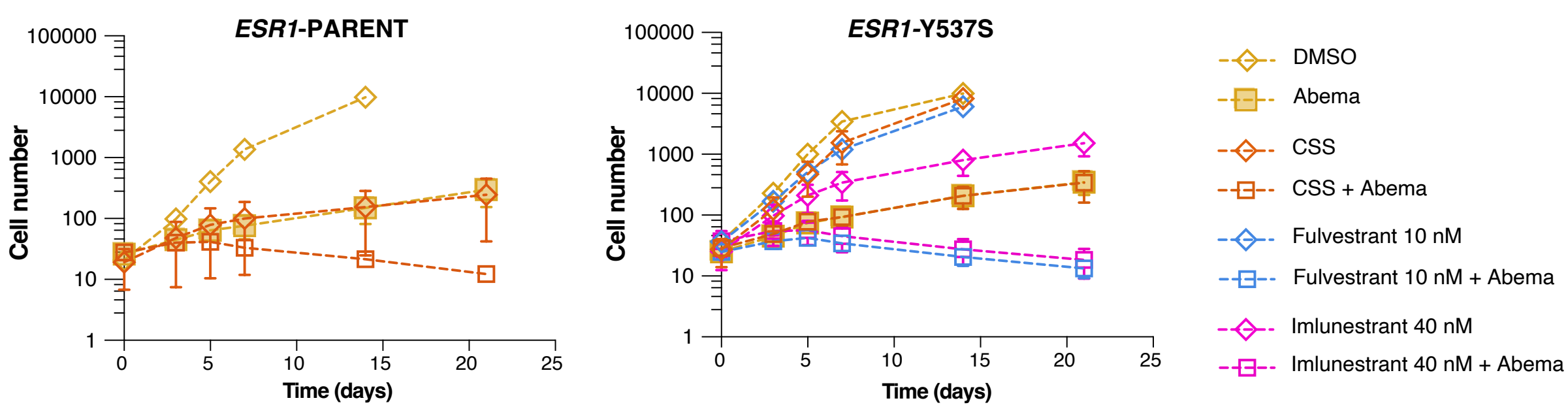

Cell number of MCF7 *ESR1*-Parent or *ESR1*-Y537S mutant cells expressing H2B-GFP grown in complete medium treated with DMSO, abemaciclib, fulvestrant, or imlunestrant, or in charcoal stripped serum (CSS) treated with DMSO or abemaciclib, tracked over 21 days (n = 4 separate wells per group; Extended Data Fig. 6e in revised manuscript).

These data confirm that *ESR1*-Y537S cells are resistant to oestrogen deprivation (charcoal stripped serum, CSS) and exhibit outgrowth under abemaciclib monotherapy as well as under combined abemaciclib treatment + oestrogen deprivation. In contrast, combined CDK4/6 inhibition with ER blockade using SERDs, which can target the mutant ER, prevents cancer cell outgrowth.

4) **Lines 226-228. The Authors state "This persistent activation of ER targets impaired full suppression of E2F-target genes upon concomitant CDK4/6 inhibition and oestrogen deprivation, facilitating cell cycle escape and thus emergence of resistance (Fig. 3h and Extended Data Fig. 4i)." This is not proven by the data presented as no data on resistance are provided. Please clarify that this is a speculation. Please reword accordingly (for example… "potentially facilitating cell cycle escape …")**

We agree with the reviewer that the original wording may have overstated what is directly demonstrated by the data in Fig. 3h. The relevant text in the manuscript has been revised to reflect this more cautious interpretation (lines 241-244).

We also note, however, that a <u>functional resistance</u> phenotype is observed in Extended Data Fig. 6e (previously ED Fig. 4i), where *ESR1*-Y537S cells exhibit sustained outgrowth over three weeks under combined hormone deprivation (charcoal-stripped serum; CSS) and abemaciclib treatment, whereas wild-type cells are fully suppressed under the same conditions. Thus, *ESR1*-mutant cells display resistance to this combination therapy in our model system. That said, we do agree that the E2F target gene expression data shown in Fig. 3h (of the original manuscript) alone do not establish resistance. Rather, these data indicate incomplete repression of E2F targets in *ESR1*-mutant cells, which we interpret as being consistent with the resistant phenotype observed in the proliferation assays.

To further clarify the mechanism underlying this observation, we have now added additional data. First, we show that switching *ESR1*-Y537S cells from CSS + abemaciclib to **fulvestrant + abemaciclib** restores repression of E2F target genes (measured by RNA-seq) and suppresses tumour cell outgrowth in our proliferation assays (Extended Data Fig. 6d and 6e). These findings indicate that persistent Rb-mediated ER signalling in *ESR1*-mutant cells can limit the extent of cell cycle repression achieved with CDK4/6 inhibition plus oestrogen deprivation, and that pharmacologic ER degradation can overcome this resistance phenotype.

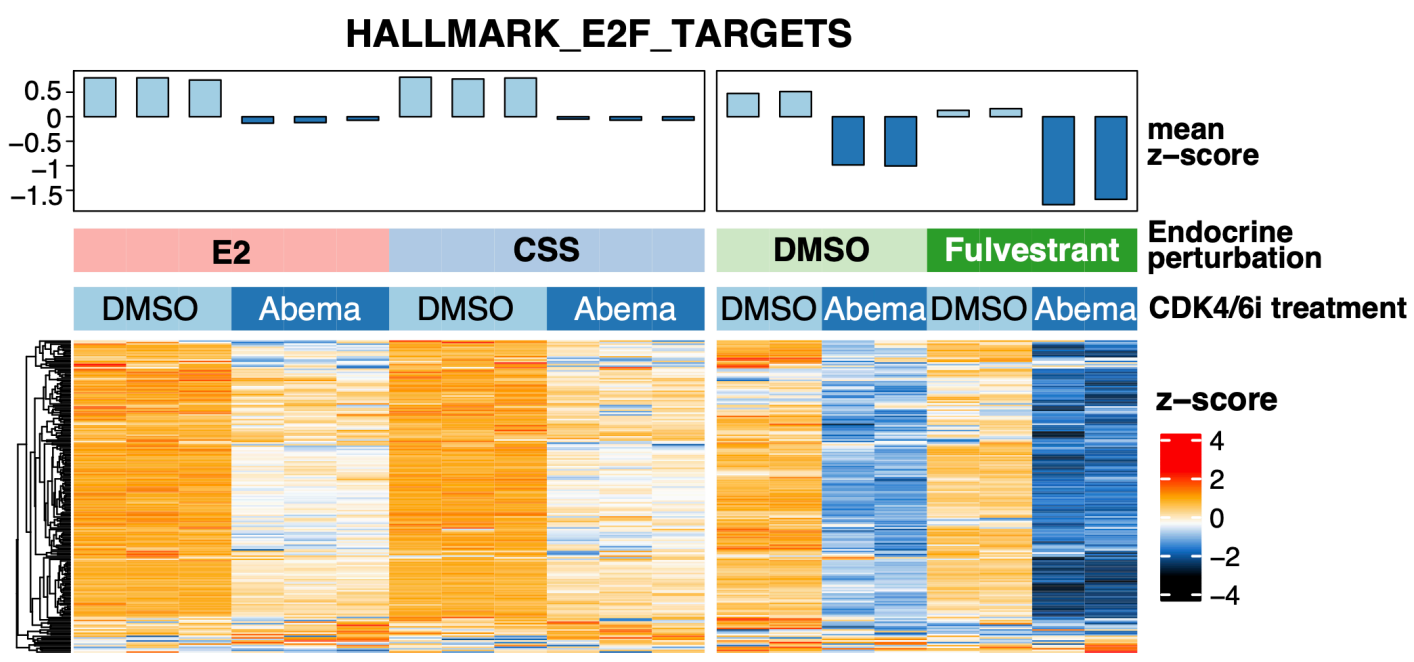

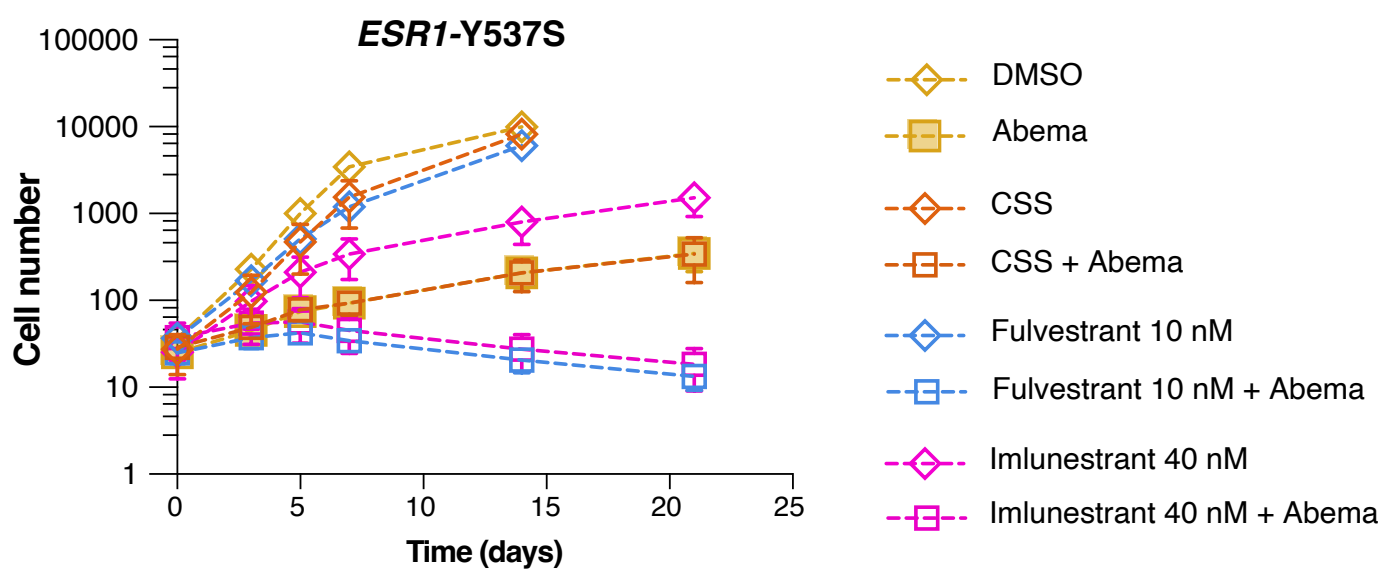

Top: MCF7 *ESR1*-Y537 cells were treated with drugs indicated for 5 days and RNA-seq was performed. Heatmap show expression of E2F target genes. Note that estrogen deprivation (CSS) does not deepen suppression of E2F targets when added to abemaciclib, but fulvestrant (a SERD) does (Extended Data Fig. 6d in revised manuscript).

Bottom: MCF7 *ESR1*-Y537S mutant cell counts tracked over 21 days (Day 0 is time of treatment initiation) ($n = 4$ separate wells per group; ED Fig. 6e in revised manuscript). (CSS – charcoal stripped serum).

Second, we examined the specific role of *CCND1*, which is induced by CDK4/6 inhibition in an ER-dependent manner. Knockdown of *CCND1* in *ESR1*-Y537S cells treated with CSS + abemaciclib resulted in deeper suppression of E2F target genes compared with CSS + abemaciclib alone (Fig. 3f). Together, these results support a model in which persistent ER activity - and specifically a failure to downregulate of its downstream target *CCND1* - can undermine full cell cycle repression during combination therapy.

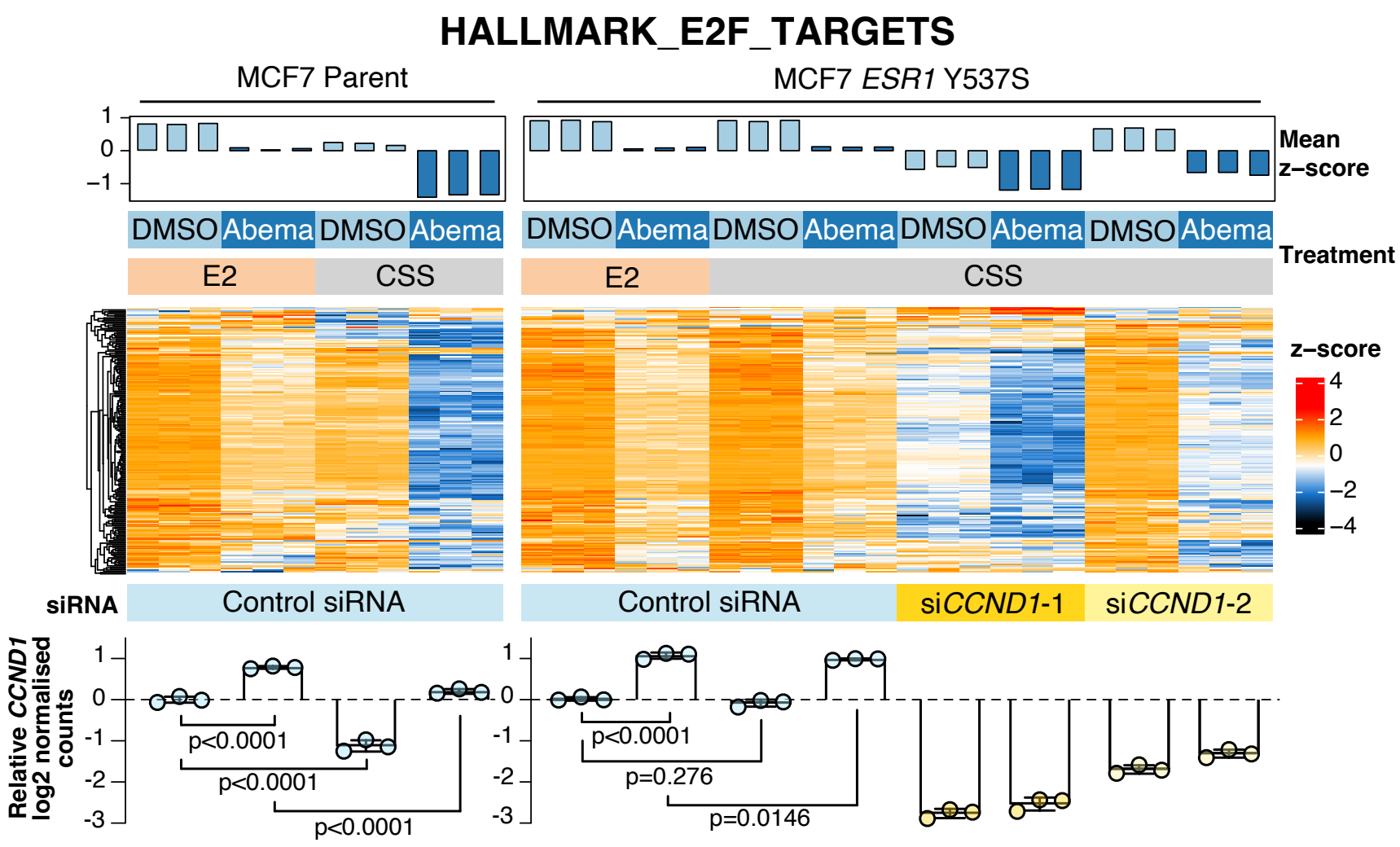

MCF7 cells (parent or *ESR1*-Y537S as indicated) were treated with drugs shown for 5 days, with or without concomitant knockdown of *CCND1,* and RNA-seq was performed. Heatmap shows expression of genes from the Hallmark E2F Target gene set. Bar plot below shows extent of *CCND1* knockdown with each siRNA used (Fig. 3f in revised manuscript).

5) **Lines 229-232 The Authors report: "These findings provide a mechanistic basis for how ESR1 mutations alone can drive resistance to combined CDK4/6 inhibitor and aromatase inhibitor therapy, explaining both their enrichment in resistant tumours and the clinical benefit of continuing CDK4/6 inhibition when switching to a selective oestrogen receptor degrader in the setting of ESR1 mutation emergence." All these statements are not supported by the data presented. Firstly, these data presented are produced in CDK4/6i sensitive cells, treated for short time with CDK4/6 inhibitors. No data are provided in CDK4/6 resistant cells. Therefore, it is not clear why the data presented may "provide a mechanistic basis" for ESR1 mutations being involved in mechanisms of resistance to CDK4/6 inhibitors. Secondly, many other biologic explanations may be hypothesized for the enrichment of ESR1 mutations at resistance to CDK4/6 inhibitors and for the efficacy of switching to an oral SERD while ESR1 mutations emerge. These speculations should be heavily rephrased, or avoided altogether here.**

The reviewer is correct that the original wording overstated the scope of our conclusions. We have therefore revised the relevant text throughout the manuscript to better reflect what is directly supported by our data (e.g. lines 249-254).

Our intention was not to imply that we directly model CDK4/6 inhibitor-resistant tumours or that our study fully explains the clinical enrichment of *ESR1* mutations. We also note that *ESR1*-mutant cells remain sensitive to single-agent CDK4/6 inhibition in short-term assays. Rather, our data demonstrate the following: (i) pharmacologically activated Rb can drive ER signalling in *ESR1*-mutant cells, including induction of ER target genes such as *CCND1* (Fig. 3e and 3f); (ii) persistence of this ER-driven transcription in the setting of oestrogen deprivation impairs complete suppression of E2F target genes during combined CDK4/6 inhibition and hormone deprivation (Fig. 3f); (iii)

this incomplete E2F repression is associated with tumour cell proliferation in long-term assays (Extended Fig. 6e); and (iv) suppression of mutant ER signalling - either pharmacologically with a SERD or genetically through *CCND1* knockdown - restores E2F target repression and sustained control of cell proliferation (Fig. 3f; Extended Data Fig. 6d and 6e).

Together, these findings support a model in which constitutive ER activity can undermine complete cell cycle control during combination therapy. Importantly, the ER-driven transcriptional program that contributes to this effect is itself promoted by pharmacologic activation of the tumour suppressor Rb, which represents the central conceptual advance of our study.

We agree with the reviewer that additional biological mechanisms likely contribute to the clinical enrichment of *ESR1* mutations and to the observed benefit of switching to SERD-based therapy. The manuscript has therefore been revised to clarify that our findings provide a mechanistic framework consistent with these clinical observations, rather than a definitive explanation.

6) **lines 294-295: "The promoter and non-promoter sites around ER target genes contributing to this phenomenon are often unbound by Rb at baseline". Here should be reported, for clarity, that these data are not shown in the paper.**

We thank the reviewer for pointing this out. To support this statement, we have now included a figure to demonstrate the point (Extended Data Fig. 3g).

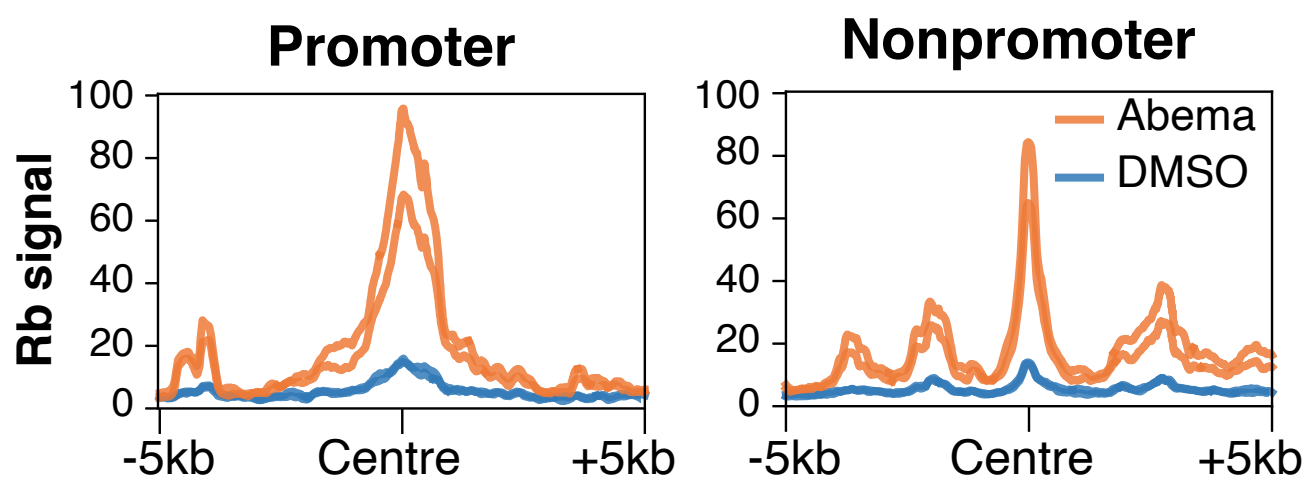

Composite Rb signal intensity at promoter (left) and non-promoter (right) up peak regions at or near genes in the *Hallmark Estrogen Response Early* geneset identified in the ORA from Fig. 2a. Rb signal obtained from CUT&RUN in MCF7 cells treated with DMSO or abemaciclib 500 nM for two days. This panel is included in the updated manuscript in Extended Data Fig. 3g.

7) **lines 307-308: "On the other hand, in endocrine-resistant tumours – such as those harbouring ESR1 mutations – the same Rb-driven transcriptional program may sustain oncogenic expression, thereby limiting therapeutic efficacy." This hypothesis is not supported by the data presented. The data only suggest that the Rb-driven transcriptional program described here is still in place when an exogenous ESR1 allele is expressed in an otherwise ESR1 WT cell lines. I would not over-emphasize this piece of data and reduce the speculations on the potential role of inactive Rb in the setting of acquired resistance.**

The reviewer raises an important point regarding the interpretation of these data. We would like to clarify that the *ESR1*-Y537S mutation used in our study was introduced into the endogenous *ESR1* locus in MCF7 cells and is expressed under native regulatory control. Thus, this model reflects ligand-independent ER signalling at physiological expression levels, rather than overexpression of an exogenous *ESR1* allele.

We agree that our system does not directly model acquired endocrine-resistant tumours from patients. Rather, our data do demonstrate that ligand-independent ER activity can sustain Rb-associated transcriptional programmes and limit full suppression of E2F target genes under conditions of oestrogen deprivation and CDK4/6 inhibition in this controlled setting. The manuscript has been revised to clarify that these findings are consistent with, but do not directly establish, a role for this mechanism in clinical resistance (lines 327-329).

8) **lines 309-310: “This dynamic role has important implications for defining optimal treatment strategies after progression on CDK4/6 inhibitors.” Again, not supported by the data. This is a speculation. It is not even clear what are the “important implications” here, since these implications are not explained. I would not refer to the resistance setting.**

The reviewer is correct that the original wording was overly broad and not sufficiently supported by the data. We have therefore revised this statement to more precisely reflect the scope of our findings (lines 329-330). Our data do not define optimal treatment strategies after progression on CDK4/6 inhibitors. Rather, they demonstrate that persistent ER signalling can limit complete cell cycle repression under conditions of CDK4/6 inhibition and oestrogen deprivation, and that pharmacologic ER degradation can restore this effect in our model system.

9) **The volcano plot in figure 4A reports some genes of interest deriving from the IP-MS experiment. A full list of genes with significant interaction with Rb in this experiment should be reported as a Supplementary file. This is essential to ensure reproducibility of this important experiment and will better support the focus on KDM5A in the final part of the manuscript.**

We thank the reviewer for this constructive suggestion. We agree that providing the complete list of significantly enriched Rb-interacting proteins from the IP-MS experiment will improve transparency and reproducibility and further contextualize our focus on KDM5A. We have now included a Supplementary Table (Supplementary Table 6) containing the full list of detected proteins, along with associated statistical parameters (e.g., $\log_2$ fold-change, adjusted *p*-values, peptide counts, and replicate information). This dataset includes all significantly enriched interactors identified in the Rb IP relative to IgG IP, and the number of proteins identified is consistent with other published nuclear IP-MS experiments. Given that several classical Rb interactors were identified, this number likely reflects the complexity of nuclear Rb complexes and the depth of the mass spectrometry, rather than a non-specific pull-down.

10) **A better explanation of why the Authors have focused specifically on KDM5A among the other interactors should be provided for clarity.**

We thank the reviewer for this important point. We agree that clearer justification for prioritising KDM5A among the identified Rb interactors is warranted. The IP–MS experiment was performed in an unbiased manner, and several significantly enriched Rb-associated proteins were identified. To prioritize candidates for functional follow-up, we applied the following biologically motivated criteria:

1. Established roles in ER signalling or regulation of ER-dependent transcription.
2. Function as a chromatin modifier with the potential to directly influence enhancer/promoter activity.

KDM5A satisfied these criteria. Among the significantly enriched hits, only a limited subset fulfilled this combination of criteria. For example, another “hit” that we evaluated was KMT2C, which has previously been reported to increase ER activity at enhancers (Gala, Li et al. 2018). However, *KMT2C* knockdown did not impact the expression of ER target genes in response to CDK4/6 inhibition. We have revised the manuscript to clarify this prioritization strategy (lines 265-266).

<u>Response to Referee #2</u>

We thank the reviewer for their detailed reading of our manuscript and helpful comments. We include below a point-by-point response to the comments and indicate where new data has been added to our manuscript to strengthen our findings.

Reviewer summary: This is an interesting and well presented study examining the impact of pharmacological CDK 4/6 inhibition on activated Retinoblastoma protein localisation on chromatin. The authors use a relatively new and unbiased technique (CUT&RUN) to demonstrate Rb re-localising to multiple novel chromatin sites following abemaciclib/palbociclib treatment of ER+ BC cell lines. Comparable findings are also seen in a short window of opportunity study and two patient xenografts. They focus on non-promoter regions and uncover a novel induction of ER target genes, mediated by Rb dependant inhibition of KDM5A mediated ESR1 repression, resulting in increased tumour cell proliferation. The effects persist following CRISPR mediated ESR1 mutant generation in the cell lines used and could represent an escape mechanism for resistance to CDK4/6 inhibitors now widely used in early and advanced breast cancers.

The authors build on their own prior work having undertaken and published a similar approach using ATACseq and CHIPseq also in breast cancer cell lines (Watt A doi: 10.1038/s43018-020-00135-y). In their previous work they focused on enhancer regions and demonstrated comparable effects in ER positive and triple negative cell lines with CDK4/6i driving 'luminal differentiation and apoptotic evasion'. The novelty of the current work lies in their discovery that non-phosphorylated Rb itself acts to enhance transcriptional programmes promoting tumour cell growth. Specifically they demonstrate Rb represses KDM5A, already known to repress Estrogen Receptor function. They go on to confirm Rb mediated induction of ER responsive genes such as CCND1 which appears novel.

We thank the reviewer for this thoughtful and balanced summary of our work. We appreciate their recognition of the conceptual advance in identifying a gene-activating role for Rb and the integration of chromatin profiling with functional and in vivo models. We are encouraged that the reviewer highlights the mechanistic link between Rb, KDM5A and ER signalling as a key aspect of the study.

1) **There is certainly some novelty in demonstration of a role of Rb as a transcriptional activator, specifically through the inhibition of KDM5A. However the induction of CCND1 has previously been shown and this has not been referenced (eg DOI: 10.1158/1535-7163.MCT-17-0290). This has also been shown in ER negative cell lines and it is not clear why the authors have limited this paper to 2 ER positive cell lines only. It is an intriguing thought that ER could potentially be derepressed in ER negative cell lines. I would like to see these data to enhance the novelty of the paper.**

   As the reviewer notes, our primary analyses are performed in ER-positive models. In this context, we provide direct molecular evidence that hypo-phosphorylated Rb promotes activation of oestrogen-responsive genes, and that this effect is dependent on functional ER, as demonstrated by suppression of this transcriptional program with ER degraders.

   To address whether this mechanism extends to ER-negative settings, we analysed RNA-seq data from ER-negative breast cancer cell lines (MDA-MB-231 and MDA-MB-453) treated with CDK4/6 inhibitors. In these models, we do not observe induction of oestrogen response gene sets, nor do we detect upregulation of *ESR1* expression (Extended Data Fig. 5b and 5c in revised manuscript). These findings indicate that Rb-dependent activation of oestrogen-responsive transcription requires the presence of functional ER and is not a general consequence of CDK4/6 inhibition across breast cancer subtypes. We interpret this as a context-dependent mechanism in which Rb engages pre-existing ER transcriptional circuitry, rather than inducing ER signalling de novo.

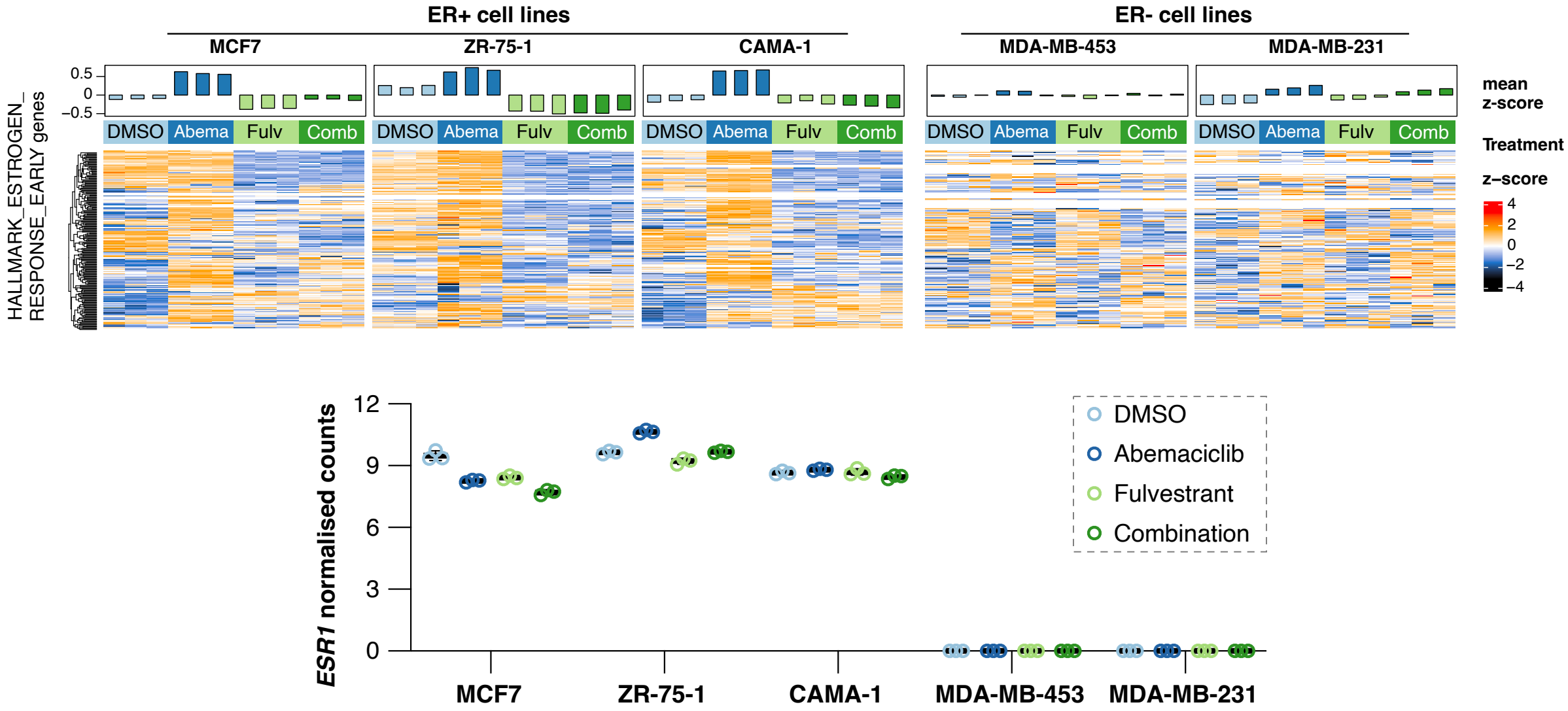

Top: ER+ and ER- breast cancer cell lines were treated with abemaciclib, fulvestrant, or the combination for 5 days and RNA-seq was performed. Heatmap demonstrates expression of genes from the *Hallmark Estrogen Response Early* gene set. Bar plots show mean z-score for all genes within this gene set across samples and condition (Extended Data Fig. 5c in revised manuscript).

Bottom: Expression of the *ESR1* locus in the same experiment (Extended Data Fig. 5b in revised manuscript).

Despite these observations, we agree with the reviewer that induction of *CCND1* following CDK4/6 inhibition has been reported previously, and we have incorporated the suggested reference into the revised manuscript (line 226). We also acknowledge that in ER-negative contexts, *CCND1* upregulation may be mediated by alternative transcriptional regulators. However, our data indicate that in ER-positive models, induction of *CCND1* occurs within a broader Rb-dependent, ER-driven transcriptional program. This is supported by the requirement for functional ER, the coordinated upregulation of multiple ER target genes, and the suppression of this program by ER degradation. We therefore interpret *CCND1* induction in this setting as part of an ER-dependent transcriptional response, rather than a general or isolated effect of CDK4/6 inhibition.

2) **The significance in clinical translation even in ER+ breast cancer, is less well evidenced and could be strengthened. In ER expressing breast cancers CDK4/6 inhibitors are almost always administered with an estrogen targeting approach, either aromatase inhibition or a SERM/SERD. The authors demonstrate the Rb induced activation of ER can be antagonised by ER targeting as expected. So how is this relevant to BC treatment? They hypothesise that the new found role of Rb "may even explain the capacity of CDK4/6 inhibition to restore endocrine sensitivity in resistant tumours." This should be investigated in this paper in my opinion. The in vivo and in vitro experiments in supplementary figure 4 could have been extended to look at resistance emergence with examination of the impact of Rb in this setting. This would significantly strengthen the paper and potentially provide extremely useful data for translational studies.**

We agree with the reviewer that CDK4/6 inhibitors are administered in combination with ER-directed therapy in clinical practice, and that the translational relevance of our findings should be clearly articulated. A key point of our study is that, despite the widespread use of this combination, the molecular basis for the strong synergy between CDK4/6 inhibition and endocrine therapy has remained incompletely defined. <u>Our data provide a mechanistic explanation for this interaction,</u> demonstrating that pharmacologic activation of Rb induces ER-dependent transcriptional programs that can be effectively suppressed by ER-targeted therapies. *In addition, these findings have*

fundamental significance, as they establish that Rb can directly promote gene expression, including pro-proliferative targets, thereby expanding the current understanding of Rb function beyond transcriptional repression.

Beyond this, our findings have relevance in the context of persistent or ligand-independent ER signalling. As shown in Extended Data Fig. 6, *ESR1*-Y537S cells exhibit sustained proliferation under combined CDK4/6 inhibition and oestrogen deprivation, whereas wild-type cells remain suppressed under the same conditions. In this setting, persistent ER activity is associated with continued expression of ER target genes, including *CCND1*, and a failure to achieve deep suppression of E2F target genes. This incomplete cell cycle repression is accompanied by progressive cell outgrowth over time, linking sustained ER signalling to functional escape from combination therapy.

We have now strengthened these observations with additional mechanistic data. Pharmacologic ER degradation using a SERD suppresses ER-driven transcription, restores repression of E2F target genes, and prevents cell outgrowth in *ESR1* mutant cells (Extended Data Fig. 6d and 6e). Similarly, targeted suppression of a key ER effector, *CCND1*, enhances E2F target gene suppression under CDK4/6 inhibition and oestrogen deprivation (Fig. 3f). Together, these findings support a model in which Rb-driven ER transcription, when not adequately suppressed, can maintain cell cycle gene expression and limit the depth and durability of response to combination therapy. We hope that this data collectively serves to emphasise the translational relevance of our study.

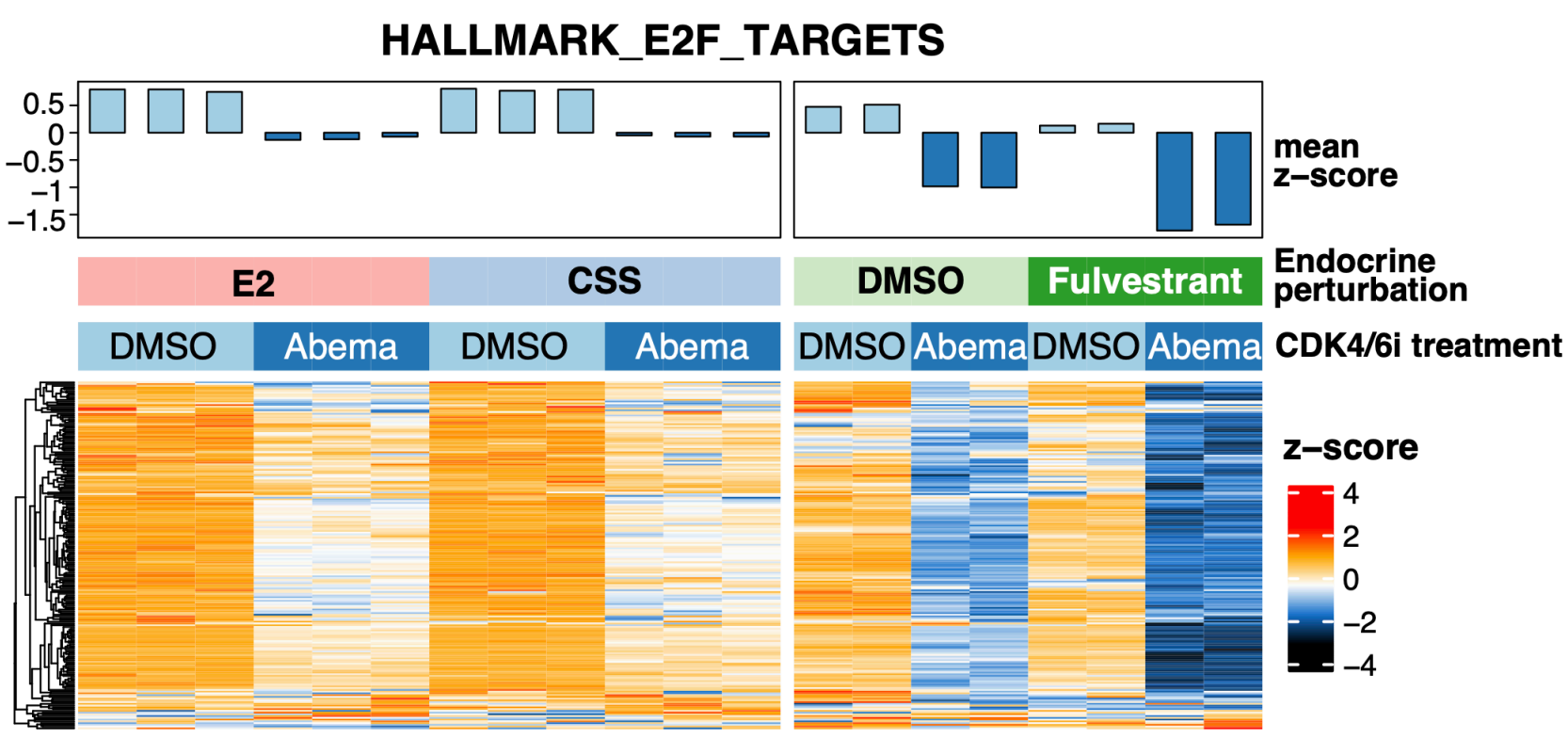

MCF7 *ESR1*-Y537 cells were treated with drugs indicated for 7 days and RNA-seq was performed. Heatmap show expression of E2F target genes. Note that estrogen deprivation (CSS) does not deepen suppression of E2F targets when added to abemaciclib, but fulvestrant (a SERD) does (Extended Data Fig. 6d in revised manuscript).

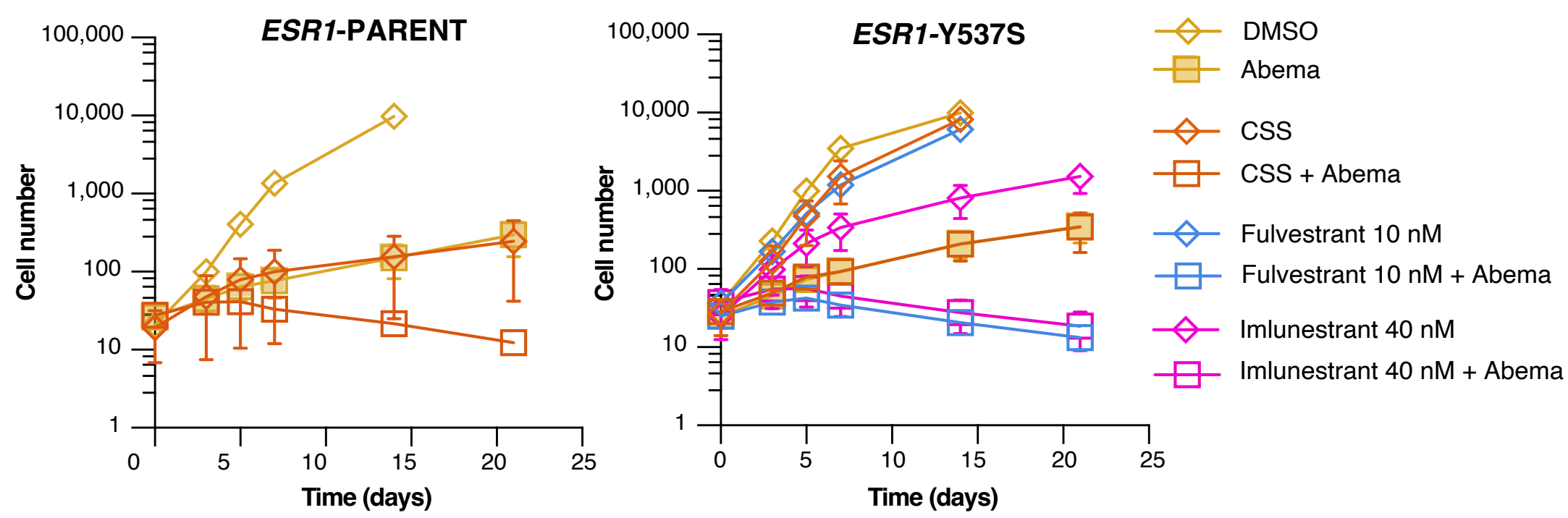

Cell number of MCF7 *ESR1*-Parent or *ESR1*-Y537S mutant cells expressing H2B-GFP grown in complete medium treated with DMSO, abemaciclib, fulvestrant, or imlunestrant, or in charcoal stripped serum (CSS) treated with DMSO or abemaciclib, tracked over 21 days (n = 4 separate wells per group; Extended Data Fig. 6e in revised manuscript).

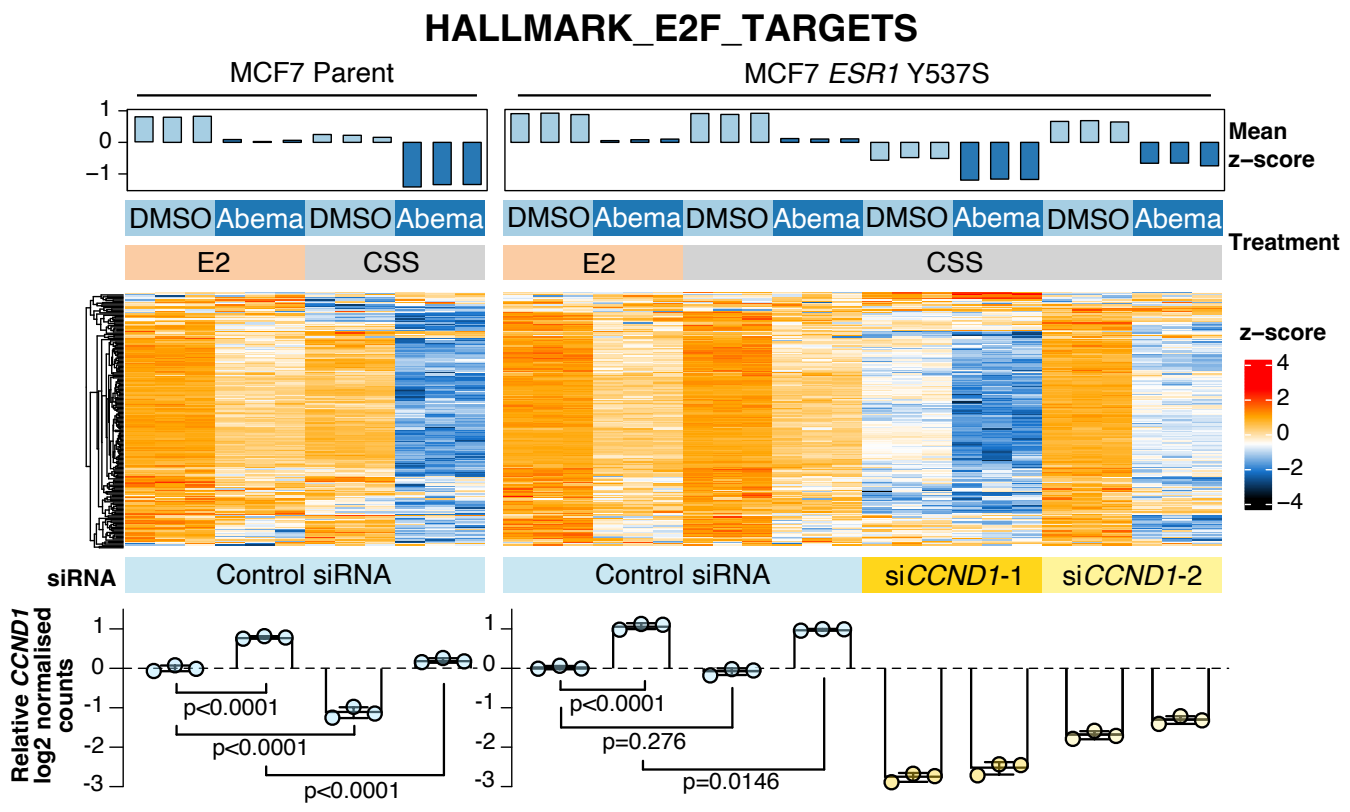

MCF7 cells (parent or *ESR1*-Y537S as indicated) were treated with drugs shown for 5 days, with or without concomitant knockdown of *CCND1,* and RNA-seq was performed. Heatmap shows expression of genes from the *Hallmark E2F Target* gene set. Bar plot below shows extent of *CCND1* knockdown with each siRNA used (Fig. 3f in revised manuscript).

Finally, we agree that modelling acquired endocrine-resistant tumours would further extend the translational impact of this work. However, such studies require long-term evolutionary systems that are beyond the scope of the current manuscript. We have therefore revised the text to more precisely define the translational implications of our findings and to avoid overinterpretation in the context of resistance (lines 241-254, 325-330).

3) **The concentrations of abemaciclib and palbociclib used in this manuscript are well above the published IC50s (eg DOI: 10.1158/1535-7163.MCT-17-0290) and at least twice the Cmax levels in human PK studies (therefore far above steady state levels). In vivo dosing is also high and this is evident from the growth curves presented in supplementary figure 4 b-g where synergy is less at the dose levels used in most of the studies and there is little additional benefit seen with combination vs single agent therapy. The translational relevance of the study would be significantly enhanced if drug concentrations were used that were achievable clinically. This is made all the more relevant with data demonstrating off target kinase inhibition, which differs between the CDK4/6 inhibitors, and may go some way to explain the differences in patterns of gene expression seen with abemaciclib and palbociclib in this study (eg figure 2a; reference that could be discussed doi. 10.1158?1541-7786.MCR-17-0468.). The off target effects (eg Wnt activation) may also impact on 'differentiation' (see below) and it would be useful to see the chromatin interaction hubs with palbociclib (figure 2c) and to see more discussion of this as a drawback.**

In vitro dosing used in this study

We agree with the reviewer that drug concentration is an important consideration, particularly when interpreting translational relevance. Our primary aim in these experiments was to achieve robust and uniform Rb hypo-phosphorylation in order to enable mechanistic interrogation of Rb-dependent chromatin and transcriptional effects. We acknowledge that some of the concentrations used in the manuscript exceed clinically reported plasma levels. However, direct comparison between *in vitro* concentrations and clinical exposures is not straightforward, as these agents are highly protein bound and free drug levels differ substantially between cell culture conditions and human plasma. We therefore focused on achieving a consistent biological effect – namely Rb activation – as a benchmark across experiments.

Importantly, the key transcriptional and chromatin features described in this study are preserved at substantially lower concentrations. RNA-seq performed at 100nM abemaciclib demonstrates induction of oestrogen response gene sets, and CUT&RUN experiments at this dose show similar

redistribution of Rb to regulatory regions associated with ER target genes, albeit with reduced magnitude. We include this data here for the reviewer's consideration.

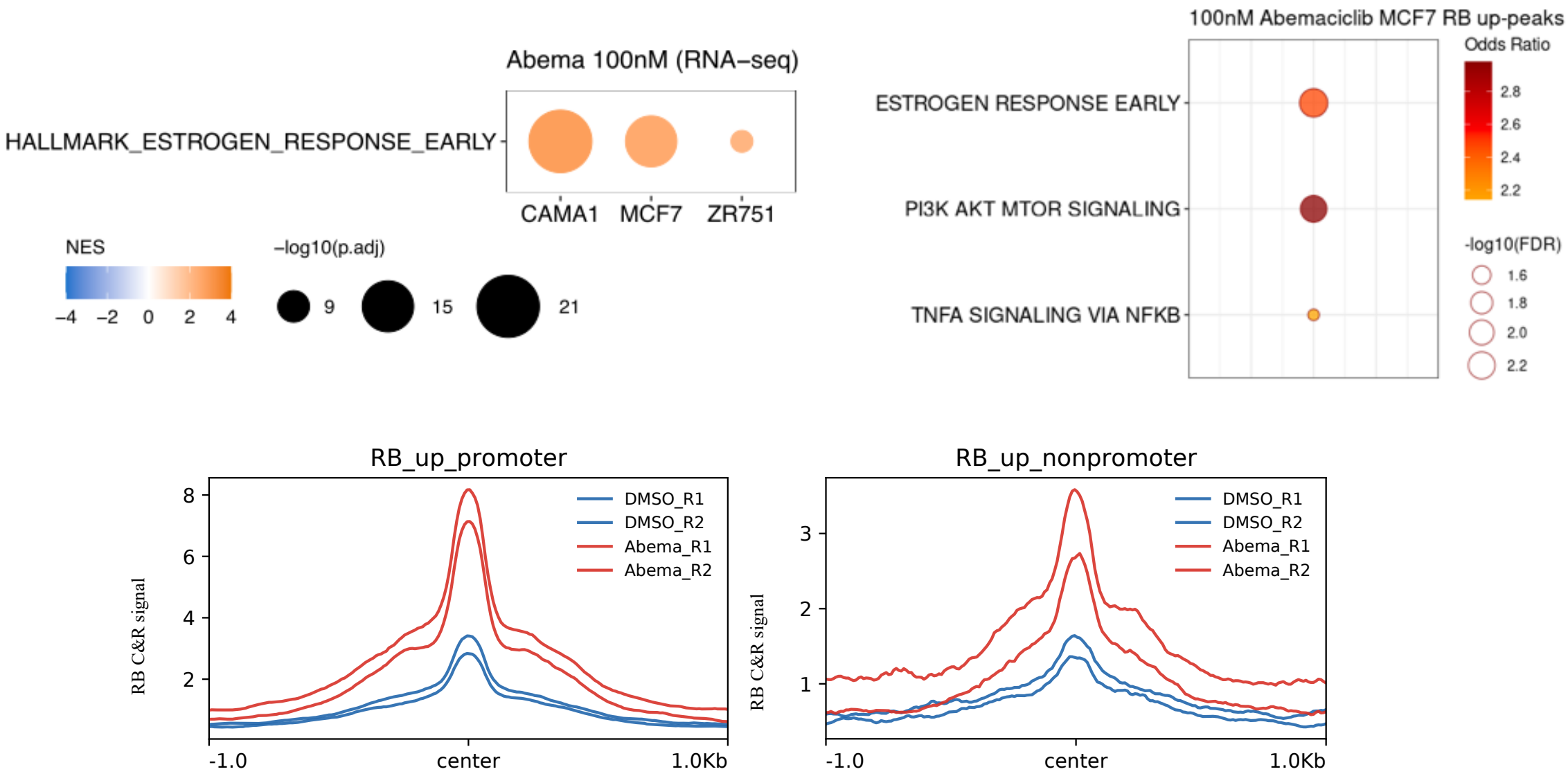

Top left: GSEA of RNA-seq data showing enrichment of the *Hallmark Estrogen Response Early* gene set in CAMA1, MCF7 and ZR751 cells treated with 100 nM abemaciclib for 5 days. CAMA1 (NES = 2.81, adjusted $P = 4.62 \times 10^{-22}$), MCF7 (NES = 2.49, adjusted $P = 1.07 \times 10^{-15}$) and ZR751 (NES = 2.14, adjusted $P = 5.53 \times 10^{-9}$).

Top right: ChIP-Enrich analysis of genes whose transcription start sites are closest to RB non-promoter up-peaks in MCF7 cells treated with 100 nM abemaciclib for 5 days (Odds Ratio = 2.47, adjusted P = 0.005 for the estrogen early geneset).

Bottom: Composite plot showing RB signal intensity at promoter and non-promoter up-peaks in MCF7 cells treated with 100nM abemaciclib (genomic regions identified in MCF7 cells treated with 500 nM abemaciclib).

These data are included for reviewer's reference.

In addition, dose-response analyses of E2F target genes across 100, 500, and 1000 nM abemaciclib show that CDK4/6 inhibition alone does not fully suppress these genes even at higher concentrations, whereas the addition of fulvestrant is required for complete repression. Together, these data indicate that the core biological effect - Rb-dependent activation of ER signalling and its impact on cell cycle control - is not restricted to higher drug concentrations.

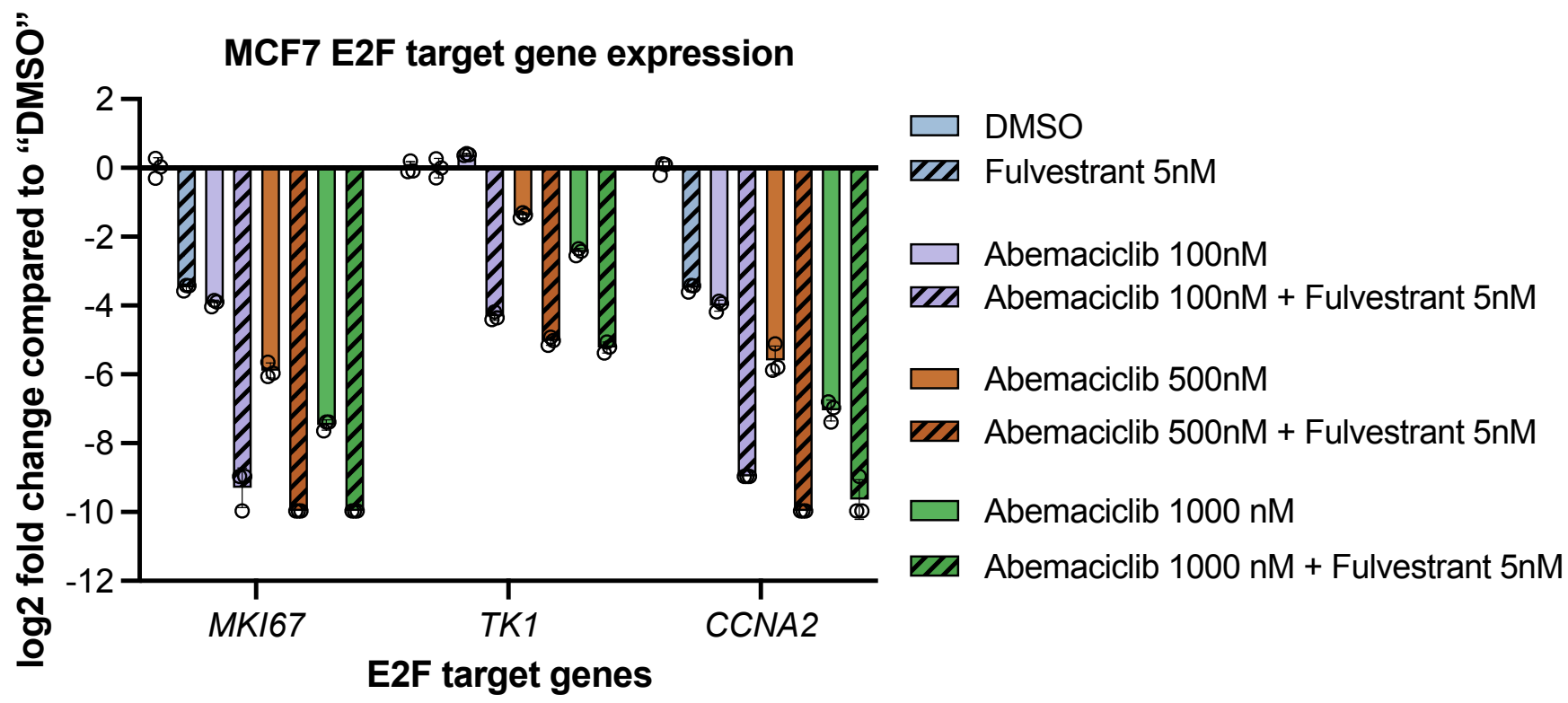

Expression of E2F target genes in MCF7 cells treated with abemaciclib and fulvestrant for 5 days, measure by RT-qPCR. Three replicates per treatment group.

These data are included for reviewer's reference.

<u>Combination effects and *in vivo* dosing</u>

With respect to the reviewer's concern regarding combination benefit, we note that the *in vitro* assays (Extended Data Fig. 4b-f in original draft; Extended Data Fig. 5d-h in revised manuscript)

and the *in vivo* experiment (Extended Data Fig. 4g in original draft; Extended Data Fig. 6a in revised manuscript) represent distinct systems. In the PDX model, the combination does provide additional benefit over single-agent treatment, consistent with the mechanistic interaction described in the manuscript. We agree, however, that dose selection can influence the apparent magnitude of synergy, and hope the data provided here provide some confidence in the relevance of our findings at lower doses.

Differences between abemaciclib and palbociclib
With respect to differences between abemaciclib and palbociclib, we agree that off-target kinase inhibition may contribute to differences in gene expression profiles. However, the key observations in this study, including Rb redistribution and activation of ER-responsive transcription, are observed with both CDK4/6 inhibitors, supporting a class effect linked to Rb activation (Fig. 1a-e, Extended Data Fig. 1e-f, Fig. 2a and 2h). Importantly, genetic ablation of *RB1* prevents induction of ER target genes following CDK4/6 inhibition (Fig. 2e and 2i, and Extended Data Fig. 4h). In addition, CRISPR-mediated disruption of Rb binding sites at regulatory regions of specific ER target genes similarly attenuates their induction (Fig. 2f and 2g, and Extended Data Fig. 4c-e). Together, these findings demonstrate that this transcriptional response is dependent on Rb and its chromatin engagement, rather than a consequence of off-target drug activity.

Finally, we agree that additional analysis of chromatin interaction hubs following palbociclib treatment would be informative. While matched HiChIP data are not currently available for palbociclib-treated cells, we have performed H3K27ac ChIP-seq under these conditions and observe similar patterns of chromatin activation at ER-associated regulatory regions at non-promoter Rb up-peak regions, at both the ChIP-seq ORA and HiChIP ORA levels, as seen with abemaciclib. These findings support the conclusion that the chromatin and transcriptional effects described are not specific to a single CDK4/6 inhibitor.

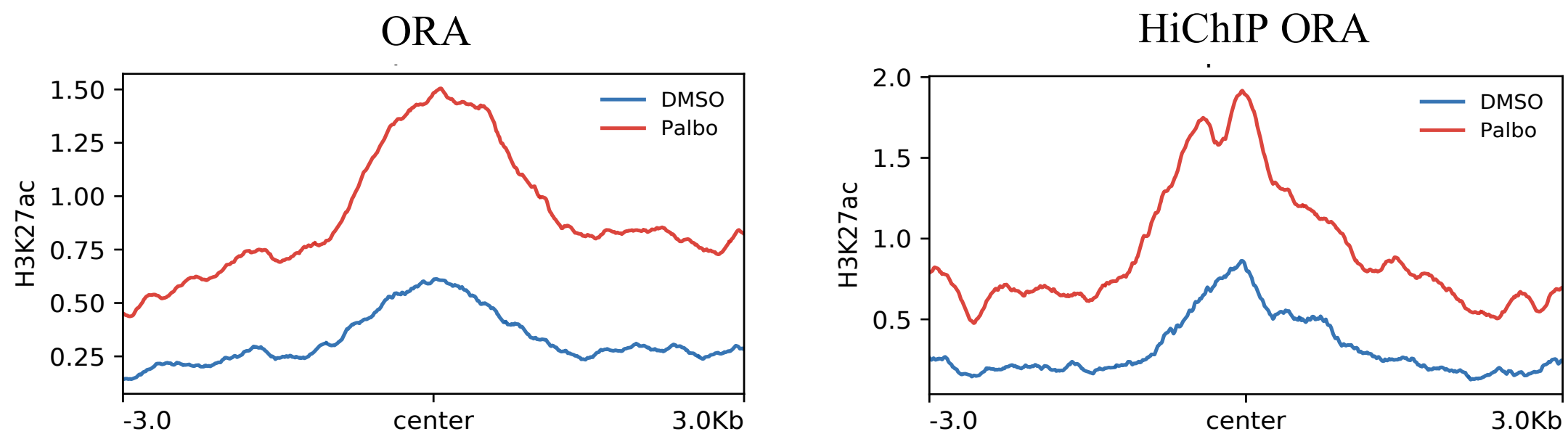

Composite plot showing H3K27ac signal intensity at Rb non-promoter up-peaks associated with *Hallmark Estrogen Response Early* genes from the ORA (Fig. 2a) and HiChIP ORA (Extended Data Fig. 3i) in MCF7 cells treated with 500 nM abemaciclib for two days. The plots above are included here for the reviewer's reference.

4) **The interpretation that their findings in this paper "..the pharmacological activation of Rb.... reveals its role in enforcing lineage identity in tumour cells through enhancer-mediated gene activation" appears to draw more on their previous work. In this paper the term 'lineage' comes in the abstract and discussion only and one reference on B cells. There is no description of what is meant by this and no justification for the use of the term. Induction of ER in ER positive breast cancer cell lines can hardly be said to alter the lineage of those cell lines and the theory seems mainly to be a hangover from their 2021 paper that included both ER positive and negative cell lines (although did not look at ER transcription).**

We agree with the reviewer that the term "lineage identity" was not clearly defined in the context of this study and may have overstated our conclusions. Our intention was not to suggest that

CDK4/6 inhibition induces a change in cellular lineage, but rather that activation of Rb reinforces pre-existing, lineage-associated transcriptional programs.

In ER-positive breast cancer models, oestrogen receptor target genes represent a core component of luminal lineage transcriptional output. Our data show that CDK4/6 inhibition enhances expression of these ER-regulated genes in an Rb-dependent manner, indicating that Rb can strengthen this existing transcriptional program. We have therefore revised the manuscript to replace the term "lineage identity" with more precise language describing reinforcement of ER-dependent transcriptional programs (e.g. lines 54, 313-314, 342, and 345).

5) **As above, why not determine the impact on ER signalling in ER negative cells as previous studies have demonstrated increased CCND1 expression in multiple TNBC cell lines and xenografts (DOI: 10.1158/1535-7163.MCT-17-0290).**

   Please see our response to comment (1) above.

<u>Response to Referee #3</u>

Reviewer Summary: This manuscript reports a novel function of the retinoblastoma (Rb) protein that activates a pro-proliferative gene program following treatment of breast cancer models and patients with CDK4/6 inhibitors. Rb is well known as a tumor suppressor that represses expression of cell cycle genes and is inactivated by CDK4/6 phosphorylation in breast and other cancers. While CDK4/6 inhibitors such as abemaciclib are used in the clinic, their efficacy is narrowly limited to certain cancer types, and they are used in combination therapy that also downregulates estrogen signaling in HR-positive breast cancer for reasons that are not completely understood. Here, using the Cut&Run approach for unbiased genomic screening, the authors identified a set of previously uncharacterized sites that are bound by Rb in response to abemaciclib treatment. These sites are outside of typical cell-cycle gene promoters and are correlated with signatures of gene activation. Remarkably, the authors link the presence of Rb at such gene enhancer regions to activation of several estrogen-responsive genes, including the gene encoding CycD1, which may explain why CDK4/6 inhibitors and anti-estrogen receptor (ER) therapies cooperate in certain breast cancer treatments. Finally, a mechanism for gene activation is supported in which Rb binds and inhibits the histone demethylase KDM5A.

Considering the great potential of using cell-cycle inhibitors as cancer therapies and the need to understand the response to CDK inhibitors in the tumor, the significance of this research is high. Several of the specific findings here are not novel, which may limit the appeal of the report to those interested in cancer biology and therapies. For example, that RB binds the genome at sites outside of cell-cycle genes, that RB antagonizes KDM5A to activate transcription, and that expression of CycD1 and other ER genes underlies the need for dual ER and CDK4/6 inhibition in therapy have been studied. On the other hand, the linking of these ideas in a mechanism that follows CDK4/6 inhibitor treatment will be an advance in our understanding of how RB pathway biology and these inhibitors impact each other. The genomic profiling experiments here are thorough, and there are several key experiments, including the RB and RB binding-site KO experiments, that provide strong support for the model. The overall experimental approach, which includes multiple model cell lines and patient samples, is for the most part robust.

We thank the reviewer for this thoughtful and balanced assessment of our work. We appreciate their recognition of the conceptual advance in linking Rb chromatin binding, KDM5A antagonism, and ER-dependent transcription in the context of CDK4/6 inhibition. We are also encouraged that the reviewer highlights the strength of the genomic profiling and the genetic perturbation experiments in supporting our model.

1) **Tables of genes for Rb-up peaks at promoters and at nearby sites should be included. For example, lists of genes used for analysis in Fig. 1e and especially Fig. 2a should be provided.**

   We thank the reviewer for this suggestion. We have provided tables that clearly identify the genes that were used in the analyses for Fig. 1e and 2a and included these as Supplementary Tables 1,2, and 4. Specifically, the tables added are as follows:

	**Table Title**
**Supplementary Table 1**	Genes with RB promoter peaks gained with abemaciclib treatment, used for ChIPEnrich analysis (Fig. 1e).
**Supplementary Table 2**	Genes with RB nonpromoter peaks gained with abemaciclib (LY) or Palbociclib (PB) treatment, used for ChIPEnrich analysis (Fig. 2a).
**Supplementary Table 4**	Genes with RB nonpromoter peaks gained with abemaciclib (LY) or Palbociclib (PB) treatment, used for ORA analysis (Fig. 2a).

2) **Lines 129-136: The inclusion of the HiChIP data here adds little support to the overall model, and the conclusion is potentially misleading. The increased number of H3K27ac-marked noted and interconnections at hubs with nonpromoter Rb peaks reflects the chromatin structure at these locations but may be unrelated to the presence of Rb. Moreover, while H3K27ac may be correlated with transcriptional activity, there is lack of data here for transcriptional activity at those sites. Therefore, the conclusion that there is an "association between Rb's integration into regulatory networks and the transcriptional activity of those networks" is overstated from this analysis.**

We agree with the reviewer that the original wording overstated the conclusions that could be drawn from the HiChIP analysis. To further address this point, we have added a new analysis (Extended Data Fig. 3d) examining transcriptional output at genes associated with these hubs. Specifically, we show that genes residing in hubs containing a non-promoter Rb binding site exhibit greater transcriptional activation in response to abemaciclib treatment compared to genes in hubs lacking such sites, and that this difference is abolished in *RB1* knockout cells – supporting an Rb-dependent contribution to this effect. In line with these additional data, we have revised the original text (lines 141-142).

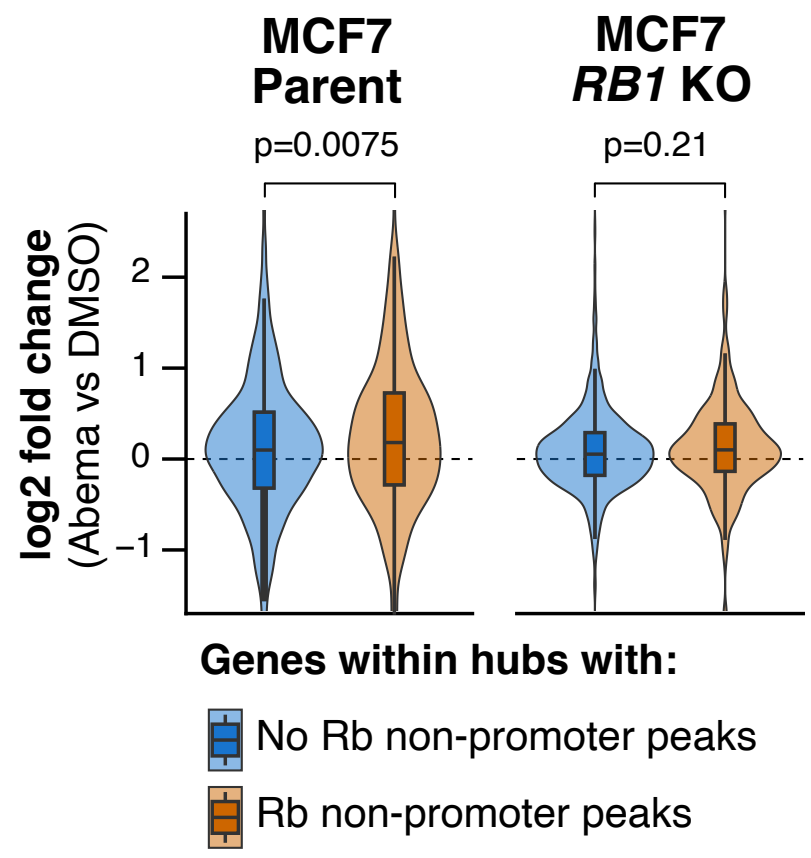

Log$_2$ fold change (DESeq2) in expression of genes residing within hubs containing non-promoter Rb up-peaks (orange; 464 genes), or hubs lacking such sites (blue; 1552 genes), in parental and isogenic *RB1* knockout MCF7 cells following five days of abemaciclib treatment compared to DMSO. Abemaciclib versus DMSO, 5 days of treatment. Statistical significance was assessed using a two-sample t-test. (Extended Data Fig. 3d).

3) **Line 150: How many out of the 3878 / 2240 RB non-promoter peaks are actually connected to genes upregulated by abemaciclib, i.e. how many genes are included in the analysis in Fig. 2a? What is the overlap in these genes between MCF-7 and ZR-75-1 cells?**

We thank the reviewer for raising this point. The question relates to the over-representation analysis (ORA) shown in Fig. 2a, where we sought to identify the genes that are located near Rb non-promoter up peaks and also show increased expression by RNA-seq. For MCF-7 cells, the ORA results identified 1,596 Rb non-promoter peaks associated with 1,185 distinct upregulated genes. In ZR-75-1 cells, the ORA results identified 591 peaks associated with 424 distinct upregulated genes.

The overlap between the two cell lines comprises 158 genes that are significantly upregulated in both models (Venn diagram provided for reference). While this number is modest, two observations are noteworthy. First, when considering all genes included across both cell lines, the majority show concordant upregulation across models, although statistical significance in both datasets is restricted to the shared subset. Second, **over-representation analysis of the 158 shared genes**

identifies a single significantly enriched gene set, *Estrogen Response Early* (adjusted *P* = 5.31 × 10^{-5}).

Together, these data indicate that genes proximal to non-promoter RB up-peaks show consistent transcriptional activation across cell lines, and that the shared, most robustly regulated subset is strongly enriched for estrogen response genes. All genes included in the ORA in Fig. 2a are provided in Supplementary Table 4.

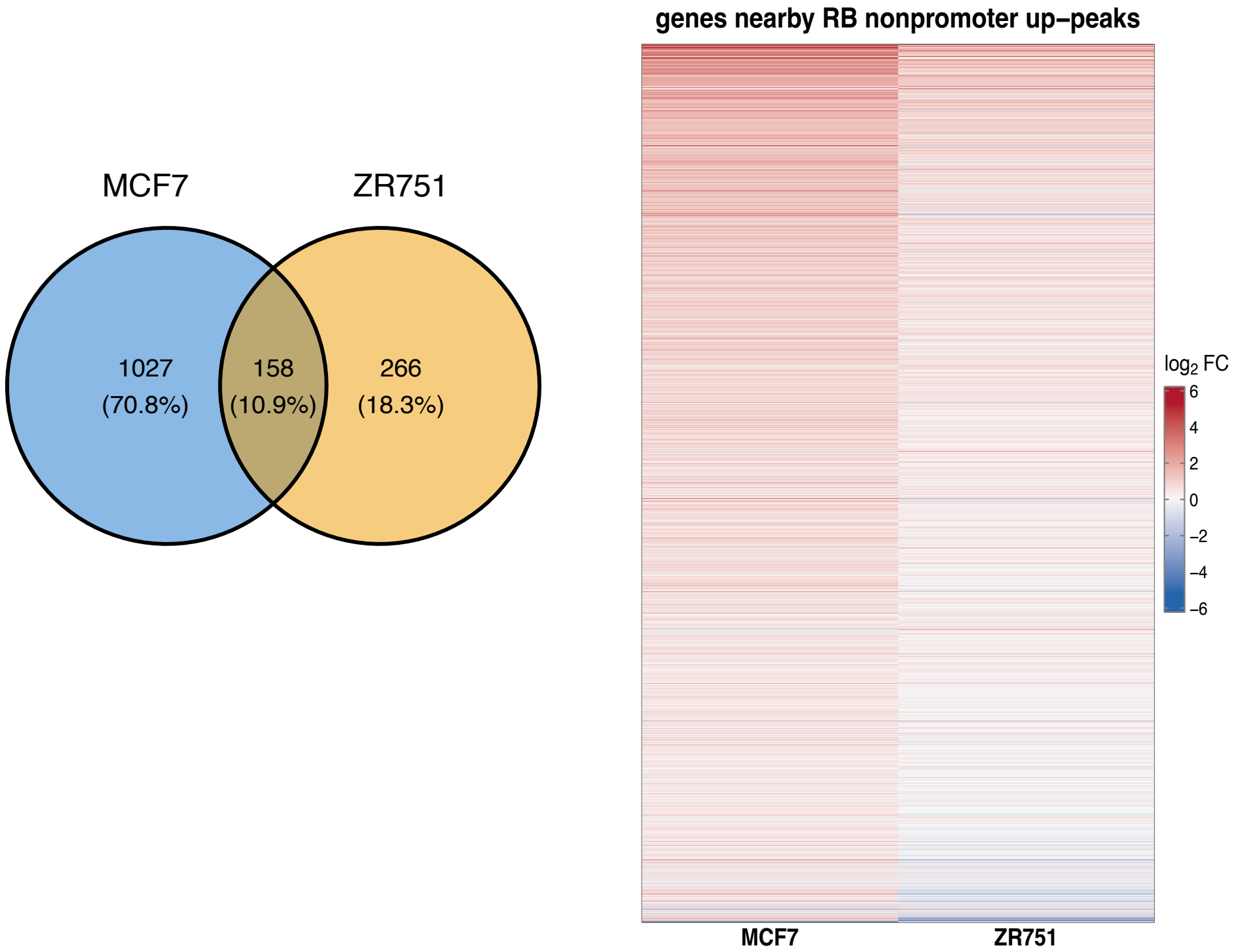

Left: Venn diagram showing the overlap of significantly upregulated genes associated with Rb non-promoter up-peaks in MCF7 and ZR-75-1 cells (genes used in the ORA analysis in Fig. 2a).

Right: Heatmap showing log_2 fold change of all genes associated with Rb non-promoter up-peaks in MCF7 and ZR-75-1 cells.

Data included here for reviewer's reference.

4) Lines 139-142: How many genes with non-promoter Rb-up peaks induced by abemaciclib are estrogen response genes? Provide a table with gene names.

Lines 139–142 of the original manuscript describe the ChIP-Enrich analysis of Rb non-promoter up-peaks in abemaciclib-treated cells. In response to the reviewer's request, we have now included a supplementary table (Supplementary Table 3) listing the estrogen response genes identified in this analysis, defined as genes from the *Hallmark Estrogen Response Early* gene set associated with Rb non-promoter up-peaks. For clarity, we also summarise these genes below. Genes shared between both cell lines (*n* = 31) are indicated in red.

Sample	Treatment	N Genes	FDR	Gene Symbols (HALLMARK_ESTROGEN_RESPONSE_EARLY)
**MCF7**	Abema	73	5.89e-08	FAM102A, NCOR2, CCND1, CBFA2T3, HES1, IGF1R, RARA, STC2, SLC37A1, MLPH, AMFR, ELF3, FKBP5, MYBL1, SULT2B1, TFAP2C, ABHD2, TBC1D30, TSKU, TMEM164, NAV2, ABAT, ADCY1, BAG1, BCL2, CA12, CALB2, CELSR2, FLNB, GJA1, SFN, ITPK1, LAD1, MICB, MSMB, PGR, RET, SLC2A1, SOX3, SVIL, TFF1, KLF10, WFS1, SEMA3B, SLC7A5, NRIP1, ANXA9, BHLHE40, SYNGR1, FCMR, DHRS3, SLC9A3R1, PTGES, CELSR1, GREB1, TOB1, SLC27A2, PRSS23, RHOBTB3, KDM4B, KAZN, WWC1, MED13L, SLC39A6, LRIG1, TJP3, NADSYN1, MREG, HR, CYP26B1, ELOVL5, DEPTOR, BCL11B
**ZR751**	Abema	46	4.64e-05	NCOR2, CCND1, CBFA2T3, HES1, BHLHE40, EEIG1, ALDH3B1, BLVRB, MPPED2, CLDN7, ELF3, FKBP5, SFN, IGF1R, KRT8, MSMB, MYBL1, RET, SLC1A1, SLC2A1, SOX3, TFAP2C, WFS1, SEMA3B, STC2, FCMR, DHRS3, KLF4, NHERF1, PTGES, TOB1, SLC27A2, TTC39A, KDM4B, RRP12, MED13L, LRIG1, TJP3, NXT1, HR, ELOVL5, BCL11B, MLPH, ESRP2, ISG20L2, CANT1

5) Lines 158-163: For the set of 405 non-promoter Rb-bound elements that looped to gene promoters (considered in Extended data Fig. 3F), it is not clear to what extent these genes are upregulated following abemaciclib treatment. This is only described for the 17 genes from the Hallmark Estrogen Response Early set. Also, how many of these 405 RB-bound elements identified in MCF7 cells are also bound by RB in ZR-75-1 cells and the xenograft model, and how many of these genes are upregulated in ZR-75-1 cells after CDK4/6 inhibition? The authors should provide tables naming the genes in the particular groups, and venn diagrams showing the overlap between the cellular models. Furthermore, the authors should clearly show whether the subgroup that contains the 17 estrogen-responsive genes that have enhancer-bound RB-looping looks comparable in the three cellular models.

We thank the reviewer for these questions and agree that additional clarification strengthens the evidence supporting a direct role for Rb at enhancer elements linked to estrogen-responsive genes.

First, with respect to overlap between cell lines, of the 405 non-promoter Rb up-peaks looping to gene promoters in MCF-7 cells, 161 are also called as Rb up-peaks in ZR-75-1 cells (based on statistical thresholds). Importantly, when these 405 loci are examined as a group, we observe a consistent increase in Rb occupancy across these regions in ZR-75-1 cells as well as in the PDX, indicating that this pattern of Rb binding is not unique to MCF-7 cells but is shared across models.

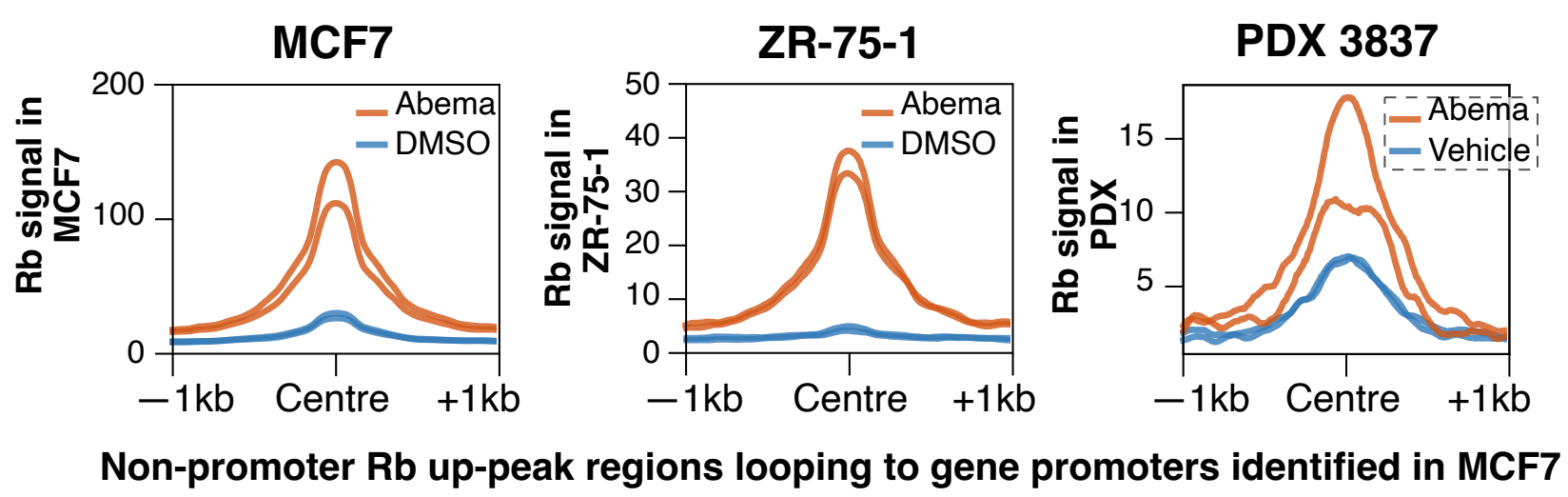

Composite profiles of Rb signal from MCF7, ZR-75-1, and PDX 3837 at the 405 non-promoter Rb up-peak regions identified in MCF7 using H3K27ac HiChIP that loop to gene promoters (Extended Data Fig. 3h).

Second, these 405 non-promoter Rb up-peaks in MCF-7 cells are associated with 344 genes (reflecting instances where multiple peaks loop to the same promoter). Of these genes, 31% (126/344) are significantly upregulated in expression following abemaciclib treatment in MCF-7 cells, 12% (41/344) in ZR-75-1 cells, and 28% (98/344) in CAMA-1 cells. Lists of all upregulated genes in each model are now provided in Supplementary Table 5, as requested by the reviewer.

To address overlap across models, we have included a Venn diagram showing the intersection of significantly upregulated genes among MCF-7, ZR-75-1, and CAMA-1 cells within this gene set. While the overlap of statistically significant genes (FDR < 0.05) is limited, the direction of expression change is broadly concordant across all three cell lines. **Notably, the subset of 17 estrogen response genes highlighted in the manuscript - defined as ER target genes with Rb non-promoter peaks looping to their promoters in MCF-7 cells - shows consistent upregulation across all three models.** This supports the conclusion that Rb-associated enhancer activity at ER target genes is a conserved feature across ER-positive breast cancer cell lines, rather than being specific to a single model.

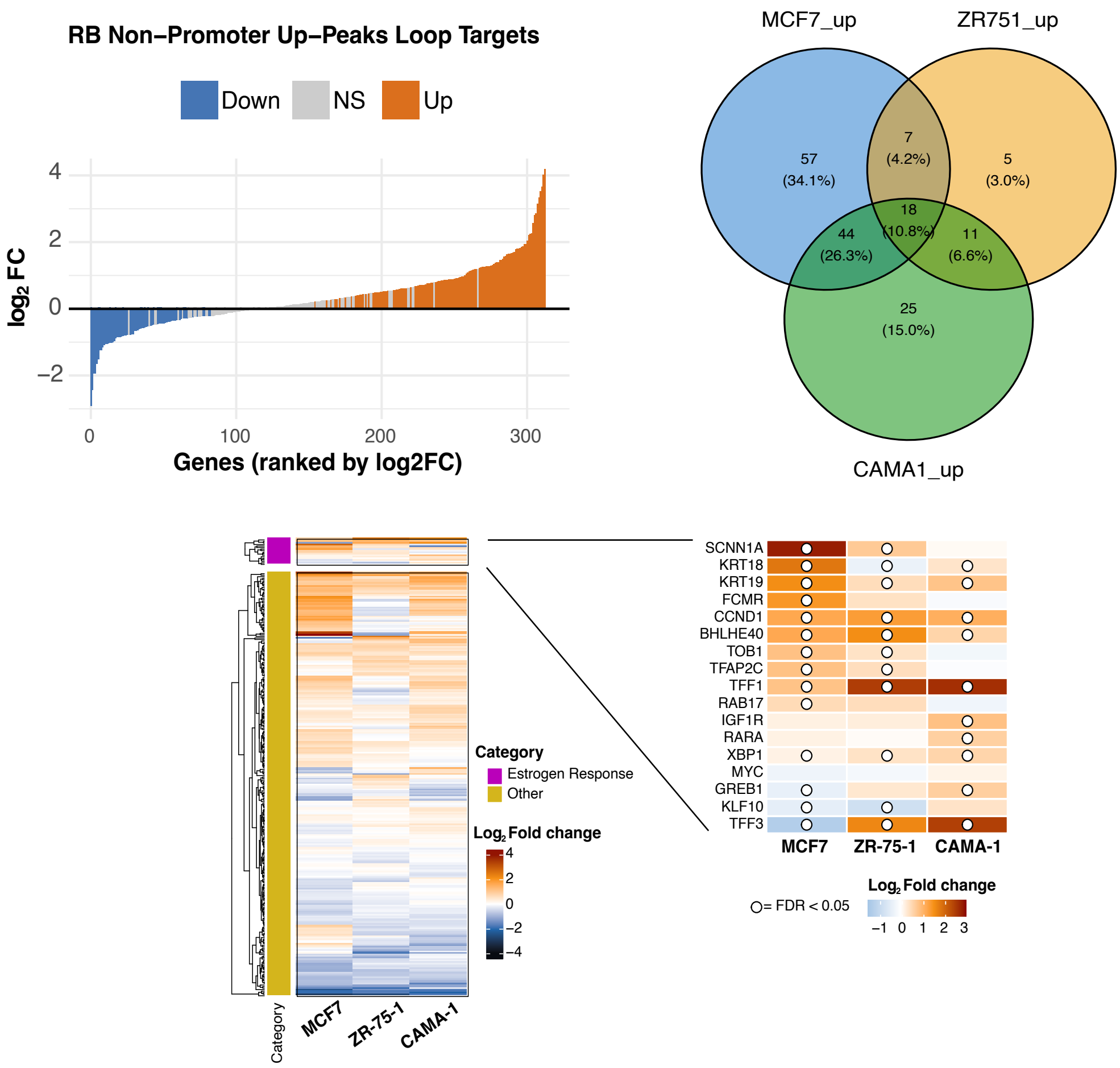

Top left: Waterfall plot showing expression fold change of genes that form loops with Rb non-promoter up-peaks in MCF7. Genes with significant changes in expression (adjusted $P < 0.05$) are shown in red for increased expression and blue for decreased expression, while non-significant genes are shown in grey. (Data included here for reviewer's reference.)

Top right: Venn diagram showing the overlap of significantly upregulated genes in MCF7, ZR-75-1, and CAMA-1 among genes that form loops with Rb non-promoter up-peaks in MCF7. (Data included here for reviewer's reference.)

Bottom: Heatmap showing $\log_2$ fold change of all genes that form loops with Rb non-promoter up-peaks in MCF7, across all three cell lines. The right panel shows an enlarged view restricted to estrogen response early genes. White circles denote statistical significance (adjusted $P < 0.05$). (Extended Data Fig. 4b).

Finally, we have substantially strengthened the *in vivo* component of the study through the generation of an entirely new dataset. In our original submission, we presented Rb CUT&RUN data from a PDX model and showed that CDK4/6 inhibitor treatment increases Rb occupancy at E2F target gene promoters (as expected). However, we were not able to confidently identify non-promoter peaks for technical reasons.

Since then, we have optimised the modified CUT&Tag protocol, DynaTag (Hunold, Pizzolato et al. 2025), to examine Rb chromatin occupancy in tissue. Compared to CUT&RUN, the DynaTag approach provides substantially improved signal-to-noise and peak detection in tissue, enabling confident identification of Rb binding events that were not resolvable in our original analysis.

We have now applied DynaTag to profile Rb in the estrogen-driven breast cancer PDX KCC-P-3837 model and include the data generated in our revised paper. As reported in our original draft, the PDX used (PDX-3837) shows upregulation of oestrogen response genes following CDK4/6 inhibitor treatment (Fig. 3b), consistent with our cell line models and samples from the POP clinical trial. Rb chromatin occupancy in the PDX recapitulates our findings from cell line models: (i) we observe a marked global increase in Rb chromatin occupancy following CDK4/6 inhibition, with the majority of Rb up-peaks located at promoters and enriched for E2F and cell cycle genes (Fig. 1e-h); (ii) importantly, we are also now able to confidently resolve non-promoter Rb up-peaks in tumour tissue. These non-promoter peaks show strong concordance with those identified in cell lines, including direct overlap at enhancers adjacent to key ER target genes such as *CCND1* (Extended Data Fig. 3f). Consistent with our *in vitro* findings, ChIP-Enrich analysis of these non-promoter Rb peaks identified the Hallmark *Estrogen Response Early* gene set as the top enriched pathway (Fig. 2a).

Taken together, this new *in vivo* dataset provides orthogonal validation of both the canonical and non-canonical functions of Rb, demonstrating that the redistribution of Rb to non-promoter regulatory elements linked to estrogen-responsive genes is not a cell line-specific phenomenon but is observed in tumour tissue. More broadly, to our knowledge, this represents the first genome-wide profiling of Rb chromatin occupancy in any tissue context, and substantially strengthens the physiological and clinical relevance of our findings.

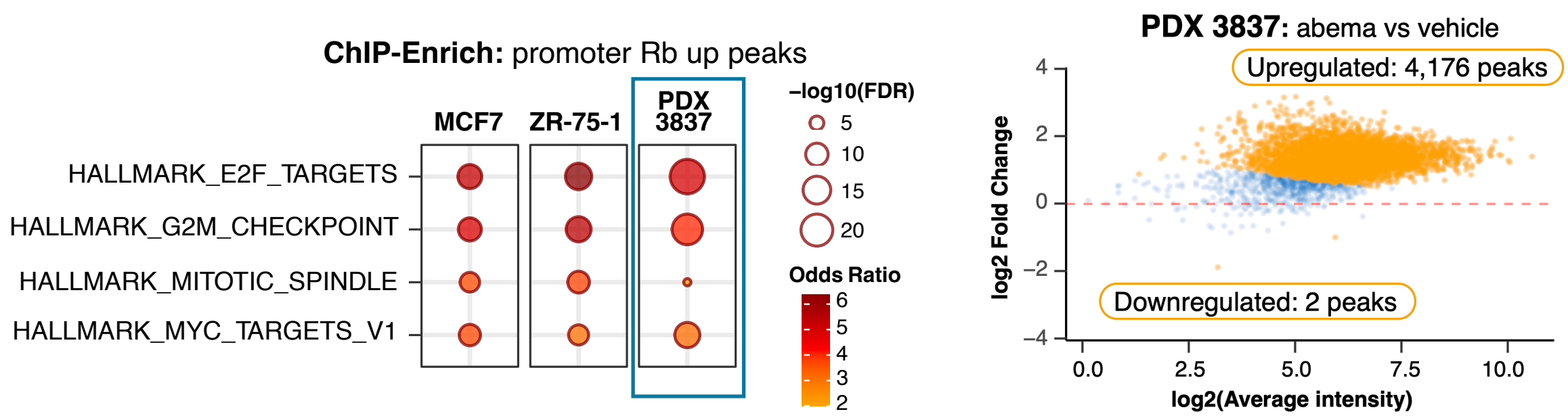

Left: ChIP-Enrich analysis of Rb promoter up peaks in MCF7 and ZR-75-1 cells treated with abemaciclib compared to DMSO (defined in Fig. 1**e**) and in a breast cancer patient-derived xenograft model (PDX KCC-P-3837) treated with abemaciclib compared to vehicle ($\log_2$ fold change > 0; adjusted $P < 0.2$). Showing the top three gene sets based on lowest adjusted P values from any of the cell lines or PDX; only data points of gene sets with adjusted $P < 0.05$ are shown. (Fig. 1e in revised manuscript).

Right: MA plot representations of differential analysis of Rb chromatin binding detected by DynaTag in PDX KCC-P-3837 treated for 4 days with abemaciclib compared to vehicle control. Each dot represents a genomic region of 400 bp width: orange indicates differential Rb signal with adjusted $P < 0.2$, and blue indicates adjusted $P \geq 0.2$ (Fig. 1f in revised manuscript).

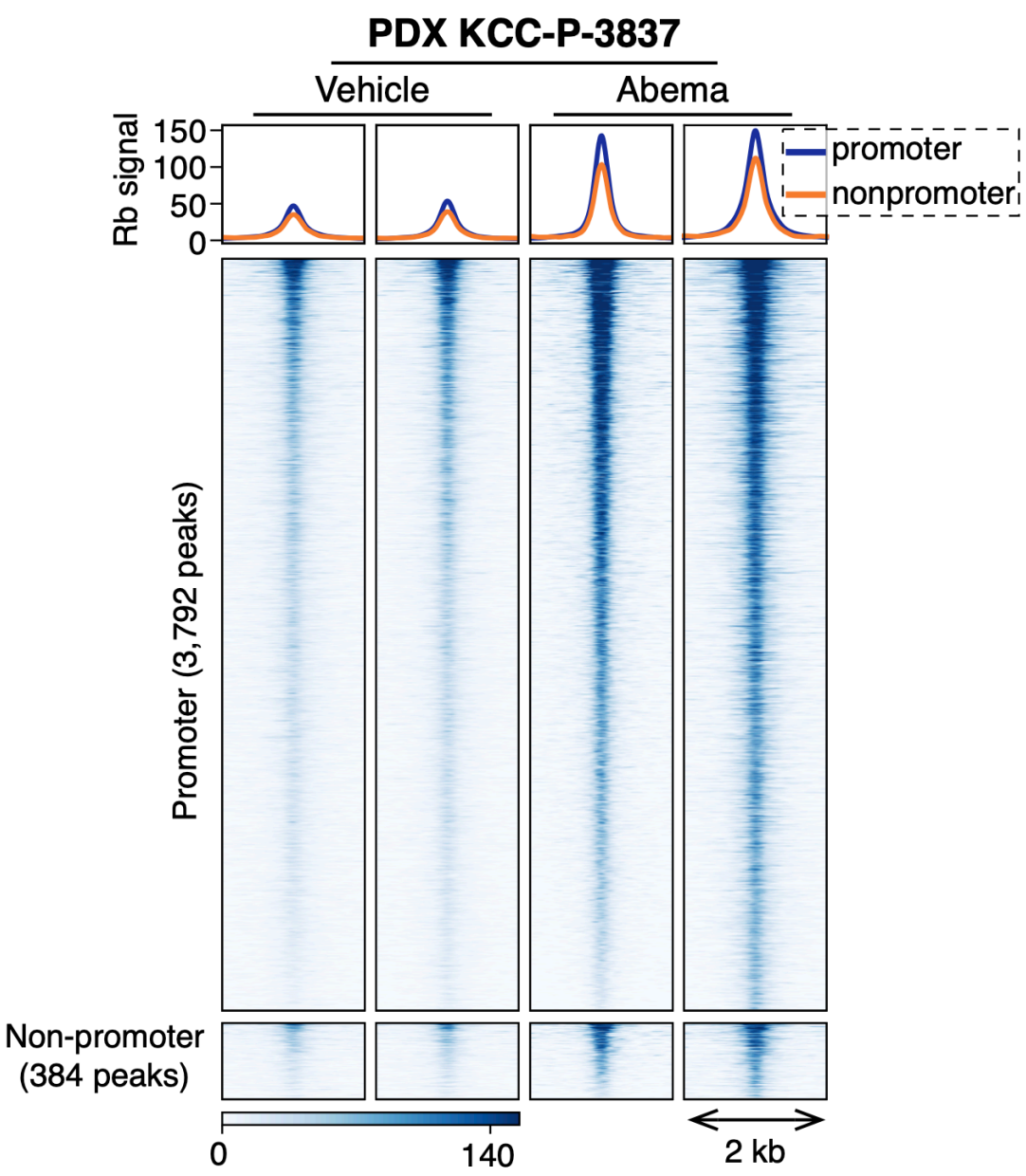

Composite profiles (top) and heatmaps (bottom) of Rb DynaTag signal at Rb up peaks in PDX-3837 tumours (Fig. 1h in revised manuscript).

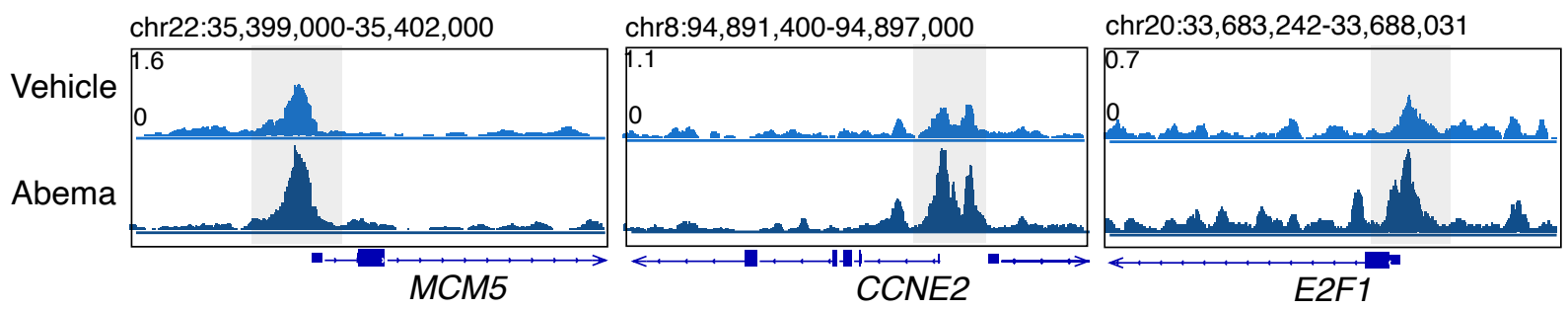

Representative PDX KCC-P-3837 Rb signal tracks at E2F target gene promoter regions. Grey highlights indicate Rb up peaks at gene promoters. (Extended Data Fig. 2f in revised manuscript).

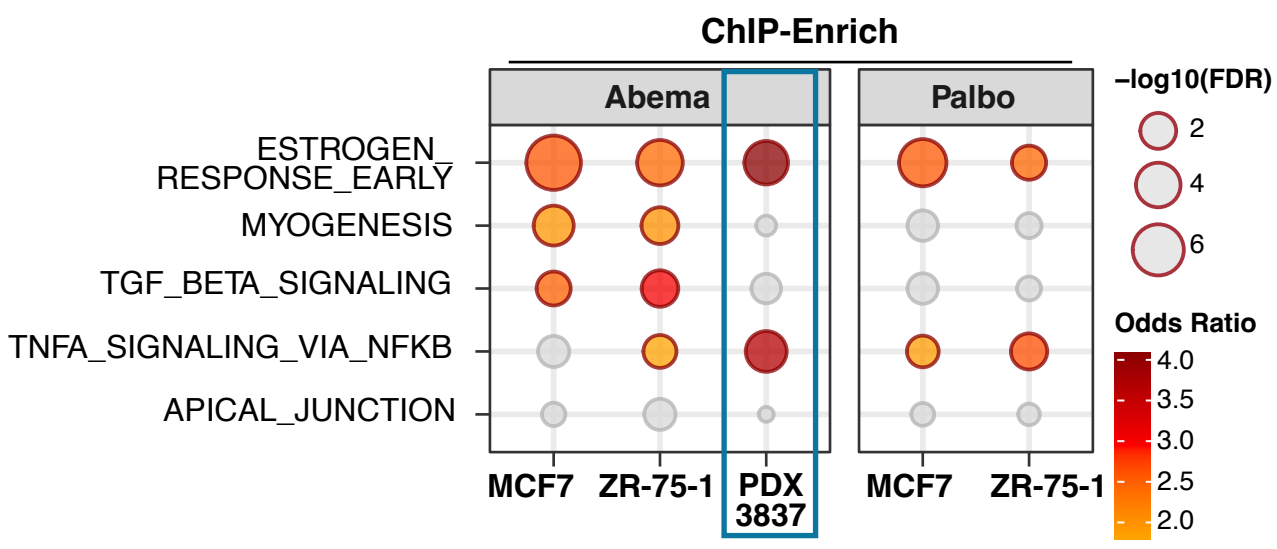

Hallmark gene set enrichment (ChIP-Enrich) for genes nearest to the non-promoter Rb up-peaks in MCF7 and ZR-75-1 cell lines following abemaciclib or palbociclib treatment compared to DMSO, or in PDX KCC-P-3837 following abemaciclib treatment compared to vehicle control. (Fig. 2a in revised manuscript).

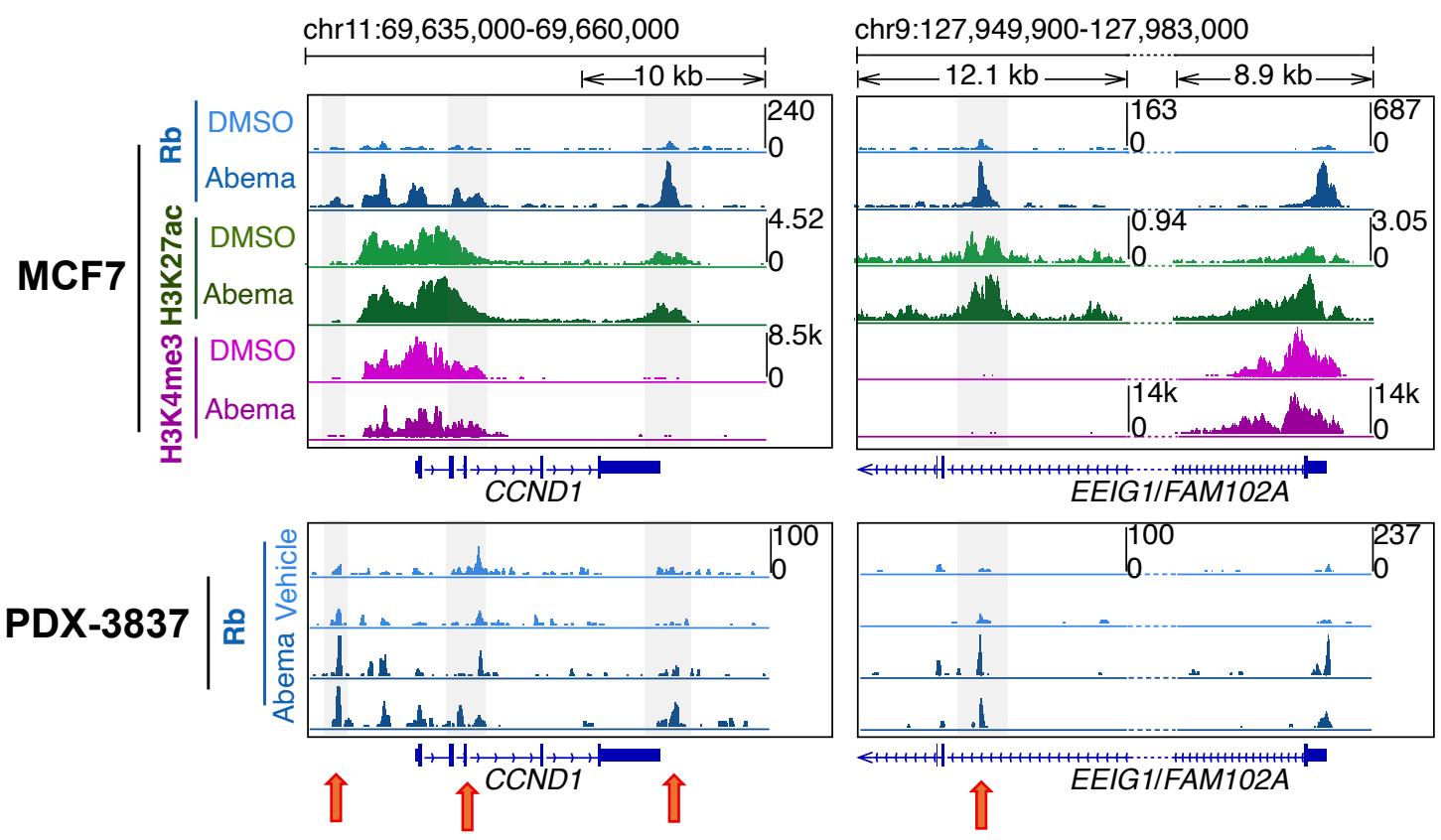

Top: Representative genomic regions near ER target genes in MCF7 cells treated with abemaciclib or DMSO, showing signal from Rb CUT&RUN, H3K27ac ChIP-seq, and H3K4me3 CUT&RUN. Examples are shown of Rb up peaks with increased H3K27ac at distal enhancers, promoter-proximal regions, and broader regulatory domains.

Bottom: Rb DynaTag signal from PDX-3837 treated with abemaciclib or vehicle, at the same ER target gene loci. (Extended Data Fig. 3f in revised manuscript).

Comment 5 continued –

The overall concern here (motivating points 3-5) is that data are being cherry-picked to support the authors' hypothesis, and more evidence is needed to support the generality of RB-activated gene expression that has meaningful consequences.

We thank the reviewer for raising this important concern. To directly address the possibility of selective reporting, we performed a comprehensive and systematic comparison across all gene set enrichment analyses included in this study, spanning both transcriptional and epigenetic datasets. In total, this comprises 17 independent analyses, including RNA expression datasets (two cell lines treated with abemaciclib or palbociclib, a PDX model treated with palbociclib, and clinical samples from the POP trial), as well as multiple analyses of Rb chromatin occupancy (ChIP-Enrich of non-promoter Rb up-peaks across cell lines and PDX models, ORA analyses in MCF-7 and ZR-75-1 cells, HiChIP-based ORA, and hub-based ORA). Across these analyses, *Estrogen Response Early* consistently emerged among the top enriched gene sets (and was the top enriched gene set in 11/17 analyses). This degree of consistency across independent datasets, analytical approaches, and data types indicates that enrichment of estrogen-responsive genes is not a post hoc observation, but a dominant and reproducible feature of the data. Notably, this signal is observed in both gene expression and chromatin-level analyses, linking Rb occupancy at non-promoter regulatory elements to transcriptional output.

This convergence is observed across biological contexts, including multiple ER-positive cell lines, two distinct CDK4/6 inhibitors, tumour tissue, and patient samples, indicating that Rb-associated activation of estrogen-responsive transcription is a general feature of CDK4/6 inhibition in ER-positive breast cancer.

Importantly, our conclusions are not based solely on association. We provide direct functional evidence that Rb is required for this transcriptional program. Genetic ablation of Rb suppresses induction of estrogen response genes following CDK4/6 inhibition. Furthermore, CRISPR-mediated disruption of Rb-bound enhancer elements at representative target genes, including *CCND1* and *KRT18*, reduces their expression under CDK4/6 inhibitor treatment. These experiments demonstrate that Rb binding at non-promoter regulatory elements is not merely correlative, but functionally contributes to gene activation.

Taken together, the convergence of 17 independent analyses across orthogonal data types, combined with direct genetic and locus-specific perturbation experiments, demonstrates that Rb-mediated activation of estrogen-responsive genes is a robust, reproducible, and mechanistically supported feature of CDK4/6 inhibition, rather than a product of selective data presentation.

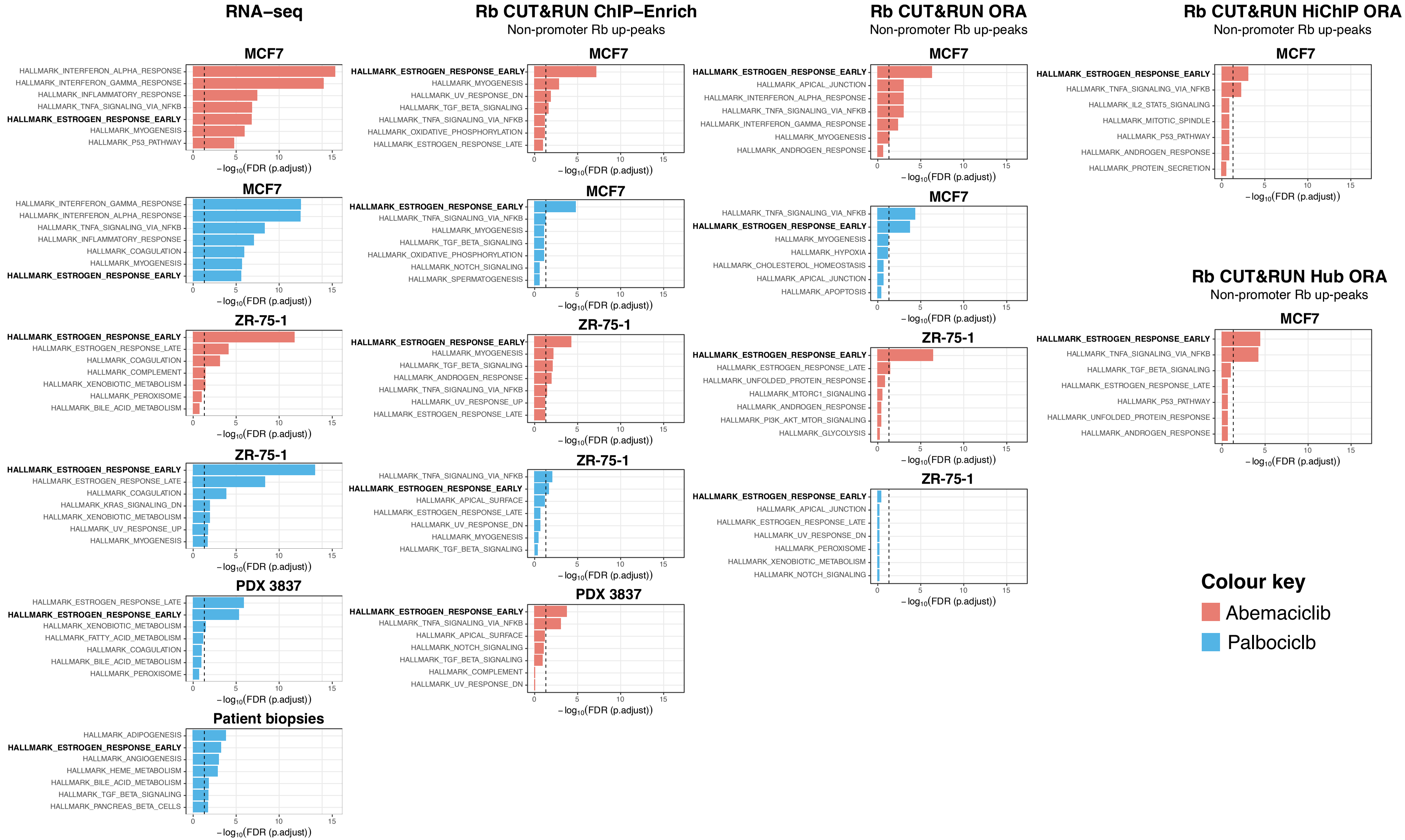

Top 7 enriched gene sets from 17 gene-set enrichment analyses grouped by analytical approach. Columns represent five analytical approaches: RNA-seq (GSEA), ChIP-Enrich, and three RB CUT&RUN-derived methods — ORA, ORA of HiChIP-linked genes, and ORA of hub genes (overrepresentation analysis, ORA). Sample names are indicated in panel titles. Data included here for reviewer's reference.

6) Line 168: Do KTR18 protein levels increase after abemaciclib treatment and does mutation of the enhancer prevent this potential increase? The authors should show a change in protein level (e.g. by Western blot) to demonstrate the significance of the change in mRNA level. Also, considering the importance in the overall model given to activation of CCND1, the authors should perform this experiment mutating the Rb-binding site impacting CCND1 expression, or they should provide an explanation of why it is technically not possible.

We thank the reviewer for this suggestion. We agree that demonstrating changes at the protein level is important, particularly for genes central to our model.

In response, we have now focused on *CCND1*, given its central role in cell cycle regulation and its importance to the overall conceptual framework of the study. In the revised manuscript, we confirm again that CDK4/6 inhibition leads to increased *CCND1* expression at both the mRNA and protein level (Fig. 2e, 3f, and Extended Data Fig. 4a and 4b). **Importantly, we now add new data exploring the impact of CRISPR-mediated disruption of Rb-bound enhancer elements at the *CCND1* locus.** Specifically, we targeted two different Rb-bound enhancers either alone or together (see Figures below). Enhancer disruption did not change *CCND1* expression under basal conditions, but did attenuate the increase in *CCND1* mRNA after CDK4/6 inhibition.

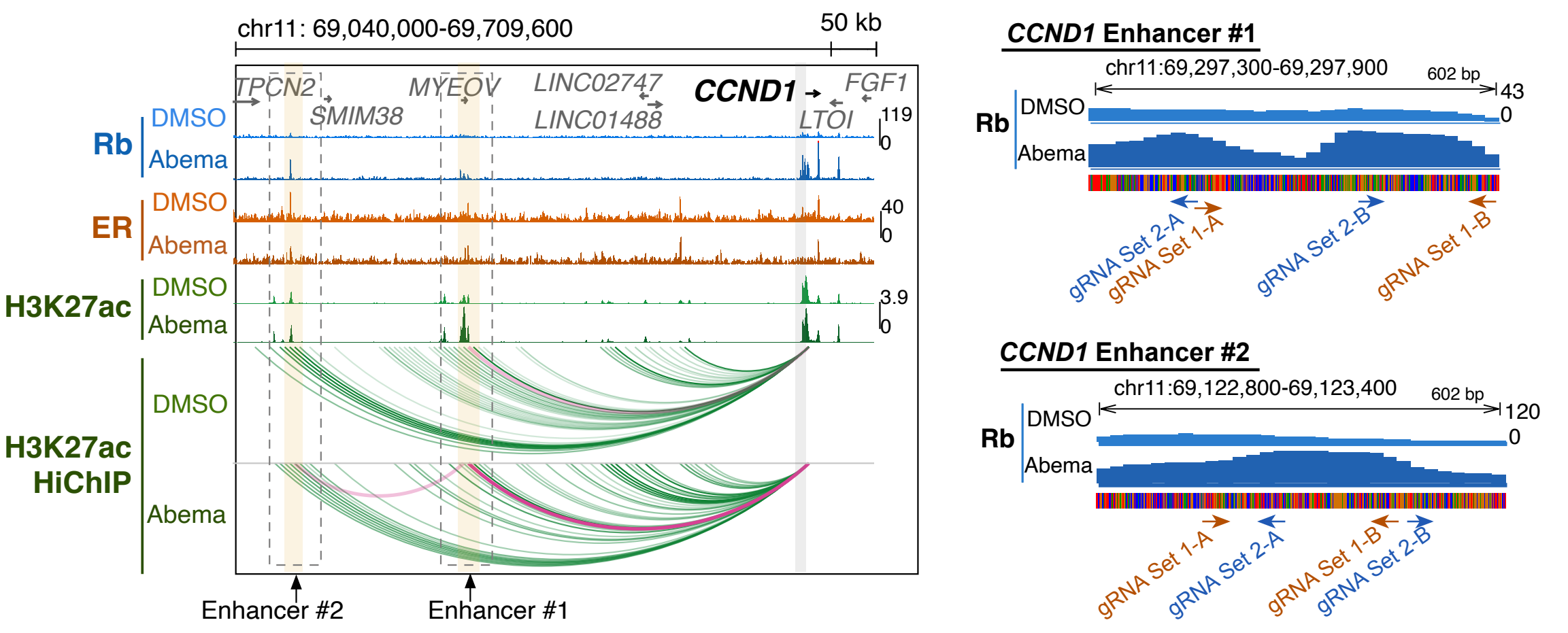

Left: *CCND1* gene loci and non-promoter Rb up peaks that loop to its gene promoter. Tracks shown include signal from Rb CUT&RUN (blue), ER CUT&RUN (orange), H3K27ac ChIP-seq (green), and H3K27ac HiChIP (green and magenta). Grey vertical bands mark ER target gene promoters. Magenta colouring indicates loops that are either newly formed or had more contacts (greater interaction strength) following treatment. The genomic regions in the grey dashed boxes and highlighted in yellow vertical bands contain the Rb non-promoter up peak regions targeted by CRISPR KO (Fig. 2d in revised manuscript).

Right: Illustration of Rb up peak genomic locations that contact the *CCND1* promoter as indicated in the left-side panel and the locations where sgRNAs targeting these regions were directed (Extended Data Fig. 4c).

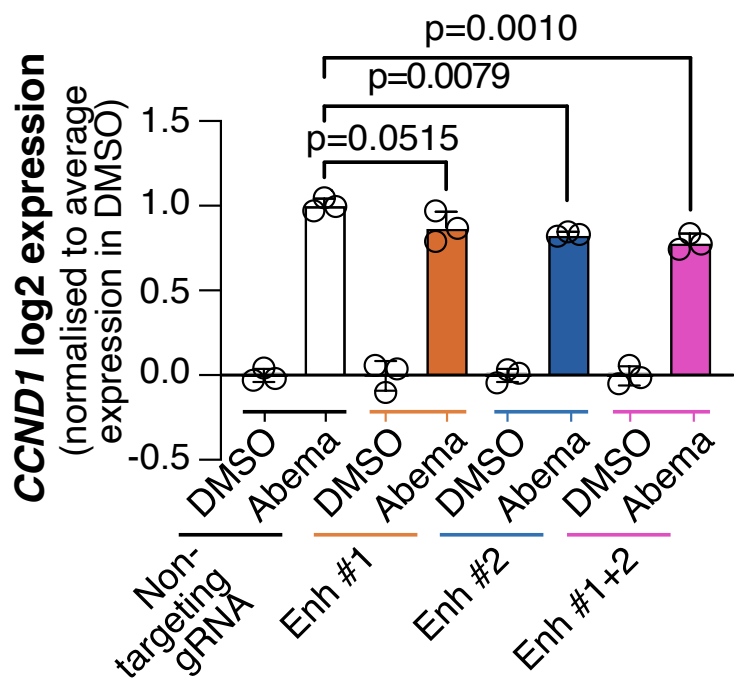

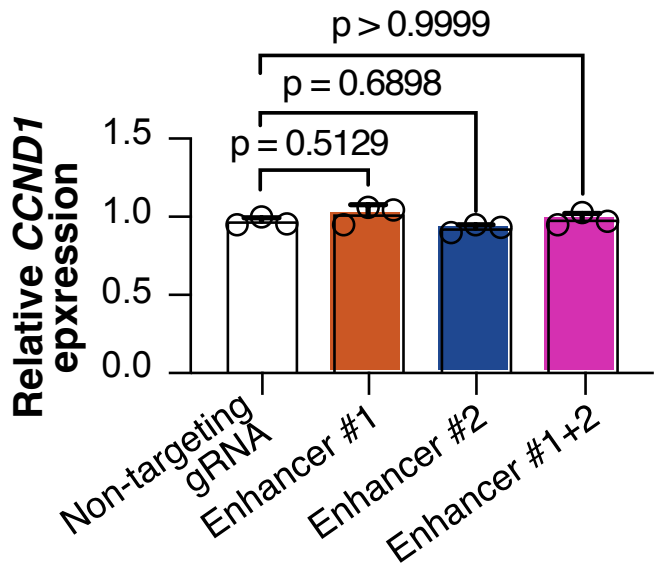

Left: Expression of *CCND1* mRNA in MCF7 cells treated with abemaciclib (5 days) or DMSO (3 days), with concomitant CRISPR-Cas9 knockout using sgRNA targeting either the non-promoter Rb up peak regions described above, or non-targeting sgRNA. Knockout of the enhancers reduced the abemaciclib-induced upregulation of the *CCND1* gene (Fig. 2f in revised manuscript).

Right: Basal (i.e. cells without drug treatment) expression of *CCND1*in DMSO-treated MCF7 cells as in the Left panel (Extended Fig. 4d).

While this attenuation is not complete, it is statistically significant, which is expected given that multiple enhancer elements likely contribute to *CCND1* regulation and only a subset were targeted for technical reasons.

Notably, this effect is reflected at the protein level, further supporting the functional impact of these regulatory elements.

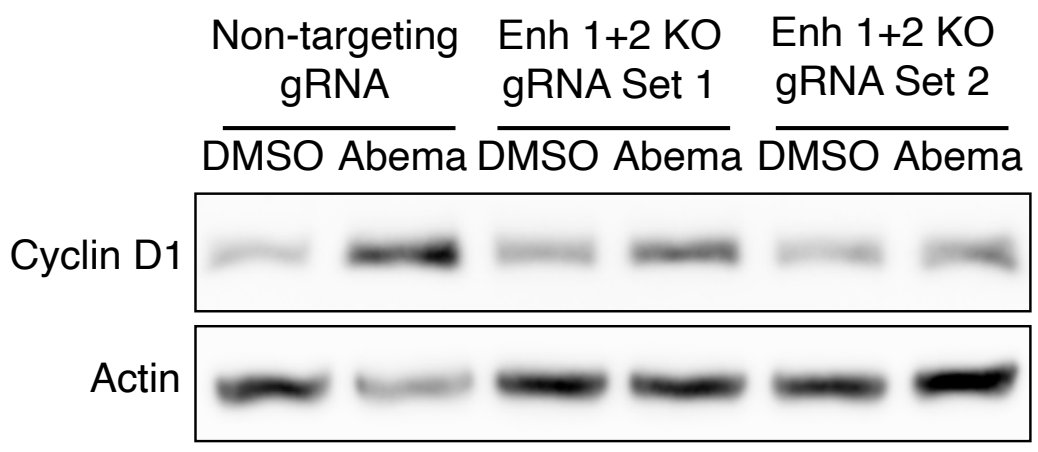

Protein expression of cyclin D1 in MCF7 cells treated with abemaciclib or DMSO, with concomitant CRISPR-Cas9 knockout using two sets of sgRNAs as indicated in the schematics above (Extended Data Fig. 4e).

Together, these data provide direct evidence that Rb binding at non-promoter regulatory elements can drive expression of a canonical pro-proliferative gene. **To our knowledge, this represents the first demonstration that Rb can directly promote expression of a gene that drives cell cycle progression, thereby extending its role beyond transcriptional repression of E2F targets.**

As the reviewer suggested, we also assessed KRT18 at the protein level. Consistent with our transcriptional data, CDK4/6 inhibition led to increased KRT18 protein expression (see response to point 7 below). However, in contrast to the mRNA results, CRISPR-mediated disruption of Rb-bound enhancer elements did not produce a clear reduction in KRT18 protein levels. One possible explanation is that the enhancer perturbation experiments target only a subset of regulatory elements, and the resulting partial reduction in transcription may be insufficient to overcome buffering at the protein level – especially for relatively stable, structural intermediate filament proteins such as cytokeratins. Importantly, the absence of a measurable change in total protein levels in this setting does not negate the biological relevance of the transcriptional effect, particularly given that protein-level consequences are clearly observed for key effector genes such as *CCND1*.

7) **Protein levels of some genes should also be tested to support the mRNA expression studies presented in Figs. 2E-2H and in Extended Fig. 4H.**

We thank the reviewer for this suggestion. To support the mRNA expression analyses, we have now included protein-level validation for representative targets.

Regarding Figs. 2e-h of the original manuscript, we show immunoblotting of cyclin D1, KRT18, and TFF1 in MCF7 parental and *RB1* knockout cells, as well as in ZR-75-1 parental cells. The results show an increase in expression of these ER targets with abemaciclib treatment, which is Rb-dependent and mitigated by endocrine therapy. These results are consistent with the transcriptional changes observed in Figs. 2e-h (Figs. 2e, h, and i in revised manuscript).

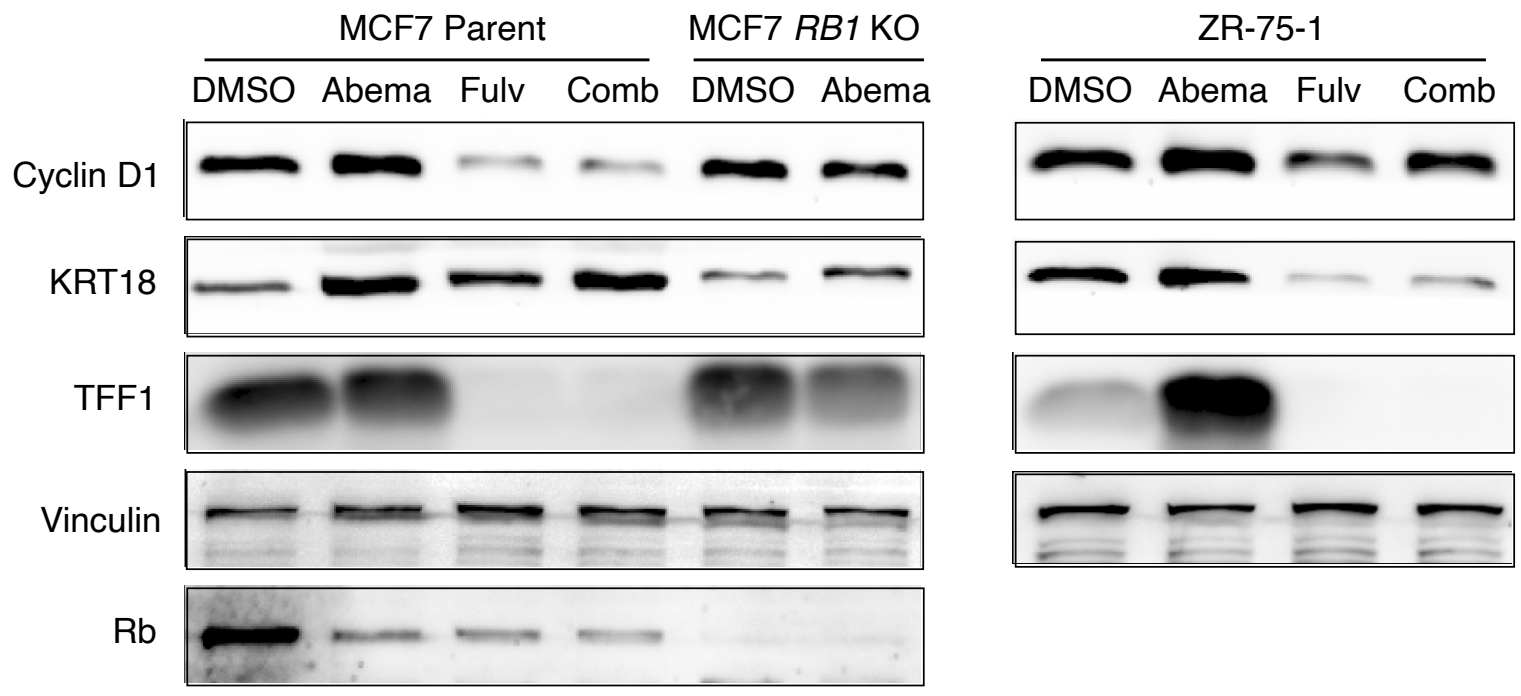

Western blot of ER target genes in MCF7 parent and isogenic *RB1* knockout cells and ZR-75-1 cells following treatment with DMSO, abemaciclib (Abema), fulvestrant (Fulv), or the combination of abemaciclib and fulvestrant (Comb) for five days (Extended Data Fig. 4a).

In Extended Data Fig. 4h of the original manuscript, the combination of abemaciclib with *CCND1* knockdown resulted in a further reduction of the E2F target genes *MKI67* and *TK1* compared to abemaciclib alone, assessed by RT-qPCR. However, detection of MKI67 by immunoblotting is technically challenging due to its large size, and TK1 protein levels were below the limit of detection under our experimental conditions. As an alternative, we assessed Cyclin A2 (*CCNA2*), a well-established E2F-regulated cell cycle protein. Notably, *CCNA2* mRNA expression is consistently downregulated by abemaciclib and fulvestrant treatments (see Extended Data Fig. 5d), supporting its use as a robust protein-level readout of E2F activity. Cyclin A2 protein levels were further reduced upon *CCND1* knockdown in combination with CDK4/6 inhibition.

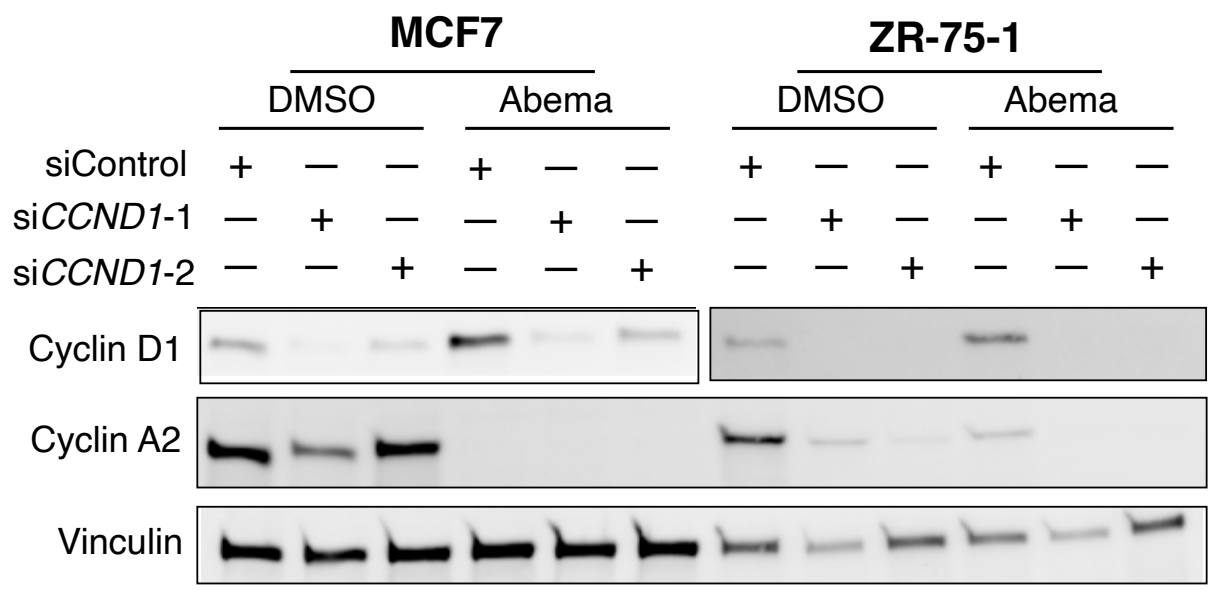

Protein levels of cyclins D1 and A2 in MCF7 and ZR-75-1 cells transfected with control non-targeting or *CCND1* siRNA and treated with DMSO or abemaciclib for 5 days (Extended Data Fig. 6c).

Importantly, the aim of this experiment is not to demonstrate an immediate further decrease in protein abundance, but rather to show that *CCND1* depletion deepens transcriptional suppression of E2F target genes. We interpret this as reinforcing the depth of cell cycle inhibition, thereby limiting the ability of cells to restore protein levels required for proliferation over time.

8) The experiment in Fig. 3C comparing the effects of Abema vs DMSO and Abema+fulv vs Abema on gene expression should also be done in the isogenic Rb KO lines to show Rb dependency of this result.

We thank the reviewer for this thoughtful suggestion. The intent of Fig. 3c is to demonstrate that CDK4/6 inhibition induces an estrogen response transcriptional program in ER+ breast cancer cells, and that this program can be antagonized by pharmacologic inhibition of ER with fulvestrant.

The Rb dependency of the abemaciclib-induced estrogen response is addressed directly in isogenic *RB1* knockout cells (Figs. 2e and 2i, and Extended Data Fig. 4h), where abemaciclib fails to induce the *Hallmark Estrogen Response Early* gene set observed in parental cells. We therefore view this experiment as specifically addressing the requirement for Rb in mediating the transcriptional response to CDK4/6 inhibition.

In contrast, the "Abema+fulv vs Abema" comparison reflects the transcriptional consequences of ER degradation. As fulvestrant acts upstream by directly targeting ER for degradation, its effects on estrogen-responsive transcription would be expected to occur largely independently of Rb status. For this reason, performing this comparison in *RB1* knockout cells may be difficult to interpret in the context of Rb dependency, as it would primarily capture an ER-loss transcriptional program. We include data here, for the reviewer's reference, to illustrate these points.

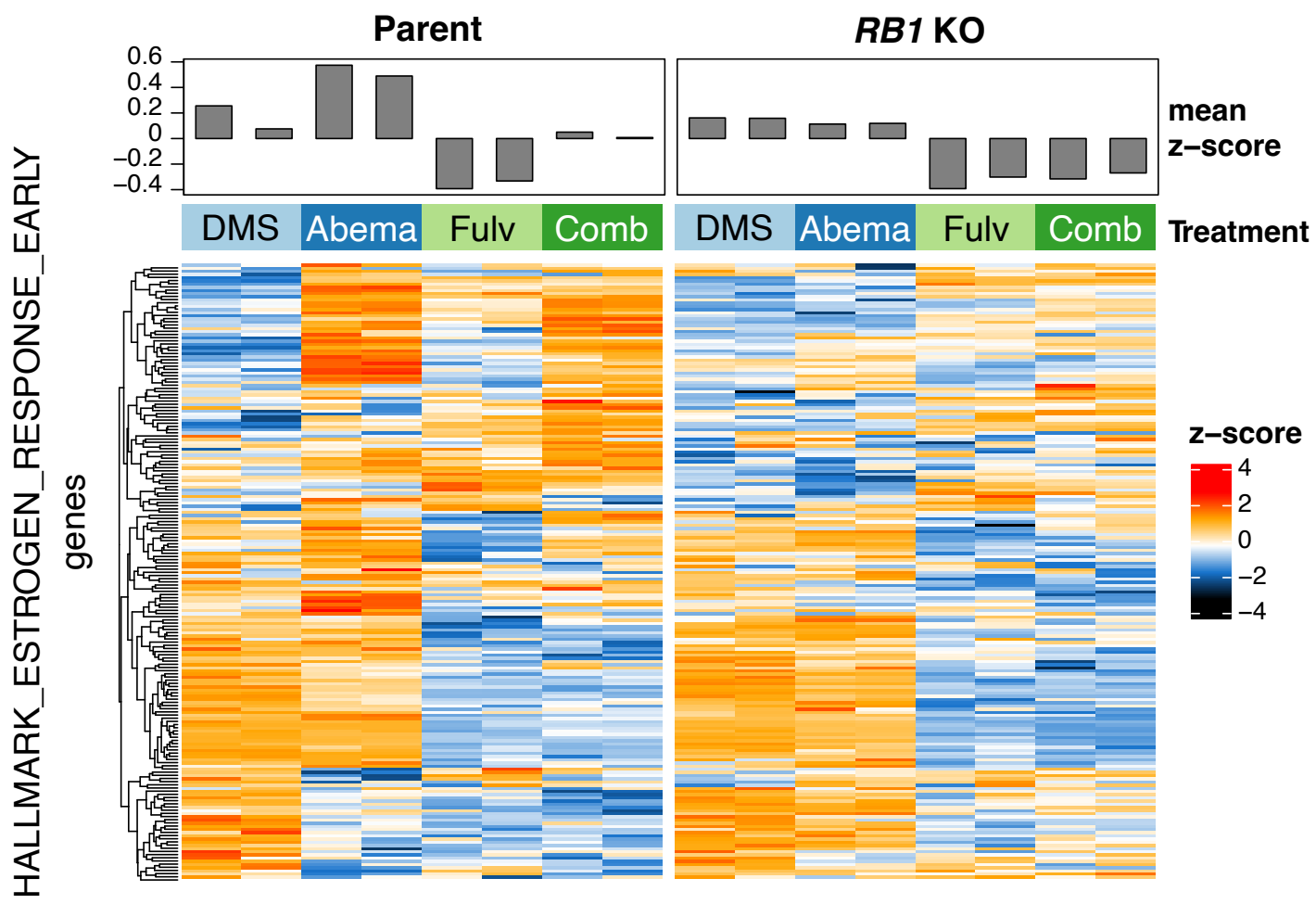

MCF7 parental and isogenic *RB1* knockout cells were treated with drugs shown for 5 days, and RNA-seq was performed. Heatmap shows z-scores for expression of genes from the *Hallmark Estrogen Response Early* gene set. Data included for reviewer's reference.

9) A table listing the interaction partners identified in the mass spectrometry screen presented in Fig. 4A should be included.

We thank the reviewer for this constructive suggestion. We agree that providing the complete list of significantly enriched Rb-interacting proteins from the IP-MS experiment will improve transparency and reproducibility. We have now included a Supplementary Table (Supplementary Table 6) containing the full list of detected proteins, along with associated statistical parameters (e.g., $\log_2$ fold-change, adjusted *p*-values, peptide counts, and replicate information). This dataset includes all significantly enriched interactors identified in the Rb IP relative to IgG IP, and the number of proteins identified is consistent with other published nuclear IP-MS experiments. Given that several classical Rb interactors were identified, this number likely reflects the complexity of nuclear Rb complexes and the depth of the mass spectrometry, rather than a non-specific pull-down.

10) **An immunoprecipitation experiment in an additional cell line should be performed to support the result in Fig. 4B.**

We thank the reviewer for this suggestion. In response, we have performed the immunoprecipitation experiment in an additional ER+ breast cancer cell line (ZR-75-1).

Consistent with our findings in MCF7 cells, we observe interaction between Rb and KDM5A in ZR-75-1 cells. While the total amount of Rb recovered in the abemaciclib condition was lower in this experiment, the level of co-immunoprecipitated KDM5A was maintained, resulting in an increased KDM5A:Rb ratio following CDK4/6 inhibition. This is consistent with our original observation that CDK4/6 inhibition enhances the association between Rb and KDM5A.

We also detect evidence of Rb–ER interaction in ZR-75-1 cells, although this is less robust than in MCF7 cells. These data have been added to Extended Data Fig. 7a and described in the revised manuscript.

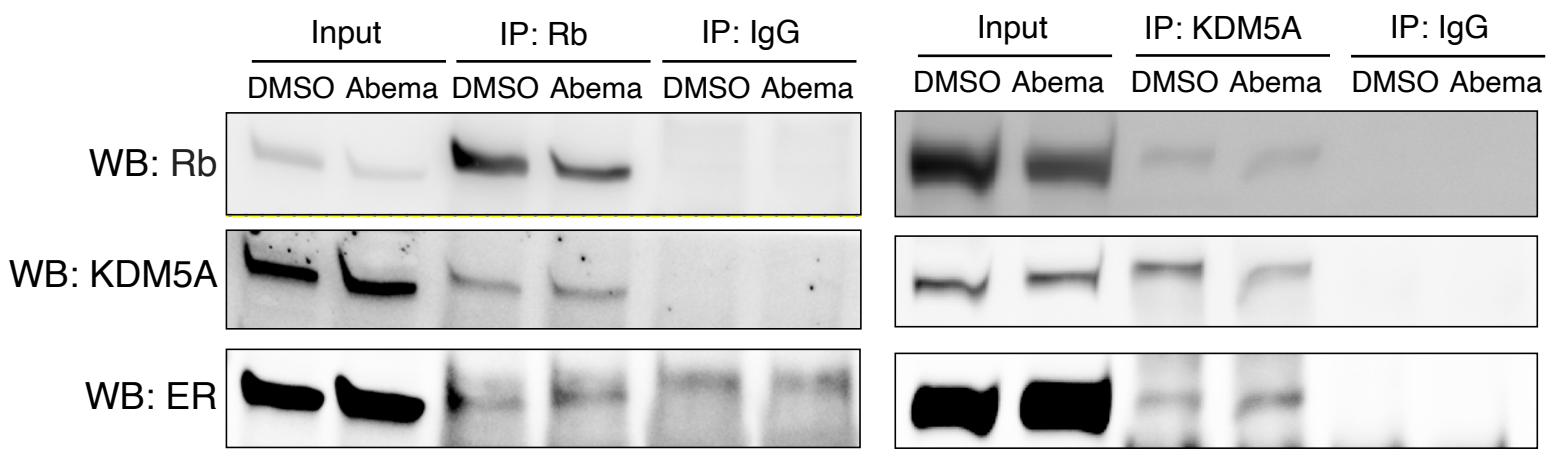

Western blot of protein obtained after Rb (left) or KDM5A (right) IP from nuclear lysates of ZR-75-1 cells treated with DMSO or abemaciclib for 2 days (Extended Data Fig. 7a).

To further strengthen evidence for the interaction between Rb, ER, and KDM5A, we performed **three-way** reciprocal co-immunoprecipitation experiments in MCF7 cells. Pulldown of each protein (Rb, ER, or KDM5A) resulted in co-precipitation of the other two, providing strong evidence for the formation of a shared nuclear complex. These findings reinforce the association identified by IP–MS and support a model in which Rb functions within an ER-KDM5A-containing transcriptional complex following CDK4/6 inhibition.

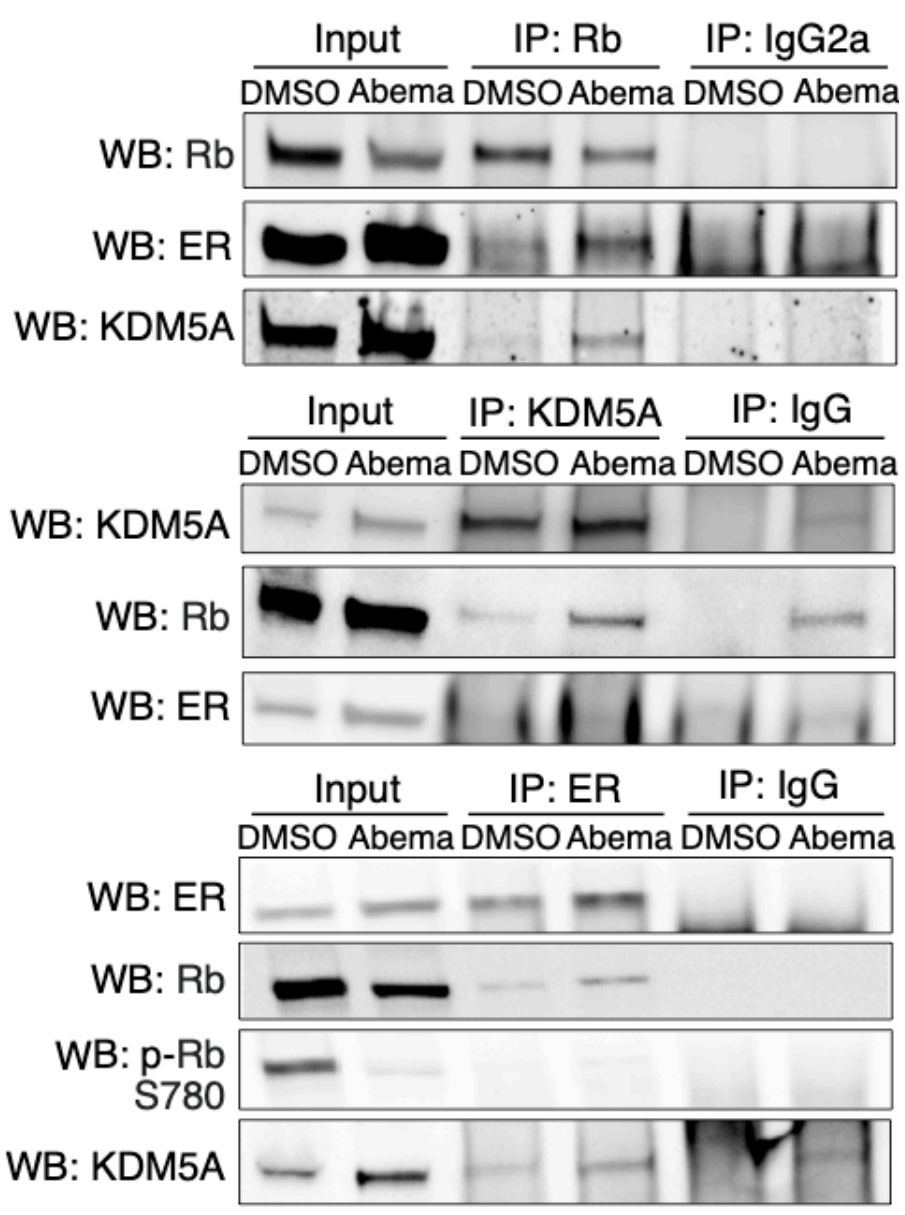

Western blot of protein obtained after Rb (top), KDM5A (middle), or ER (bottom) IP from nuclear lysates of MCF7 cells treated with DMSO or abemaciclib for 2 days. (Fig. 4b).

11) Lines 253-257: The authors should demonstrate that knockdown of KDM5A or ER leads to loss of RB recruitment on enhancers. Although it is implied in the overall model, there is no evidence that either of the proteins recruits RB to the enhancers. Does ER recruit KDM5A or does KDM5A bind to DNA directly, as shown in (PMID: 18270511)? Overall, the concern here is that characterization of the complex is insufficient and that the model is insufficiently developed with respect to how the specificity of RB recruitment at these sites and in response to drug treatment is achieved.

We thank the reviewer for this important point regarding the mechanism of Rb recruitment. To address this directly, we performed *KDM5A* knockdown followed by assessment of Rb chromatin binding after CDK4/6 inhibition. Notably, we did not observe a reduction in Rb recruitment under these conditions.

This finding suggests that KDM5A is not required for the initial recruitment of Rb to chromatin. Consistent with this, our data indicate that KDM5A occupancy is largely unchanged following CDK4/6 inhibition, supporting a model in which KDM5A is present at these sites prior to treatment. In this context, CDK4/6 inhibition promotes recruitment of Rb to chromatin, where it subsequently associates with pre-bound KDM5A.

We therefore interpret our data as distinguishing between recruitment and complex formation: while the Rb–KDM5A interaction is enhanced following CDK4/6 inhibition, KDM5A does not appear to mediate Rb recruitment to these sites. The factor(s) responsible for directing Rb to specific genomic loci remain to be defined.

We agree that a full dissection of the components governing Rb recruitment and site specificity will be an important area for future work. However, we consider this to be beyond the scope of the current study, which focuses on defining the functional consequences of Rb chromatin binding following CDK4/6 inhibition. We have clarified this point in the revised manuscript.

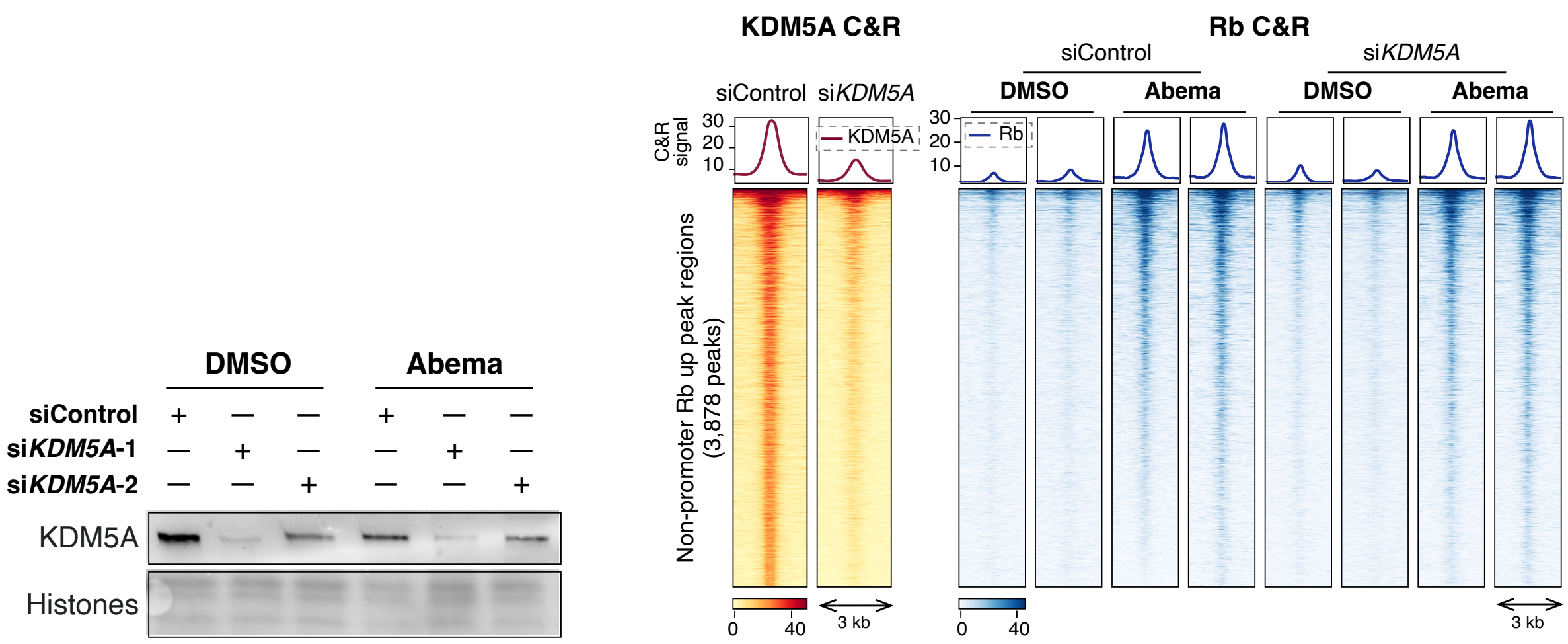

Left: Western blot transfected with control or KDM5A siRNA and treated with DMSO or abemaciclib (Extended Data Fig. 7c).

Right: MCF7 cells were treated with control or si*KDM5A*-1 siRNA. CUT&RUN was performed for KDM5A in these cells (left; yellow-red). Rb CUT&RUN was performed on these cells with the addition of DMSO or abemaciclib treatment for 2 days. This heatmap shows the CUT&RUN signal from cells treated with DMSO or abemaciclib in addition to siRNA (right; blue) at Rb non-promoter up peaks regions as in Fig. 1c.

12) Fig. 4g: This result needs to be supported by qPCR and Western blot for a few ER target genes, including CCND1. Also, the authors should report how many of the 405/17 genes described in lines 157-163 are upregulated in DMSO/siKDM5A-treated cells.

We thank the reviewer for this suggestion. We agree that it is important to more clearly define the effect of KDM5A depletion on ER target genes highlighted in our model. To address this, we now include additional analyses of the 17 genes identified in MCF7 cells as estrogen-responsive targets with non-promoter Rb peaks looping to their promoters. As shown in the new heatmap, the majority of these genes show upregulation after *KDM5A* knockdown. When looking at all 344 genes with promoters linked to the 405 Rb non-promoter up-peaks, we also observe a trend towards upregulation of genes with *KDM5A* knockdown, but this was less obvious. Critically, the focus of our work is on the importance of Rb/KDM5A as regulators of ER target gene expression, and it is thus noteworthy that the most consistent upregulation is seen in those genes belonging to the *Hallmark Estrogen Response Early* gene set. Because these effects were assessed transcriptome-wide by RNA-seq, we used these genome-wide data as the principal transcript-level validation, together with protein-level confirmation for representative targets.

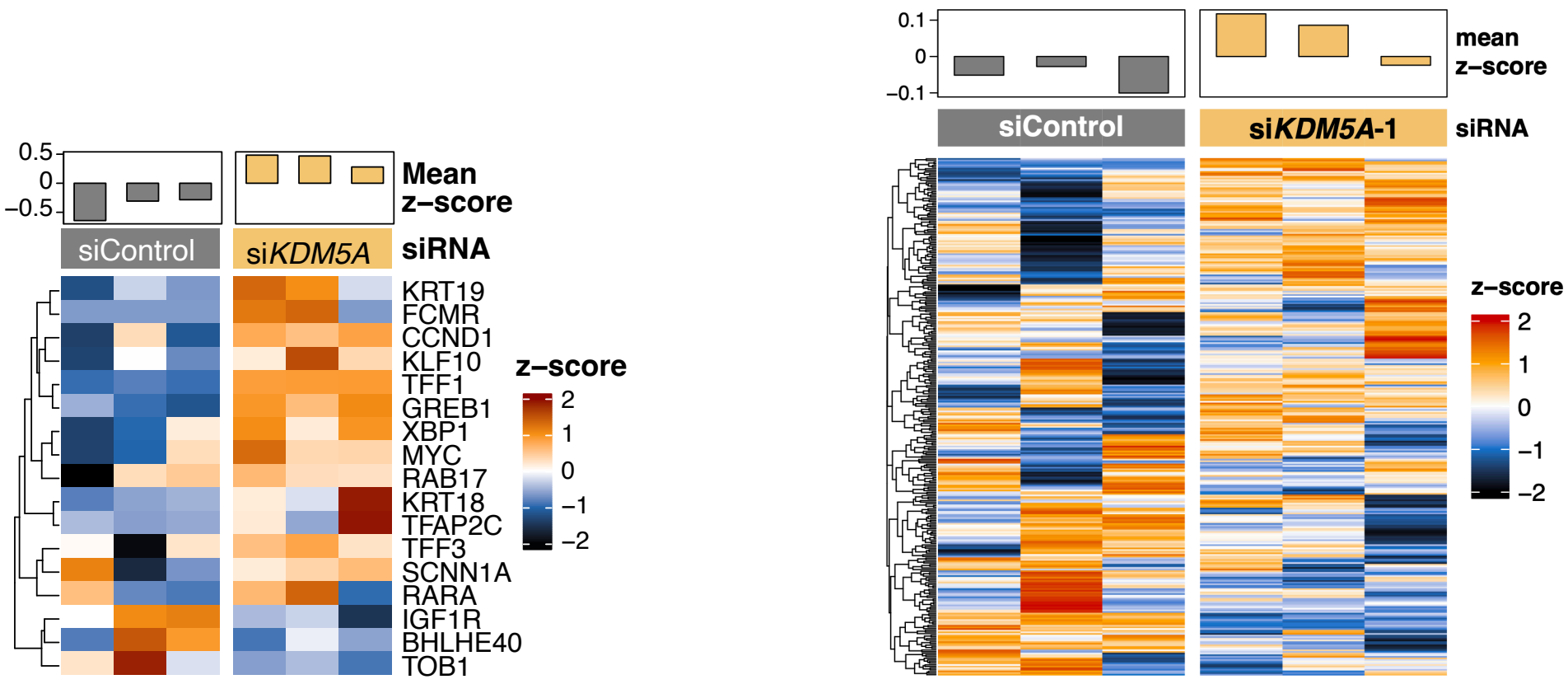

Left: Heatmap showing relative expression of 17 genes from the *Hallmark Estrogen Response Early* gene set in MCF7 siControl and si*KDM5A* cells. These 17 genes are a subset of the 344 genes identified as targets of non-promoter Rb up-peaks looping to their promoters (MCF7 H3K27ac HiChIP), which overlapped with the *Hallmark Estrogen Response Early* gene set. Bar plots show mean z-score per sample (Extended Data Fig. 7e).

Right: Heatmap showing relative expression of all 344 genes identified as targets of non-promoter Rb gained peaks looping to their promoters in MCF7 (H3K27ac HiChIP). Bar plots show mean z-score per sample. Data included for reference.

In addition, we now include western blot validation for key ER targets, including cyclin D1 and KRT18**,** both of which show increased protein expression following *KDM5A* knockdown. As expected, this increase is less evident in KDM5A-depleted cells treated with abemaciclib, consistent with our model that *KDM5A* knockdown phenocopies the de-repressive effect of CDK4/6 inhibition on this transcriptional program.

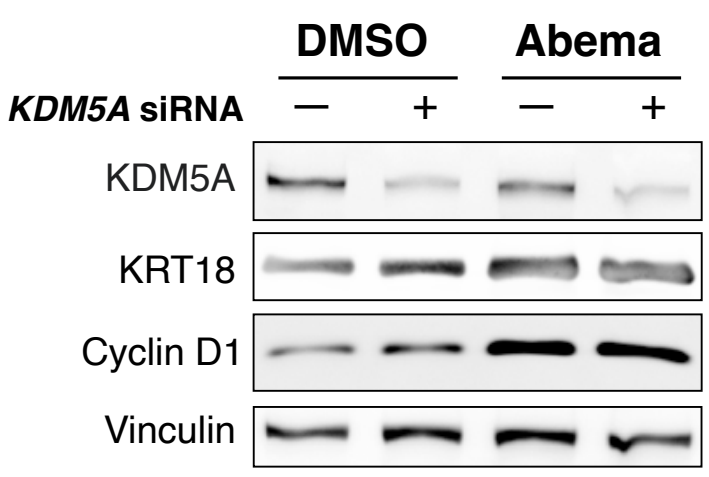

Western blot of MCF7 cells transfected with control or *KDM5A* siRNA and treated with DMSO or abemaciclib for 3 days (Extended Data Fig. 7f).

Together, these new data strengthen the conclusion that KDM5A acts as a repressor of this Rb-associated ER transcriptional response, and that relief of KDM5A-mediated repression contributes to induction of functionally relevant target genes.

References

Gala, K., Q. Li, A. Sinha, P. Razavi, M. Dorso, F. Sanchez-Vega, Y. R. Chung, R. Hendrickson, J. J. Hsieh, M. Berger, N. Schultz, A. Pastore, O. Abdel-Wahab and S. Chandarlapaty (2018). "KMT2C mediates the estrogen dependence of breast cancer through regulation of ERalpha enhancer function." Oncogene **37**(34): 4692-4710.

Hunold, P., G. Pizzolato, N. Heramvand, L. Kaiser, G. Barbiera, O. van Ray, R. Thomas, J. George, M. Peifer and R. Hansel-Hertsch (2025). "DynaTag for efficient mapping of transcription factors in low-input samples and at single-cell resolution." Nat Commun **16**(1): 6585.

Response to Referees

Rb-driven transcription limits its tumour suppressive effects in breast cancer (Manuscript ID: 2025-08-22316B).

We thank the reviewers for their constructive feedback on our revised manuscript. We have made further modifications as outlined below.

<u>**Response to Referee #1**</u>

I read the revised manuscript and the related supplementary material with interest. The paper has been significantly improved by this revision. I think this version includes all the requested changes I had previously made. Congratulations to the Authors for this important work.

We thank the reviewer for their comments and feedback on our initial manuscript. Incorporation of the reviewer's suggestions has strengthened our paper, and we are pleased that the reviewer agrees.

<u>**Response to Referee #2**</u>

The authors have strengthened the manuscript in response to reviewers' comments. However, I disagree with some of the interpretation of the response to reviewer 2 point 1 and the text and supplementary figure 5b,c. The treatment with abemaciclib does induce an oestrogenic transcriptional programme but this is not inhibited by fulvestrant, perhaps unsurprisingly in the demonstrated absence of induction of the ESR1 transcript in an ER negative cell line. This needs greater clarity in the text as the implication from the new data is that the recruitment of Rb to the hubs is NOT ER dependent, at least not in its entirety. I note reviewer 3 (point 11) requested either KDM5A or ER knockdown an the authors undertook the former demonstrating no change in the Rb recruitment. I need to see the knockdown of ER to confirm whether the effect is entirely ER mediated, given the data from ER negative cell lines. Otherwise no additional issues with the response

We thank Reviewer 2 for their thoughtful comments and for acknowledging that the manuscript has been substantially strengthened during revision. We agree that the findings in the ER-negative models require careful interpretation and appreciate the opportunity to further clarify the conclusions that can and cannot be drawn from these data.

Reviewer 2 raises two related points: (i) whether the induction of *Hallmark Estrogen Response Early* genes following abemaciclib treatment is entirely ER-dependent; and (ii) whether ER is required for recruitment of Rb to these enhancer-associated chromatin regions.

<u>Assessing the functional contribution of ER in our experimental models</u>
The reviewer is reasonably concerned that enrichment of *Hallmark Estrogen Response Early* genes may not necessarily indicate strict ER dependence. We agree that the data do not support a model in which all transcriptional responses following CDK4/6 inhibition are exclusively mediated by ER. Indeed, this was not our intention. Rather, our central conclusion is that in ER-positive luminal breast cancer models, activated Rb promotes enhancer-associated transcriptional programs that are substantially enriched for ER-responsive genes and are strongly influenced by intact ER signalling.

Several observations support this interpretation.

1. In ER-positive models, pharmacologic ER degradation with fulvestrant (which directly interrogates the functional requirement for ER) markedly attenuates the induction of oestrogen-responsive transcriptional programs following CDK4/6 inhibition (Fig 1, top). Furthermore, when these genes are inhibited by ER degradation, the addition of a CDK4/6 inhibitor is not able to restore their upregulation

(Fig 1, bottom). We believe this remains the strongest evidence that ER signalling is a major functional contributor to these transcriptional responses in ER-positive breast cancer cells.

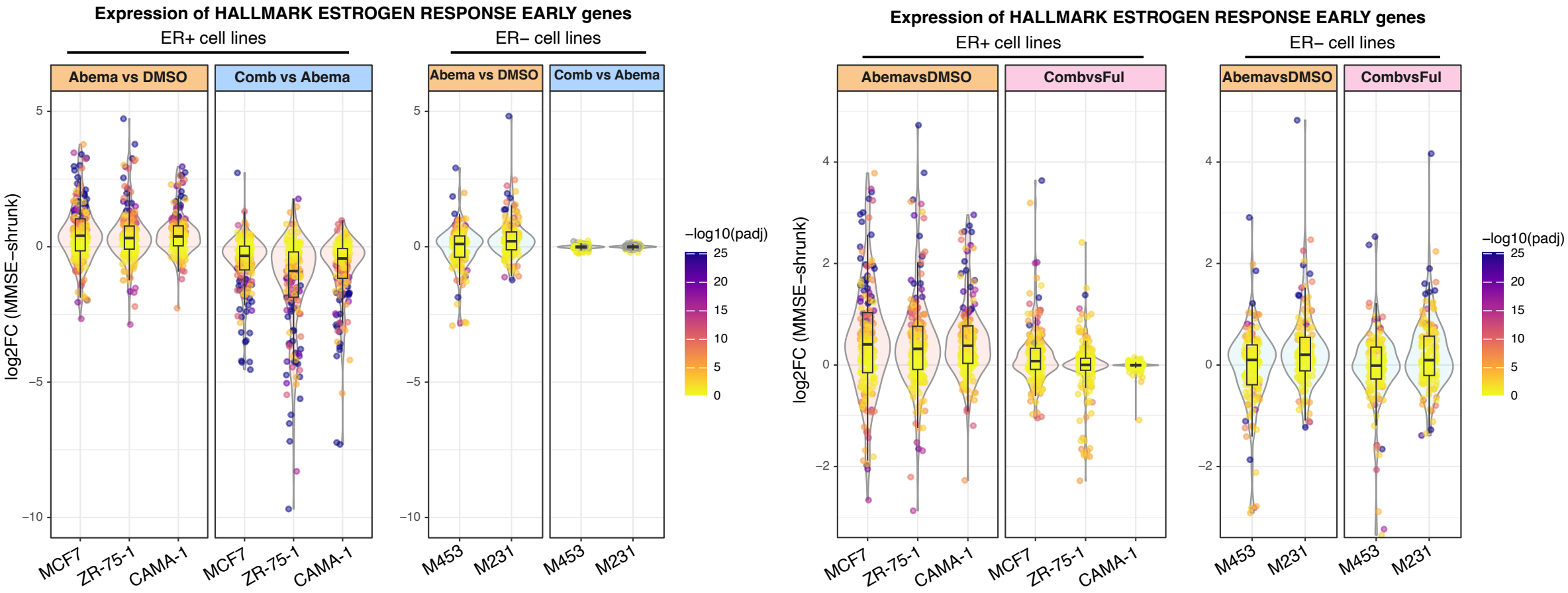

Figure 1. Expression of *Hallmark Estrogen Response Early* genes showing (i) abemaciclib vs. DMSO, (ii) abemaciclib + fulvestrant vs abemaciclib, and (iii) abemaciclib + fulvestrant vs fulvestrant, in ER-positive and ER-negative cell lines.

2. With respect to the induction of *Hallmark Estrogen Response Early* genes in the ER-negative models, we note that this is considerably weaker and involves fewer genes than that observed in ER-positive luminal models (Figure 1 and Figure 2). We agree that the addition of fulvestrant to these cells does not attenuate this effect, and fully acknowledge that additional transcription factors or chromatin-associated mechanisms likely also contribute to transcriptional activation at these loci. Indeed, it is possible that these additional factors might also operate in ER-positive cells, although the fulvestrant data strongly suggest that ER signalling is the dominant contributor in that setting.

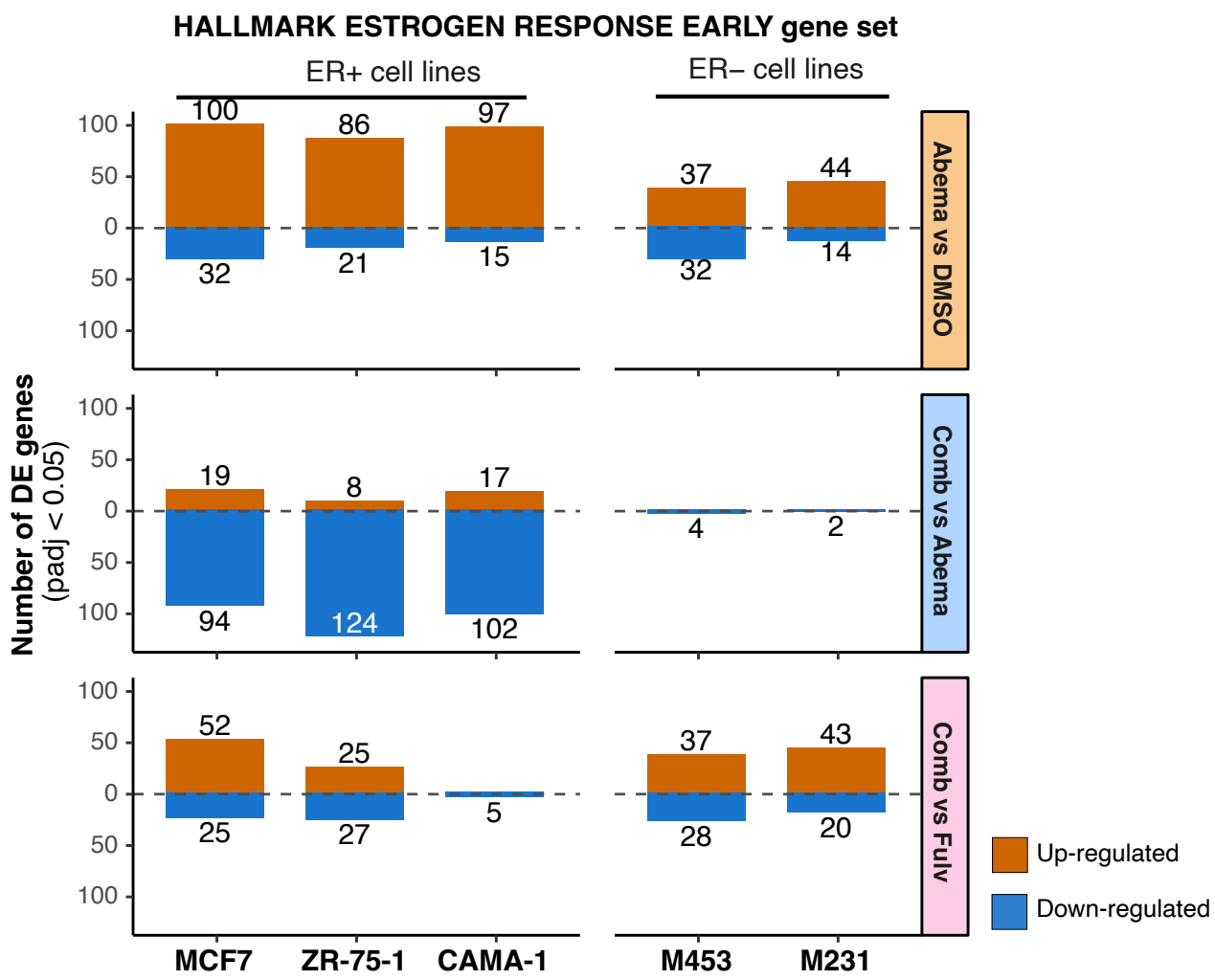

Figure 2. Number of differentially expressed *Hallmark Estrogen Response Early* genes (FDR $p < 0.05$) in ER-positive and ER-negative cell lines for three treatment comparisons: abemaciclib vs. DMSO (top), abemaciclib + fulvestrant (Comb) vs. abemaciclib (middle), and abemaciclib + fulvestrant (Comb) vs. fulvestrant (bottom).

<u>Assessing the contribution of ER in recruiting Rb to the chromatin</u>

We would like to clarify that our model does not suggest an ER-dependent recruitment of Rb to chromatin. Rather, our data support a model in which CDK4/6 inhibition induces Rb chromatin occupancy, and in ER-positive cells, this Rb-chromatin interaction intersects with ER activity to regulate a subset of ER-associated transcriptional outputs. Specifically, our data demonstrate that Rb, ER, and KDM5A exist within a shared chromatin-associated complex in the context of CDK4/6 inhibition.

ER is known to participate in large multi-protein chromatin regulatory assemblies, and it remains entirely plausible that additional transcription factors, pioneer factors, co-regulators, or chromatin-associated proteins contribute to Rb localisation at these loci. We therefore believe that defining the precise hierarchy

and mechanism of recruitment within these complexes represents an important future direction, but one that is beyond the scope of the current study.

With respect to the suggestion that ER knockdown experiments should be performed, we respectfully believe that this experiment would not substantially extend the mechanistic conclusions beyond the fulvestrant data already included in the manuscript. Fulvestrant both antagonises and degrades ER protein and produces robust suppression of the oestrogen-responsive transcriptional program in ER-positive models following CDK4/6 inhibition. Functionally, this already demonstrates that intact ER signalling contributes substantially to the transcriptional responses induced by CDK4/6 inhibition in ER-positive luminal models. Given that we do not claim that ER is required to recruit Rb, an ER knockdown experiment would not be helpful in this context either.

To address the reviewer's concerns, we have done the following:

1. **We have included the following text in our Discussion: "Although ER signalling is a major contributor to this response in luminal breast cancer cells, our data do not establish that ER recruits Rb to chromatin, and additional transcription factors or chromatin-associated regulators may also participate".**
2. **Changed our description of the data from ER-negative cells in the main text as follows: "The importance of ER signalling in this response was further supported by the markedly attenuated induction of *Hallmark Estrogen Response Early* genes in RB1 wild-type, ER-negative cell lines treated with abemaciclib".**
3. **We have replaced the heatmaps in our previous submission with the violin plots shown above (Figure 1 in this document, which is now included as Extended Data Figures 5b and c in the new submission).**

We hope the reviewer will agree that, taken together, the current data strongly support the central conceptual conclusions of the study. More broadly, our study provides one of the first native genome-wide maps of hypophosphorylated Rb binding in cancer cells and, to our knowledge, the first chromatin mapping of activated endogenous Rb in tissue. These analyses reveal an unexpected duality in Rb biology following CDK4/6 inhibition: alongside canonical repression of E2F-driven cell-cycle genes, activated Rb also participates in enhancer-associated transcriptional programs linked to oestrogen-responsive signalling in luminal breast cancer cells. Importantly, these findings suggest that activated Rb can simultaneously contribute to both anti-proliferative and proliferation-associated transcriptional outputs, helping to explain the biological complexity of CDK4/6 inhibitor responses in breast cancer.

Response to Referee #3

The authors have sufficiently addressed a number of concerns, and we remain impressed with the overall novelty of the study, its significance, and the evidence supporting a number of conclusions. We have a couple of remaining points that should be addressed before publication:

We thank the reviewer for their feedback and are pleased that they find our revised manuscript to be improved.

1) The Venn diagrams provided to the reviewer should be included as Supplementary figures in the manuscript to increase transparency. This includes (1) the intersection of significantly upregulated genes among MCF-7 and ZR-75-1 within the set of non-promoter Rb up-peaks (shown on page 16 of the reviewer response); and (2) the intersection of significantly upregulated genes among MCF-7, ZR-75-1, and CAMA-1 cells within the set of genes that form loops with Rb non-promoter up-peaks (shown on page 18 of the reviewer response). Here the authors should also clarify that the set of 18 genes that is the intersection of all three gene sets includes the 17 estrogen response genes highlighted in the manuscript.

We have included both of these Venn Diagrams in the Extended Data (Extended Data Figs. 3c and 4a) and believe the text reflects the data as indicated.

2) Based on the minimal differences observed in the Rb-KDM5a IP with addition of abemaciclib in the new Extended Data Fig 7a, the authors should at least acknowledge in the main text the alternative interpretation that this effect could be a specific feature observed in MCF7 cells.

We acknowledge the reviewer's point, and have added the following sentence to the main text of our new submission: "Although enhancement of these interactions following CDK4/6 inhibition was more pronounced in MCF7 cells than in ZR-75-1 cells, the complex was detectable in both models".
